# Bioactivity in *Rhododendron*: A Systemic Analysis of Antimicrobial and Cytotoxic Activities and Their Phylogenetic and Phytochemical Origins

**DOI:** 10.3389/fpls.2017.00551

**Published:** 2017-04-13

**Authors:** Anne Grimbs, Abhinandan Shrestha, Ahmed S. D. Rezk, Sergio Grimbs, Inamullah Hakeem Said, Hartwig Schepker, Marc-Thorsten Hütt, Dirk C. Albach, Klaudia Brix, Nikolai Kuhnert, Matthias S. Ullrich

**Affiliations:** ^1^Department for Life Sciences and Chemistry, Jacobs University BremenBremen, Germany; ^2^Stiftung Bremer RhododendronparkBremen, Germany; ^3^Institute for Biology and Environmental Sciences, Carl von Ossietzky University OldenburgOldenburg, Germany

**Keywords:** *Rhododendron*, bioactive compounds, phytochemical profiling, antimicrobial activity, cytotoxicity, phylogeny, categorical correlation analysis

## Abstract

The exceptional diversity of the genus *Rhododendron* has a strong potential for identification, characterization, and production of bioactive lead compounds for health purposes. A particularly relevant field of application is the search for new antibiotics. Here, we present a comparative analysis of nearly 90 *Rhododendron* species targeted toward the search for such candidate substances. Through a combination of phytochemical profiles with antimicrobial susceptibility and cytotoxicity, complemented by phylogenetic analyses, we identify seven potentially antimicrobial active but non-cytotoxic compounds in terms of mass-to-charge ratios and retention times. Exemplary bioactivity-guided fractionation for a promising *Rhododendron* species experimentally supports in fact one of these candidate lead compounds. By combining categorical correlation analysis with Boolean operations, we have been able to investigate the origin of bioactive effects in further detail. Intriguingly, we discovered clear indications of systems effects (synergistic interactions and functional redundancies of compounds) in the manifestation of antimicrobial activities in this plant genus.

## Introduction

The World Health Organization declared antibiotic resistance a public health emergency of yet unknown proportions (WHO, 2011). Emerging resistance of pathogens to treatment with antibiotics is responsible for >30,000 deaths per year in the European Union alone, with worldwide incidences predicted to be orders of magnitude higher. Despite the recognized need for new antimicrobials, only two new classes of antibiotics have been brought to market in the last decades (Gwynn et al., 2010). Plants have been used in traditional medicine for more than 5,000 years (Petrovska, 2012), and even today more than 60% of the world's population depends on medicinal plants as their primary source of healthcare. In excess of 300,000 different plant species have so far been cataloged by botanists around the globe (Angiosperm Phylogeny Group, 2016), and 25% of all commercial pharmaceutical drugs are based on plant natural products (Cragg et al., 1997; Cragg and Newman, 2013). Thus, plants offer an immense resource of chemical diversity to be exploited in pharmaceutical research and drug discovery, in particular in the search for novel antibiotics (Harvey et al., 2015).

Traditional natural product chemistry relies on selection mostly based on ethno-pharmacological or epidemiological knowledge of a biological target followed by plant extract screening and activity-guided fractionation. Here, we choose an alternative route to the serendipity-based and laborious traditional approach. We suggest combining metabolomics data with biological activity data, subjected to a detailed statistical analysis and demonstrate the value of such a novel approach in the identification of compounds displaying biological activity. The genus *Rhododendron* has been chosen as a case study since *Rhododendron* extracts are still used in a large number of ethno-medical applications (Popescu and Kopp, 2013). With more than 100 compounds in clinical trials as of late 2009, an increasing number of natural product-based drugs have been approved for clinical use (Li and Vederas, 2009). So far, secondary metabolites from *Rhododendron* have not been used clinically. However, previous phytochemical characterizations showed that proanthocyanins, beneficial to cardiovascular health, occur in high abundance and diversity in *Rhododendron* extracts (Jaiswal et al., 2012). Taken additionally into account the diversity of the genus, which comprises in excess of 1,000 species (Argent, 1997) and is one of the most species-rich plant genera worldwide (Frodin, 2004), give rise to the hypothesis *Rhododendron*'s phytochemical fingerprint has profound potential as a source of bioactive compounds for use as food additives or in medicine (Rønsted et al., 2012). Of particular relevance as *Rhododendron*-derived bioactive compounds are amongst others proanthocyanidins and polyhydroxylated diterpenes such as grayanotoxins (Jaiswal et al., 2012; Popescu and Kopp, 2013).

While for several species, various antimicrobial activities had been reported (Popescu and Kopp, 2013), chemical compounds responsible for a certain bioactivity had been identified only for individual cases (Zhou et al., 2014; Li et al., 2015). Here, we aim at predicting candidate compounds with antimicrobial activity from a large compilation of *Rhododendron* leaves. Our previous studies have shown that antimicrobial activity against Gram-positive bacteria seem to be more pronounced than against Gram-negative bacteria, especially *Bacillus subtilis* (*B. subtilis*) as most-susceptible tester organism (Rezk et al., 2015b). In line with this, the well-established model system for Gram-positive bacteria, *B. subtilis*, was chosen as representative test microorganism for the current study.

Indispensable for potential antibiotic agents is displaying low cell toxicity to ensure safe human application. To this end, additionally the cytotoxicity toward mammalian cell lines of numerous *Rhododendron* crude leaf extracts has been investigated. In one of our previous studies, it was shown that the *Rhododendron*'s cytotoxicity toward epidermal keratinocytes and intestine epithelial cells can be assessed by testing for their effects on metabolic activities and proliferation rates (MTT assay) of cultured mammalian cells *in vitro* (Rezk et al., 2015a). The MTT assay thus provides a means of rapid high-throughput screening with good approximation potential in predicting cytotoxic effects of *Rhododendron*-derived extracts using an easily performed and well-standardized cell viability assay (Karar et al., 2016).

To align the specific bioactivity effects to a particular chemical compound the phytochemical profiles of all *Rhododendron* extracts have been determined using liquid chromatography coupled to mass spectrometry (LC-MS) focusing on secondary metabolites within the methanolic crude extracts. Our previous phytochemical characterization had shown that there are at least two main classes of bioactive substances in *Rhododendron*-derived leaf extracts, polyphenolics and terpenes (Jaiswal et al., 2012). Apart from classical approaches such as principal component analysis and boxplots, categorical correlation analysis had been performed to explore putative bioactive compounds.

Furthermore, intricate biological responses, such as bioactivity, are often assigned to more than a single substance. In drug discovery and development, compound combinations have recently demonstrated their merits as potent pharmaceuticals across a wide range of disease areas compared to treatment with specific agents (Katouli and Komarova, 2010; Tan et al., 2012; Yuan et al., 2012; Hill et al., 2013; Lewis et al., 2015). For instance, the β-lactamase inhibitor, clavulanic acid, is co-administered with β-lactam antibiotics, in particular penicillins and cephalosporins, to circumvent pathogen resistances against those. Apart from synergy, composite biological response can be caused by functional equivalents, potentially as a result of parallel evolution of defense against pathogens. Such cases are well documented in small molecule chemistry where, e.g., regioisomeric chlorogenic acids share the same activity such as inhibition of viral neuraminidase (Karar et al., 2016). As part of drug discovery, alternative compound compilations or structurally related derivatives are analyzed regarding bioequivalence, interchangeability, and drug substitution. For exploring the mechanism of combined effects, mathematical frameworks had proved to be successful, such as the ad-hoc reference models Loewe additivity (Loewe, 1953; Chou and Talalay, 1977, 1984; Berenbaum, 1989), Bliss independence (Bliss, 1956; Greco et al., 1995) and further Boolean modeling approaches (Vaidya et al., 2008; Flobak et al., 2015). In line with this, here both composite responses, synergy and redundancy of combinations of two LC-MS profiles, were implemented using the Boolean operators AND and OR, respectively, to enable the combined categorical correlation analysis.

**Figure 1:**
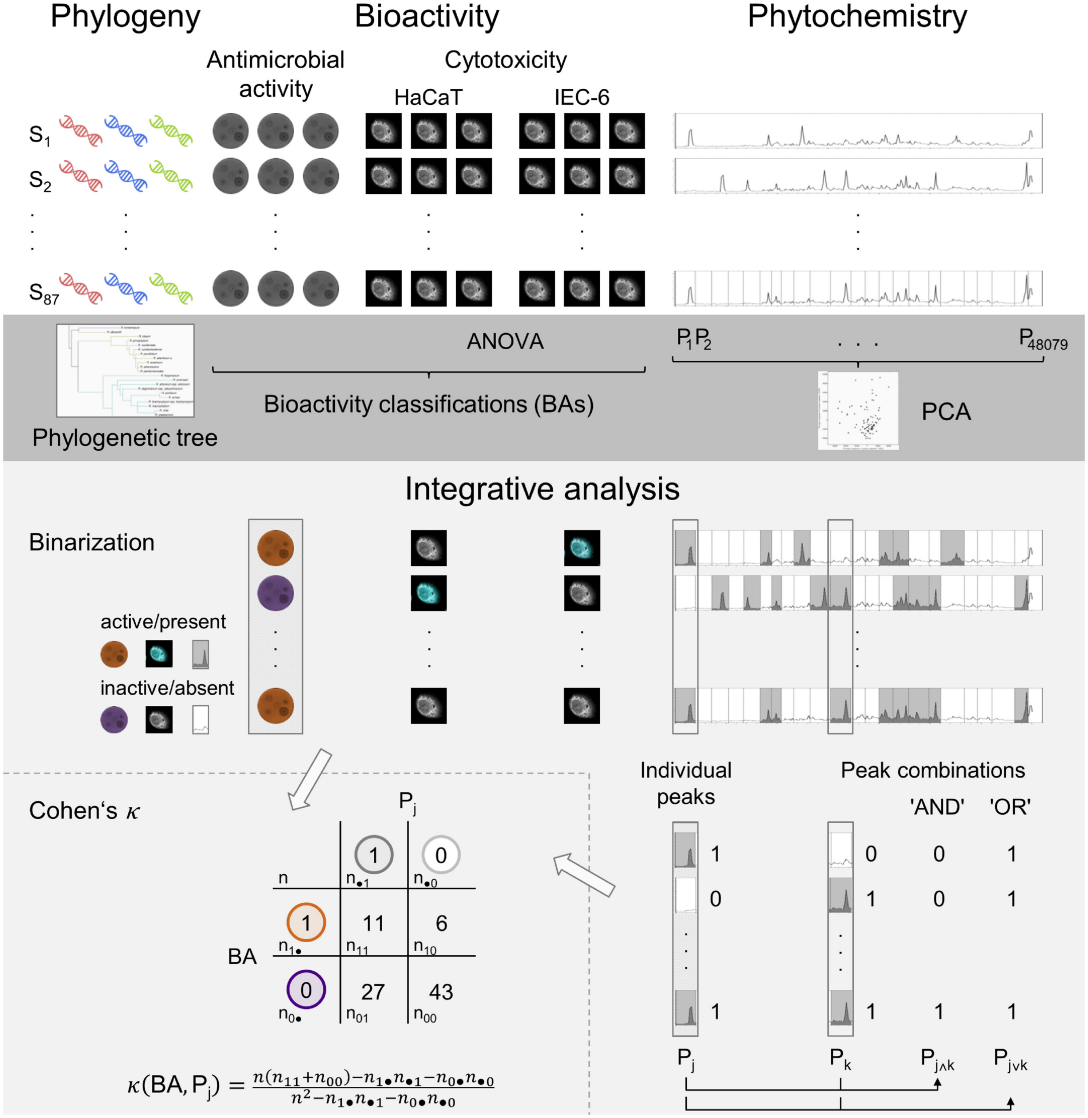
**Schematic overview of the experimental approaches (white background) and statistical analyses (gray backgrounds) in the presented study**. The dark gray region depicts the individual analyses while the brighter gray part illustrates the integrative analysis. Incorporating the individual results and based on the binarization of the processed data, bioactivity and phytochemistry are aligned in the correlation analysis, Cohen's κ.

The diversity of the genus *Rhododendron* qualifies for such a comprehensive analysis and, moreover, allows for examining the still highly discussed question at which scale phylogeny co-determines the bioactive phytochemical composition (Hegnauer, 1986; Wink, 2003; Rønsted et al., 2012). Based on the, to our knowledge, largest compilation of woody plant species, here *Rhododendron*, relations of phytochemical profile, phylogeny and bioactivities were surveyed and putative bioactive compounds were inferred (Figure 1). The herein used 87 *Rhododendron* species have been chosen from the five main subgenera of the genus: *Rhododendron* (excluding tropical vireyas), *Hymenanthes, Tsutsusi, Pentanthera*, and *Azaleastrum*, 10 of 11 sections, and 34 of 62 sub-sections in order to cover a broad taxonomic spectrum for this plant genus.

## Materials and methods

### Plant material and extraction procedure

Fresh leaf material of 87 reliably identified *Rhododendron* species were collected from plants grown in the Rhododendron-Park Bremen (www.rhododendronparkbremen.de) from January 2012 to December 2013. The identities of all plant species have been verified according to the German Genebank *Rhododendron* Database provided by the Bundessortenamt (www.bundessortenamt.de/rhodo; Dataset S1) and vouchers are deposited at the herbarium of the Botanical Garden at the University of Oldenburg (OLD).

For bioactivity and phytochemical analyses, leaf material was immersed in liquid nitrogen and grinded to powder. Crude extracts were prepared by re-suspending 2 g (fresh weight) of leaf powder in 10 ml 80% methanol (HPLC-grade, in freshly purified Millipore water) for 24 h at 4°C. Non-dissolved leaf residues were removed by centrifugation (3,220 × g, 30 min, 4°C).

### Phylogenetic analysis including DNA extraction, amplification, and sequencing

Total DNA has been extracted using the innuPREP Plant DNA kit (Analytik Jena, Jena, Germany). Two plastid gene regions *trn*K (*mat*K and 3′ end *trn*K) and *trn*L-F, and the nuclear ribosomal Internal Transcribed Spacer (ITS), were amplified by polymerase chain reaction (Table S9). Amplifications were performed using a TProfessional Standard Thermocycler (Biometra, Goettingen, Germany) or a Mastercycler^©^ gradient (Eppendorf, Hamburg, Germany). Sequencing reactions of PCR products were performed by GATC Biotech (Konstanz, Germany) with an ABI 3730xl (PE Applied Biosystem) automated sequencers. Nucleotide sequences were aligned with Geneious Pro v5.4.6 (Kearse et al., 2012). Contigs were aligned using MAFFT algorithm (Katoh and Standley, 2013) and manually edited.

The combined data sets were analyzed using Maximum Likelihood (ML) and Bayesian inference (BI). ML analyses were carried out using RAxML v7.9.5 (Stamatakis, 2006) where the best-fitting model (GTR with gamma correction) was selected by Akaike Information Criterion in jModelTest v3.7 (Posada, 2008). Non-parametric bootstrap analyses (1,000 replicates) were conducted to determine node support. BI analyses were performed in MrBayes 3.2.2 (Ronquist and Huelsenbeck, 2003) using the best-fit evolutionary model from ML analysis. Each analysis consisted of two independent runs, each with four Markov chains, proceeded for 10 million generations and sampled every 1,000th generation with the first 25% of the trees considered as burnin. Chain convergence and estimated sample sizes were confirmed to be sufficiently high in Tracer v1.5 (Rambaut and Drummond, 2009). For comparison posterior probabilities were reported on a percent scale (posterior probability × 100). Phylogenetic clustering of bioactivity against *B. subtilis* and cytotoxicity data (both cell lines) was tested via D-statistics (*p*-value testing whether D is significantly different from one) (Fritz and Purvis, 2010) using the R package caper (Orme et al., 2011).

### Antimicrobial susceptibility analysis

Antimicrobial activity screenings were conducted by agar diffusion method (Nathan et al., 1978) as described by Rezk et al. (2015b). Briefly, Lysogeny Broth (LB) agar plates were inoculated with 200 μl of the tester organism, *B. subtilis* strain 168, (1 × 10^7^ CFU/ml) by evenly spreading the cell suspensions over the agar surface. Wells with diameters of 5 mm were bored into the agar plates. Subsequently, 50 μl of the plant crude extracts were filled into each well. The plates were incubated overnight at 28°C. Inhibition of microbial growth was determined by measuring the radius of the inhibition zone. As negative solvent control, 80% methanol was used. All experiments were performed in triplicates and the results are presented as mean values. For the activity classification a radius of 0.6 cm has been chosen as threshold value based on our previous studies (Rezk et al., 2015a,b).

### Cytotoxic and proliferation analysis

The effects of *Rhododendron* leaf extracts on viability and proliferative activity of mammalian cells were quantified using the 3-(4,5-dimethylthiazol-2-yl)-2,5-diphenyltetrazolium bromide assay (MTT; Carl Roth, Karlsruhe, Germany). A detailed description and a thorough discussion on the necessity to combine it with other assays for a precise detection is provided elsewhere (Uzunova et al., 2010; Rehders et al., 2011; Rezk et al., 2015a; Karar et al., 2016). Here, two mammalian lines, normal rat small intestine epithelial cell line IEC-6 (Thomas and Oates, 2002; Mayer et al., 2009) and human keratinocyte cell line HaCaT (Büth et al., 2007; Rehders et al., 2011) were used as described by Rezk et al. (2015a) in order to mimic intact tissue. Thus, a value exceeding the MTT conversion of the solvent control (DMSO) would indicate induction of hyper-proliferation, while a value below the control serves as an indication of cytotoxic effects that can be explained by both, compound-induced cell death or reduced proliferation rates.

Briefly, cells were seeded in 96-well plates (Greiner bio-one, Essen, Germany) at a density of 1 × 10^4^ cells/well until reaching the desired density (HaCaT 90–100%, IEC-6 70%). Upon incubation with culture medium containing 50 μg/ml *Rhododendron* leaf extracts and an 0.05% (v/v) DMSO solvent control for 24 h, cells were incubated with fresh culture medium containing MTT at a final concentration of 0.5 mg/ml for another 4 h. The supernatants were removed, and 100 μl of 100% DMSO was added to each well. Absorbance was read at 595 nm using a microplate reader (Tecan, Männedorf, Switzerland). As negative control, cells incubated with culture medium containing DMSO, only, at a final concentration of 0.5% (v/v) were used. Percentages of cell viability were calculated from triplicates according to (Equation 1),

(1) $$\begin{array}{r} \text{cell viability}\left[\%\right]=\frac{{\text{A}}_{\text{treated cells}}}{{\text{A}}_{\text{control cells}}}\text{.} \end{array}$$

For the cytotoxicity classification, a one-way ANOVA was performed, including multiple testing using the Benjamini-Hochberg procedure at false discovery rate level α = 0.05 (Benjamini and Hochberg, 1995).

### Phytochemical analysis

Phytochemical profiles were determined on an Agilent 1200 HPLC system using a 5 μm Diphenyl column (inner diameter 250 × 3 mm; Varian, Darmstadt, Germany), equipped with a binary pump, an auto-sampler with a 100 μl loop, and a UV-Vis detector with a light-pipe flow cell. The binary solvent system consisted of water/formic acid (1,000:0.05 v/v, Solvent A) and methanol (Solvent B). A linear gradient from 10 to 80% B in 70 min with an increase of 10% every 10 min was employed at a constant flow rate of 0.5 ml/min. The HPLC system was connected to a microTOF mass spectrometer (Bruker Daltonics, Bremen, Germany) coupled to an electrospray ionization source. The TOF analyzer was calibrated with a 0.1 M sodium formate solution, injected prior to each chromatographic run, using the enhanced quadratic mode. Tandem mass spectra were obtained under the same conditions as above using a quadrupole ion trap mass spectrometer with auto MS^*n*^ mode (Bruker HCT ultra, Bruker Daltonics, Bremen) coupled to an electrospray source. All mass spectra were acquired in the negative ion mode.

Analyses of HPLC-TOF-MS raw data, in terms of peak detection, peak matching, and retention time alignment, were performed using the R packages XCMS (Smith et al., 2006; Tautenhahn et al., 2008) and MAIT (Fernandez-Albert et al., 2014). For determination of the involved parameter the toolbox IPO has been used (Libiseller et al., 2015). In the course of peak annotation, the intensity threshold was set to 10^3^ and^13^C isotope peaks were removed using the Bioconductor package CAMERA (Kuhl et al., 2012). In addition, all peaks common to all samples were discarded from the sample data set prior further analysis.

Based on the overall 48,079 peaks generated by TOF-MS, a principal component analysis (PCA) was performed using centering and Pareto scaling (van den Berg et al., 2006). For highlighting the data separation, a two-standard deviational ellipse was added for each group based on the mean, standard deviation, and the covariance of the corresponding data.

Furthermore, the high resolution TOF-MS data along with the complementary ion trap measurements were analyzed via Bruker Data Analysis software. The resulting total and extracted ion chromatograms and tandem mass spectra were used to predict molecular formula and detailed structural information for the assignment of compounds and their derivatives recorded in the in-house library.

For the activity-guided fractionation of *R. ambiguum* b leaf extract, a pipeline was set up of preparative HPLC for separation, and LC-MS^*n*^, LC-TOF-MS, and NMR for further analysis. The prep-HPLC system consist of a 5 μm Diphenyl column with an 8 mm guard column (Macherey-Nagel, Germany), equipped with two binary pumps, a six port manual injector with 500 μl loop, and a UV-Vis detector (Varian Pro Star, USA). The binary solvent system consisted of water/formic acid (1,000:0.05 v/v, Solvent A) and acetonitrile (Solvent B). A gradient from 10 to 90% B was employed with an increase to 70% after 10 min and 10% every additional 10 min at a constant flow rate of 4 ml/min. The fractions tested positively for antimicrobial activity against *B. subtilis* (inhibition zone radius of ≥0.6 cm) were subjected to LC-MS^*n*^ and LC-TOF-MS analysis (conditions see above). The two final observed isomers of *m/z* 333 were analyzed using different NMR, i.e., ^1^H, ^13^C, ^13^C DEPT, COSY, NOESY, HSQC, and HMBC.

### Bioactivity classification for identified secondary metabolites

For analysis of the identified compounds based on our in-house compound library, the group of tested *Rhododendron* species was divided into active and inactive species regarding each of the three bioactivity thresholds, i.e., 0.6 cm antimicrobial inhibition radius and false discovery rate level α = 0.05 for significance of both cytotoxicity measures. For each identified compound the ensuing classification was compared by means of a two-tailed unpaired T-test and visually through their quartiles using boxplots.

### Categorical correlation analysis

For each *Rhododendron* species up to 20,000 LC-MS peaks were recorded, together with antimicrobial activity and cytotoxicity measurements. In order to reduce the complexity of the data set, all measured variables were binarized representing presence or absence of the peaks or one of the bioactivity indicators. In this respect, an intensity threshold of 10^4^ for the detected LC-MS peaks, an 0.6 cm antimicrobial inhibition radius and a false discovery rate level α = 0.05 for both cytotoxicity analyses were used. By means of Cohen's Kappa (Cohen, 1960; McHugh, 2012), the agreement between two raters, the phytochemical and the respective bioactivity measures, can be evaluated (see Figure 1).

To assess compliance to both, antimicrobial activity against *B. subtilis* and non-cytotoxicity toward the mammalian cell lines, a combined quality measure was introduced, the linear combination of the individual correlation coefficients, according to (Equation 2),

(2) $$\begin{array}{r} {\bar{\kappa }}_{\text{C}}=\kappa_{\text{AM}}-\left(\kappa_{\text{HaCaT}}+\kappa_{\text{IEC-6}}\right)\text{.} \end{array}$$

An LC-MS peak was admitted to be most-predictive for an antimicrobial active but non-cytotoxic compound if also the individual categorical correlation analysis regarding antimicrobial activity against *B. subtilis* showed strong correlation (${\bar{\kappa }}_{\text{C}}$ ≥ 0.68 ∧ κ_AM_ ≥ 0.68).

For the qualitative exploration of additive and synergistic effects, and functional redundancy, binarized LC-MS peak profiles were linked by the Boolean operators AND and OR, respectively. The resulting LC-MS profiles were applied to categorical correlation analysis in the same manner as the individual peak profiles.

## Results

At the core of this study is the large compilation of leaves from *Rhododendron* species, all cultivated and harvested in the Rhododendron-Park Bremen. The 87 species comprise species from the two large subgenera *Rhododendron* (38) and *Hymenanthes* (31), and another three of the remaining six subgenera, namely *Pentanthera* (12), *Tsutsusi* (4), and *Azaleastrum* (2). To obtain a representative collection, *Rhododendron* species were selected according to the size of the five subgenera, i.e., the largest subgenus, *Rhododendron*, is represented by the highest number of species.

### Phylogeny of *Rhododendron*

**Figure 2:**
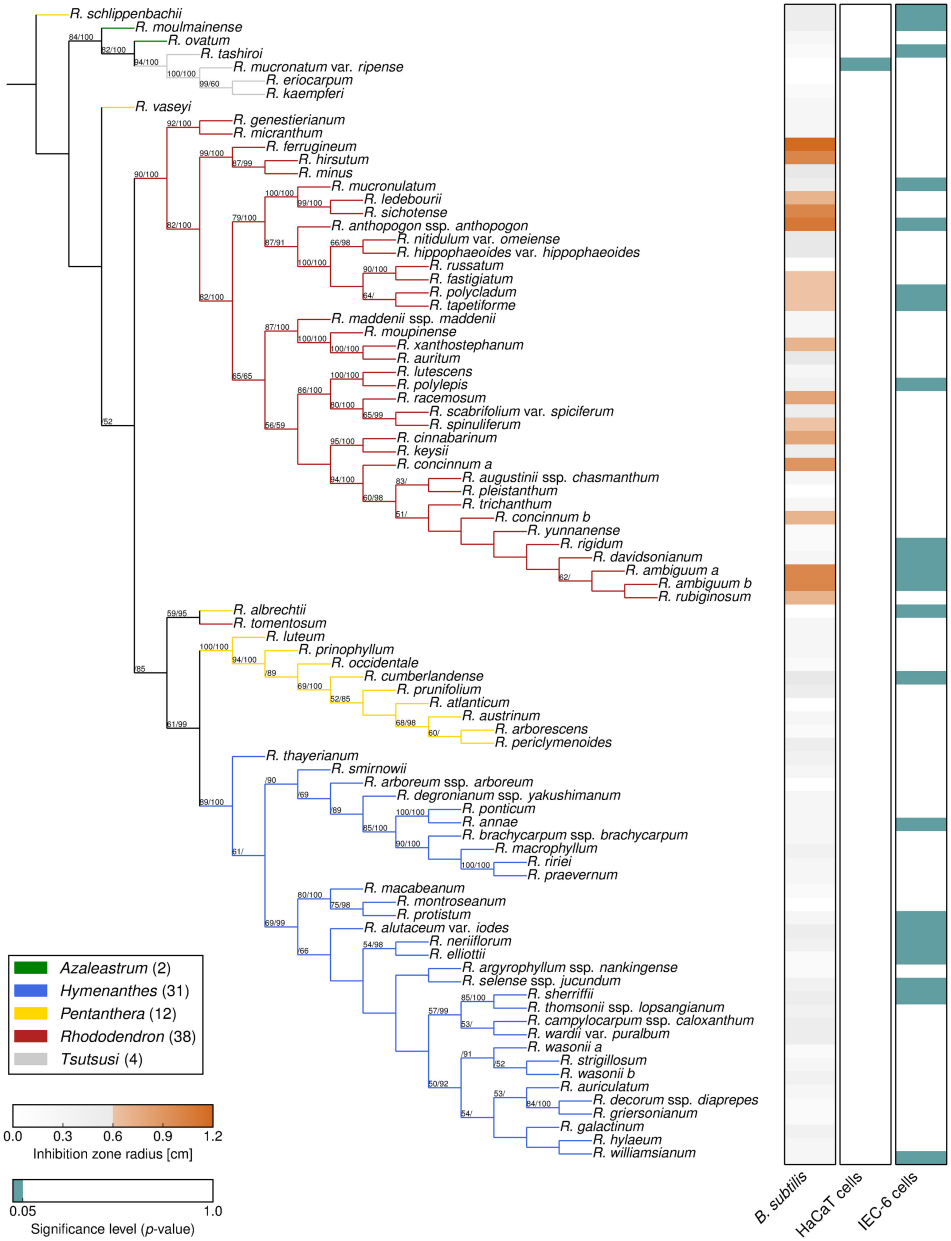
**Phylogeny and bioactivities of *Rhododendron***. The phylogenetic tree of the 87 *Rhododendron* species is based on three genetic markers, *trn*K, *trn*L-F, and ITS. The support values from likelihood bootstrap analysis (before slash) and Bayesian inference (after slash) are denoted at the respective branches. The three panels show the antimicrobial activity against *B. subtilis* **(left)**, and the cytotoxicity toward HaCaT cells **(middle)**, and IEC-6 cells **(right)** with respect to the given threshold and significance level, respectively.

To assess the exceptional diversity of the genus *Rhododendron*, a combined phylogeny based on three DNA regions, *trn*K, *trn*L-F, and ITS, was constructed. Thereby, Bayesian inference and Maximum Likelihood analyses resulted in phylogenetic trees of the genus *Rhododendron* with the same topology (Figure 2).

The two largest subgenera, *Hymenanthes* and *Rhododendron* (excluding *Rhododendron tomentosum*) and the subgenus *Tsutsusi* form monophyletic groups. In contrast, the subgenera *Pentanthera* and *Azaleastrum* are polyphyletic. These findings are in line with previous studies on the phylogeny of the genus *Rhododendron* and the family *Ericaceae* using different markers (Goetsch et al., 2005; Schwery et al., 2014). The results support that our sampling is representative for the genus and allows for a conclusive survey of the bioactivities performed in this study. Moreover, the presented compilation allows addressing herein the vividly discussed hypothesis stating that phylogeny predicts phytochemical diversity (Hegnauer, 1986; Wink, 2003; Rønsted et al., 2012).

### Antimicrobial effects of *Rhododendron*

As a benchmark for *Rhododendron*'s antimicrobial activity, comparative agar diffusion assays testing against *B. subtilis* had been performed indicating a phylogenetic clustering (D = 0.31, *p* < 0.001) of antimicrobial activities exclusively in leaf extracts of the subgenus *Rhododendron* (Figure 2, Dataset S1). Particularly, leaf extracts of 17 species exhibited distinct, the 0.6-cm-inhibition-radius threshold-exceeding antibacterial activities, suggesting that these extracts contain at least one, or even multiple antimicrobial compounds.

This activity classification is in agreement with previous studies (Popescu and Kopp, 2013; Rezk et al., 2015a,b) corroborating the notion of *Rhododendron* species exerting antimicrobial effects against Gram-positive bacteria. However, crude extracts had been tested thus far, and therefore, such a qualitative assessment and, additionally, thresholding must be considered an approximation. Both need to be confirmed by dose-response curves determining minimum inhibitory concentrations (MIC) as soon as purified substances are available to obtain a stricter classification.

### Cytotoxicity of *Rhododendron*

For the application of *Rhododendron* leaf extracts as part of a medicinal formulation, or as food additives, toxic impact of antimicrobial extracts or compounds on mammalian cells are to be avoided. Analyses of metabolic activity and proliferation rates by MTT-assay indicated that the cytotoxic effects vary strongly for the two different cell lines investigated (Figure 2, Dataset S1). Intestine epithelial (IEC-6) cells were affected much stronger by incubation with *Rhododendron* leaf extracts than epidermal keratinocytes (HaCaT; Figure 2, compare panels on the right, green depicts cytotoxicity). Cytotoxic effects toward IEC-6 cells were exerted by leaf extracts prepared from species widely spread across the genus *Rhododendron*, and there was no phylogenetic clustering (D = 0.66, *p* = 0.66) such as observed for antimicrobial activities (Figure 2, cf. right panels, green vs. orange). Because the extract of a single *Rhododendron* species, only, showed significant cytotoxic effects toward HaCaT cells, no conclusion can be drawn regarding keratinocyte-affecting compounds produced in the genus *Rhododendron*. At the concentrations applied, the leaf extracts of 12 of the 17 *Rhododendron* species denoted to be antimicrobial against *B. subtilis* can be considered non-cytotoxic to any of the two mammalian cell lines investigated in this study. However, to attest absence of cytotoxic effects of leaf extracts consisting of several different compounds, further and more detailed cell biological assessments need to be performed as previously emphasized in a study that used a smaller collection of *Rhododendron* species (Rezk et al., 2015a).

For both, assessments of antimicrobial activity as well as exclusion of gross cytotoxicity, further analyses are required, such as determination of MIC in dose-response curves upon incubation with specific chemical compounds, in order to confirm bioactivity profiles of the different species analyzed. Hence, in the further course of this study, detailed phytochemical profiling was approached with the aim to identify the chemical nature of bioactive compounds, i.e., acting as inhibitors of the growth of Gram-positive bacteria, while not exerting cytotoxic effects onto keratinocytes and/or intestine epithelial cells.

### *Rhododendron*'s phytochemical fingerprint

Within the framework of the fingerprint more than 48,000 liquid chromatography coupled to mass spectrometry (LC-MS) peaks across the 87 *Rhododendron* species could be detected, each one uniquely defined by a mass-to-charge ratio (*m/z*) and a retention time. In selecting these 48,000 peaks, signals common to all extracts, comprising both solvent impurities and primary metabolites as well as isotope peaks have been removed from the data set. However, the number of detected peaks does not reflect the actual number of chemical compounds since the data set still includes both, fragment and adduct ions. It is important to keep this in mind when performing statistical analyses, which are using every individual peak signal. Detailed structure assignment on polyphenolic compounds with flavonoids (proanthocyanidins, hydroxycinnamates and hydroxybenzoate derivatives) allowed identification of around 300 secondary metabolites across all *Rhododendron* species. For approximately half of these, a definite structure could be assigned based on authentic reference compounds. For the remaining half a tentative structure could be suggested leaving open details of regio- and stereochemistry. These results indicated that the vast majority of peaks represented unknown or non-identifiable compounds.

#### Principal component analysis

As a first statistical assessment of the data, clusters and dependences in the phytochemical screening data have been inferred by means of principal component analysis (PCA) with the aim to reveal differences among species and to determine the most-predictive attributes. As such potential attributes the classifications with respect to antimicrobial activity against *B. subtilis*, cytotoxicity toward both mammalian cell lines, as well as the phylogeny were taken into account.

**Figure 3:**
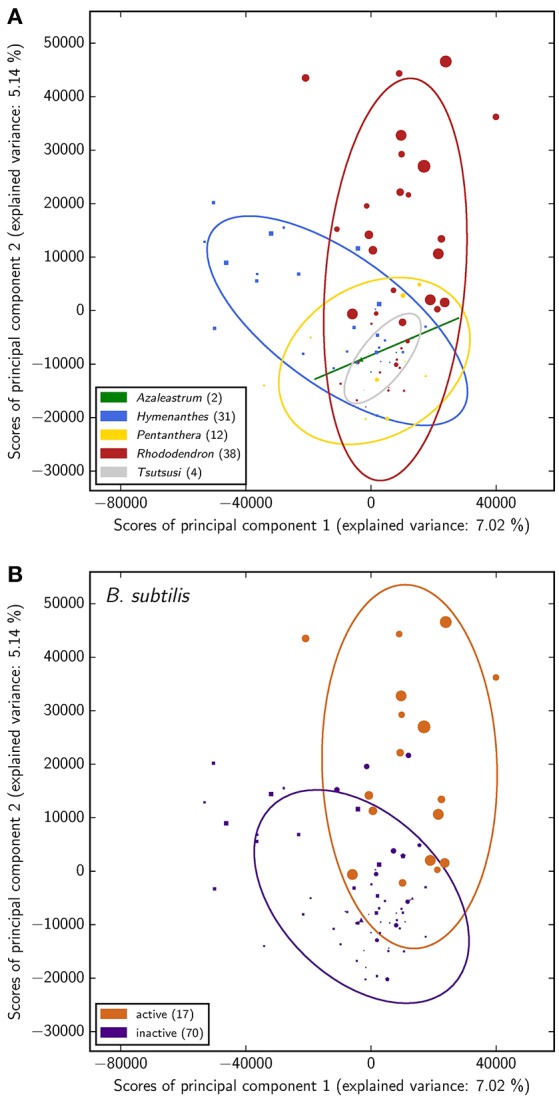
**Principal component analysis of the phytochemical data for all 87 *Rhododendron* species**. The scores of the principal components corresponding to the species are colored with respect to the corresponding subgenera **(A)** and antimicrobial activity classification **(B)**, respectively. In addition, the item shape highlights the subgenus of each species (△:*Azaleastrum*, ◻:*Hymenanthes*, 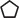:*Pentanthera*, ◯:*Rhododendron*, ▽:*Tsutsusi*) and the item size illustrates the antimicrobial activity of the respective species (the larger the more active).

The PCA suggests rather a connection of *Rhododendron*'s phytochemistry to antimicrobial activity against *B. subtilis* than to its phylogeny while no conclusions can be drawn about a relation of phytochemical profiles and cytotoxicity exerted onto either of the two mammalian cell types (Figure 3 and Figure S1). This is reflected in the groups' separation and illustrated by the corresponding coloring in the score plot of the PCA. However, the predictive value of the PCA is limited in the sense that the explained variance proportion of the first two principal components is no more than 12%. Accordingly, examining the corresponding loading plot (Figure S2) does not provide reliable insights on most-contributing LC-MS peaks. Moreover, the fact that the here measured antimicrobial activity occurs exclusively in the subgenus *Rhododendron* points to a certain dependency of both separations, according to antimicrobial activity against *B. subtilis* and phylogeny. This dependency clearly affects the inferences on the respective relation of antimicrobial activity and phylogeny to *Rhododendron*'s phytochemical fingerprint.

Besides phylogeny and defense mechanisms against pests and pathogens, other factors such as environment may influence the phytochemical profile. While different species may react differently to the same climatic parameters and pathogen-attack may be heterogeneous across species and habitat, the weak signal of the phylogeny suggests little differentiation in phytochemistry across the genus. Further analysis with respect to the chemical diversity (i.e., number of substances for detected peaks and identified compounds) of each subgenus also did not show any significant connection of phylogeny and phytochemistry (Figure S3). Considering these findings, the hypothesis that phylogeny determines the phytochemical diversity cannot be corroborated by our analysis.

As mentioned before, the number of peaks in the LC-MS analysis usually exceeds the number of substances present in the crude extracts. Since the herein conducted PCA is based on the individual peak signal, our predictions might be biased towards substances, which are represented by more than one peak. Together with the fact that the predictive power of the PCA is limited, a potential relation of *Rhododendron*'s phytochemistry to its antimicrobial activity against *B. subtilis* needs further validation using customized statistical analysis such as the following categorical correlation analysis.

### Relation of phytochemistry and bioactivity of *Rhododendron*

In line with the PCA, the relation of phytochemistry and antimicrobial activity, particurlarly against *B. subtilis*, was further surveyed. In addition, the phytochemical data was examined with respect to cytotoxicity toward the two mammalian cell lines to reveal potential relations. More precisely, the relation of the respective bioactivity classifications and two aspects of the phytochemical profiles were explored across all *Rhododendron* species: (1) the identified secondary metabolites, and (2) the detected LC-MS peaks representing so far unidentified compounds.

#### Comparative analysis of identified secondary metabolites

Among the 87 *Rhododendron* species, 292 different polyphenols could be identified based on in-house compound libraries (Dataset S1). In total, these identified compounds depict only a very small portion (0.6%) of the detected peaks. Nonetheless, insights about the bioactivity of these compounds allows for comparison to previously published findings on *Rhododendron* and other plants (Popescu and Kopp, 2013).

**Figure 4:**
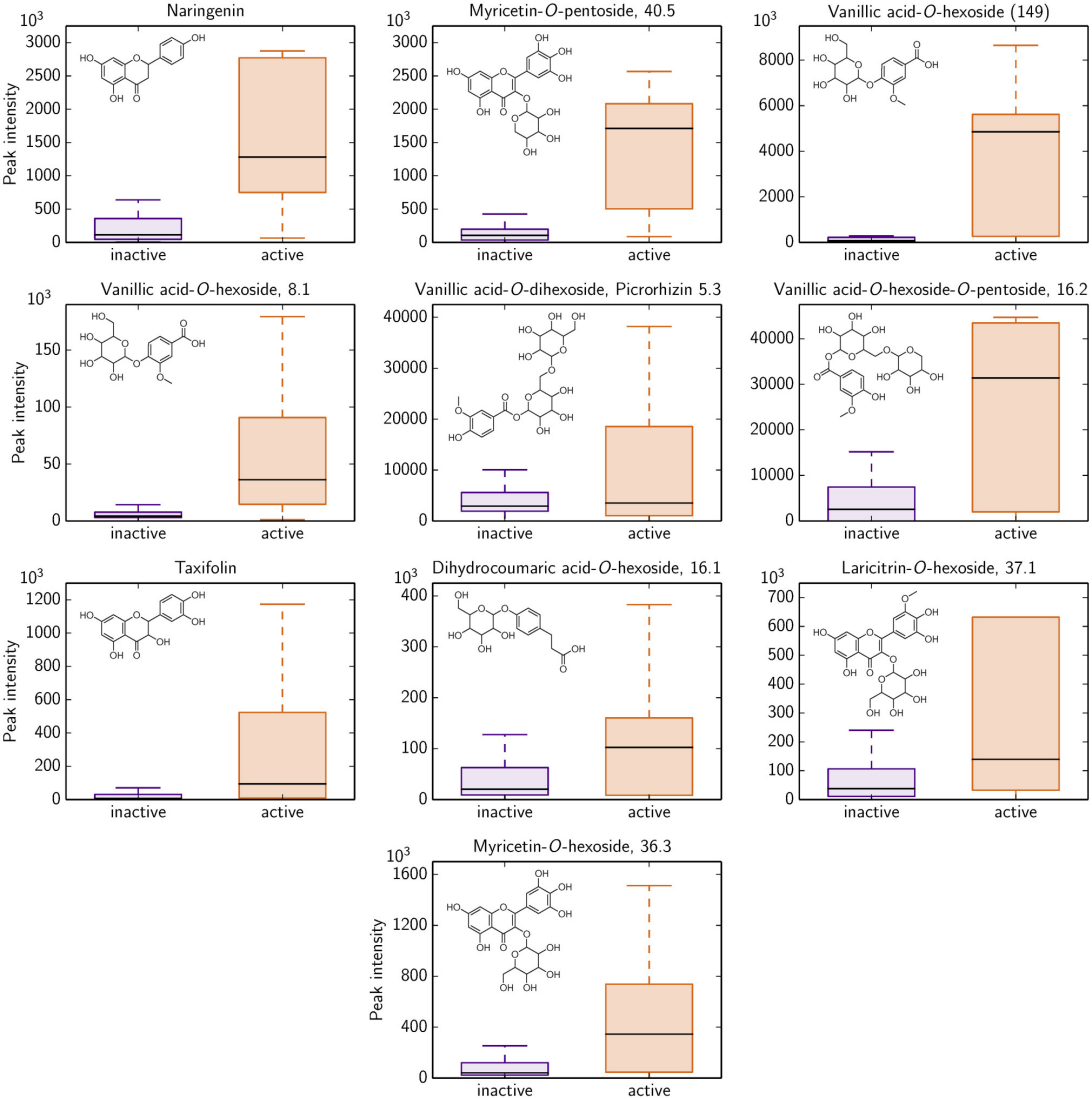
**Identified polyphenolics showing significant differences in LC-MS intensity with respect to antimicrobial activity classification of all 87 *Rhododendron* species (remaining 282 identified compounds are provided in Figure S4)**. 17 species are denoted as antimicrobial active (orange), i.e., the radius of the agar diffusion assay is ≥0.6 cm, and, thus, 70 species are characterized as antimicrobial inactive (violet).

Based on the LC-MS screening intensities, 10 of these 292 compounds show a significant difference for antimicrobial active and inactive *Rhododendron* species. All 10 polyphenols could potentially serve as antimicrobial activity against *B. subtilis* indicators since their occurrence is much more pronounced in those *Rhododendron* species declared as active ones (Figure 4). Besides naringenin and taxifolin, these 10 polyphenolics involve glycoside derivates of dihydro-*p*-coumaric acid, laricitrin, myricetin, and vanillic acid.

For cytotoxicity toward mammalian cell lines, no such indicator compound could be determined. No compound shows significant differences according to the respective cytotoxicity classification of *Rhododendron* species (Figures S5, S6). This reinforces the PCA prediction (see above) that there is no noticeable relation of phytochemical profile and cytotoxicity exerted toward the mammalian cell lines investigated in this study.

Keeping in mind that the identified compounds cover only a small proportion (0.6% of the detected LC-MS peaks) of the chemical space present in *Rhododendron*, the proposed compounds might be unsatisfactorily predicting the antimicrobial activity, particurlarly against *B. subtilis*. Conceivably, there might exist much stronger indicators for the bioactivity among the so far unidentified compounds originated by the remaining LC-MS peaks.

#### Categorical correlation analysis of the detected LC-MS peaks

To explore further potentially bioactive compounds, the more than 48,000 detected LC-MS peaks (combination of high-resolution *m/z* values and associated distinct retention times) were correlated with the respective bioactivity classifications. For this purpose, the LC-MS profiles were binarized, illustrating the presence and absence of the respective peak for each *Rhododendron* species. As expected, there exists not a single LC-MS peak that matches perfectly to the antimicrobial activity classification against *B. subtilis* or the one regarding the cytotoxicity toward both mammalian cell lines.

**Figure 5:**
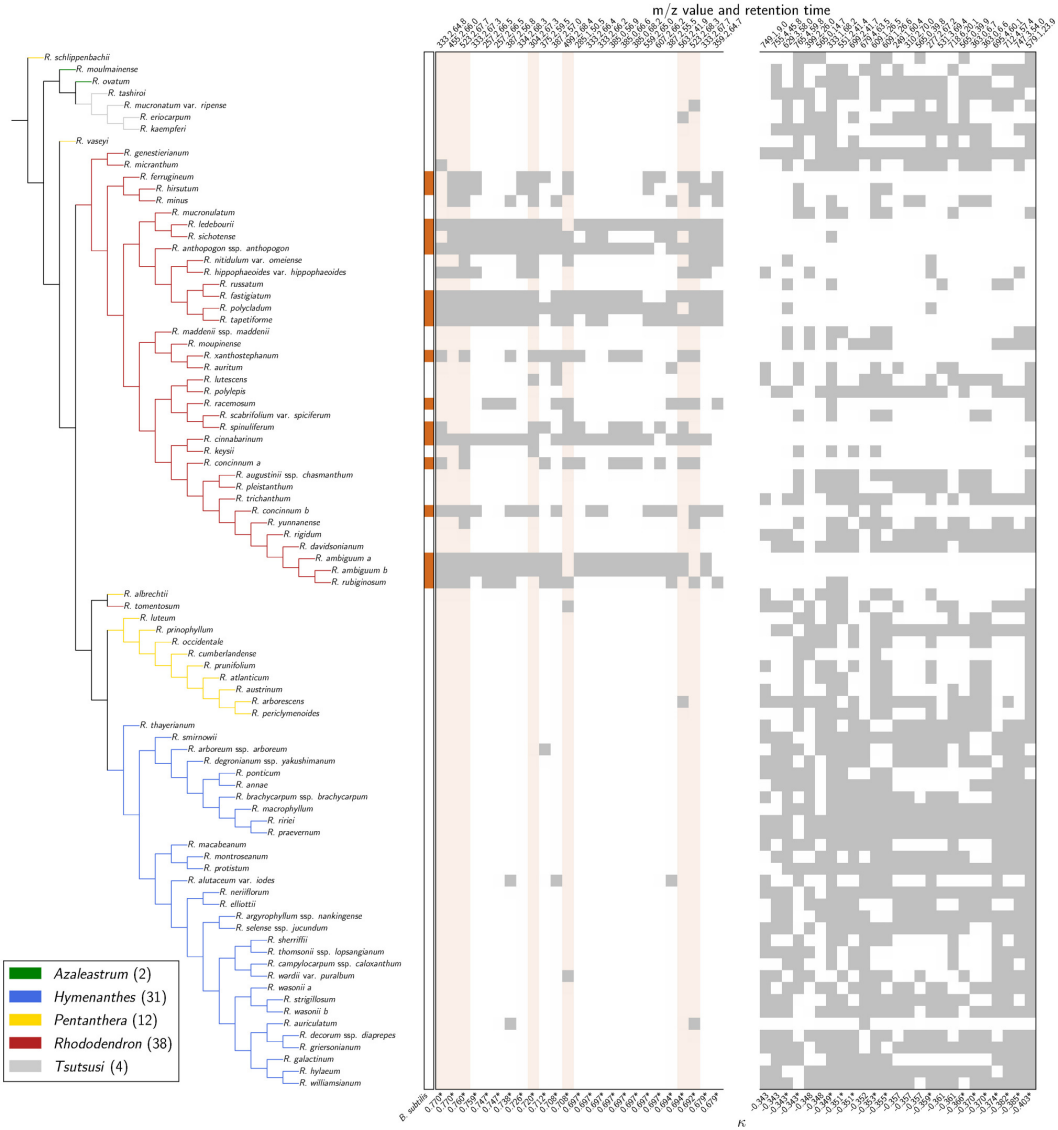
**The 25 most- and least-predictive LC-MS peaks with respect to Cohen's kappa correlation for antimicrobial activity across all 87 *Rhododendron* species mapped against their phylogeny**. A sample is denoted as antimicrobial active (orange) if the radius of the agar diffusion assay is ≥0.6 cm. A compound, defined by an *m/z* ratio and retention time tuple, is denoted as present in a sample (gray) if its intensity is ≥10,000. The ^*^ emphasizes significant correlations with respect to multiple testing correction by Benjamini-Hochberg (α = 0.05). The seven most-predictive peaks regarding antimicrobial activity and non-cytotoxicity at once are highlighted in bright orange.

However, 23 of the 48,079 peaks show strong correlations with respect to antimicrobial activity against *B. subtilis* (≥0.68; Figure 5). The categories “strong,” “moderate,” and “low” correlations are phenomenological, as the exact boundaries contain some arbitrariness (Taylor, 1990). These most-predictive peaks have predominantly high retention times and low *m/z*, meaning that they are likely to be highly hydrophobic compounds (Tables S1, S2 and Figure S7). In contrast, the 10 polyphenolics suggested above as potential indicator for antimicrobial activity show a higher degree of hydrophilicity as judged by their shorter average retention times on a reversed phase packing. Moreover, none of the previously suggested polyphenolics is among the most-predictive peaks regarding antimicrobial activity against *B. subtilis*. Particularly, the first of these matches is vanillic acid-*O*-hexoside, 8.1 on rank 996 out of 48,079 according to the correlation coefficient, and thus among the top 2.1% of detected peaks.

Regarding the cytotoxicity toward IEC-6 cells, there exists not a single peak showing strong correlation and only 13 peaks with at least a moderate correlation signal (0.35 to 0.67; Figure S8). In contrast to the most-predictive antimicrobial active peaks, the well-predicting peaks for cytotoxicity toward the IEC-6 cells are widely distributed across *m/z* ratio and retention time range (Table S1). In case of cytotoxicity exerted toward HaCaT cells, the correlation coefficient as well as the distribution over *m/z* ratios and retention times are hardly informative since only a single species showed significant cytotoxic effects (Table S1 and Figure S9). Accordingly, the 26 peaks showing perfect correlation represented compounds occurring only in this particular species thus not allowing any conclusive thoughts.

Taken together, the categorical correlation analysis reinforces the strong relation of phytochemistry and antimicrobial activity against *B. subtilis* but also shows moderate relation of phytochemistry and cytotoxicity toward the intestinal cell line. In addition, for the 23 most-predictive antimicrobial active peaks the correlation signals with respect to cytotoxicity exerted toward both mammalian cell lines are only weak (Table S2). In order to reveal the most-predictive peaks for an antimicrobial active compound which can also afford certain safety for application to human cells, in a first attempt, a linear combination of the quality measures, i.e., correlation coefficients, was determined. This combined relationship coefficient was intended to give preference to correlation with antimicrobial activity classification against *B. subtilis* and an anti-correlation with the cytotoxicity toward the mammalian cell lines (see Section Materials and Methods, Equation 2). As a result, 7 out of 23 most-predictive peaks emerged with a strong signal regarding antimicrobial activity and non-cytotoxicity (Table 1, Table S3 and Figure S10). These seven peaks might represent a first assessment for an antimicrobial active compound from *Rhododendron* that can be considered non-cytotoxic to any of the two mammalian cell lines investigated in this study.

**Table 1:** **The seven most-predictive peaks for antimicrobial active but non-cytotoxic compounds across all 87 *Rhododendron* species**.

***m/z* ratio**	**rt (min)**	**${\bar{\kappa }}_{\text{C}}$**	**Rank_C_**	**Rank_AM_**	**Rank_HaCaT_**	**Rank_IEC-6_**
499.17	68.4	0.82	1	11	32,668	42,896
523.19	67.7	0.79	2	3	34,746	25,643
523.21	68.3	0.75	4	23	1,777	46,192
304.16	67.3	0.73	7	9	34,075	21,733
333.19	64.8	0.71	11	1	31,117	7,956
455.20	66.0	0.71	11	1	31,117	7,956
563.17	61.9	0.70	14	21	31,117	21,419

*The peaks are uniquely determined by m/z ratio and retention time (rt) and have attributed the combined relationship coefficient, ${\bar{\kappa }}_{C}$, and corresponding rank (C), as well as the individual ranks according to correlation coefficient for antimicrobial activity and the cytotoxicity toward HaCaT and IEC-6 cells*.

To experimentally confirm that any member of the above peak list possesses antibacterial activity, an activity-guided fractionation was carried out exemplarily on leaf extracts of *Rhododendron ambiguum* b, which was among the species with the highest antimicrobial activity against *B. subtilis*. Following the standard phytochemical procedure, a highly active fraction was obtained containing a mixture of two isomeric compounds with an *m/z* of 333 ([M-H] C_18_H_22_O_6_). All further separation attempts on the two isomeric compounds failed and due to signal overlap in 2D NMR spectra, no defined structure could be suggested. Tentatively, the two compounds are derivatives of cannabinoic acid with isomerism at the side chain terpene olefinic moiety. Most importantly, the joint pair of *m/z* and retention time for these compounds was predicted as best candidate for an antimicrobial active compound (Table 1, column 5), impressively demonstrating the value of the herein conducted statistical analysis, which could be experimentally confirmed by a ‘blind’ activity-guided screening.

#### Categorical correlation analysis of LC-MS peak combinations

**Figure 6:**
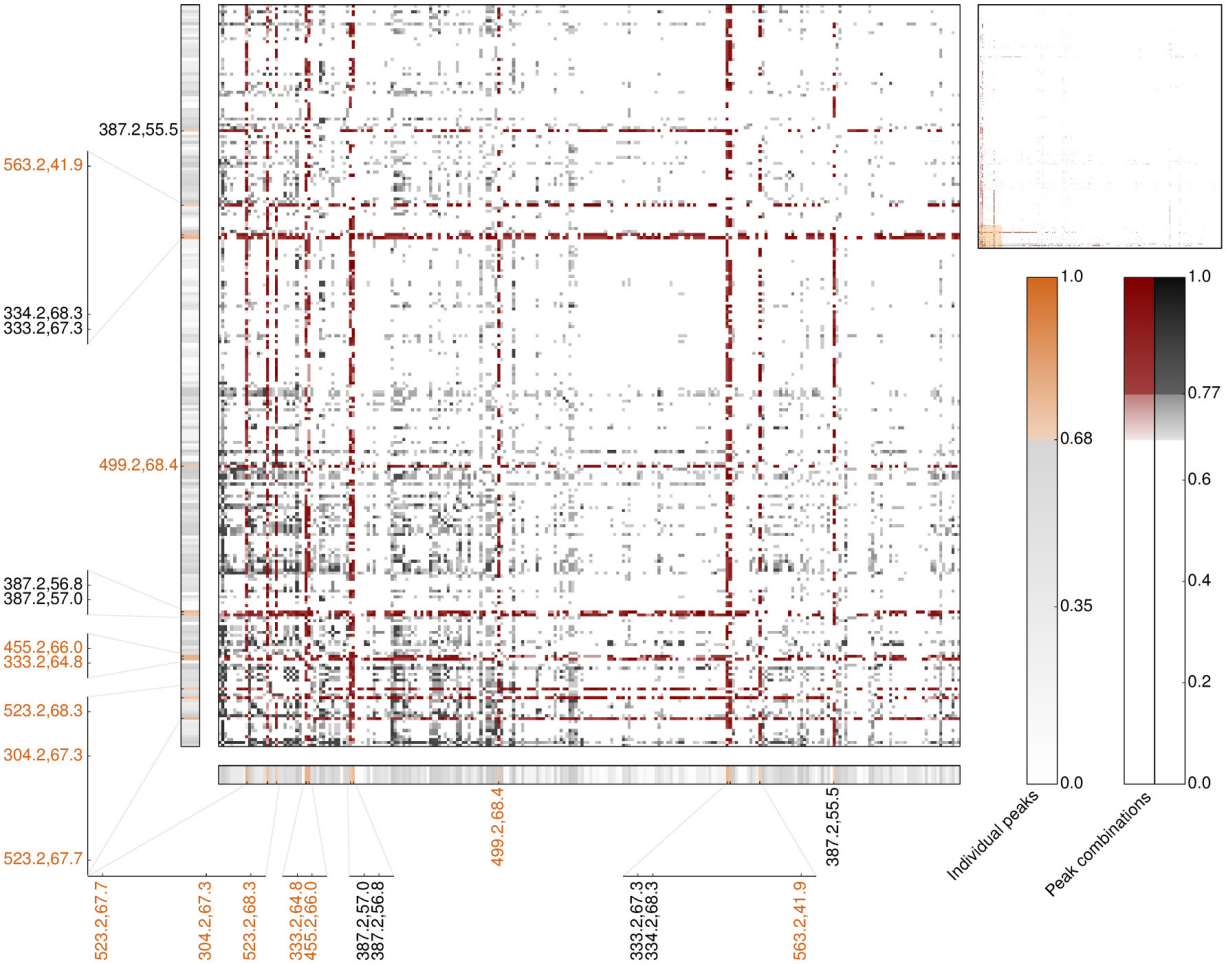
**Heatmap of the top 250 LC-MS peaks involved on the most-predictive additive peak combinations regarding Cohen's kappa correlation for antimicrobial activity across all 87 *Rhododendron* species (main panel)**. The upper right panel provides the overview of all 643 peaks included in the outperforming additive peak combinations, namely κ ≥ 0.77. The combinations highlighted in red involve at least one of the 23 most-predictive peaks regarding the individual peak analysis, κ ≥ 0.68. The corresponding individual peak correlation coefficients are depicted in the thinner horizontal and vertical panels. Orange labels emphasize the seven most-predictive individual peaks regarding antimicrobial activity and non-cytotoxicity.

In order to assess whether compound combinations might better explain intricate biological responses, such as bioactivity, categorical correlation analysis had been extended to allow for investigating each combination of two LC-MS profiles. Following established mathematical frameworks for additivity and redundancy, for this Boolean operators AND and OR had been applied, respectively. Compared to the individual peak analysis, the correlation signals of measured antimicrobial activity and LC-MS profiles are considerably more pronounced for both, AND and OR combinations (Figures S11, S12). While possible additive or synergistic combinations strongly point out the already identified individually high-predictive peaks, potential alternative interconnections show much stronger correlation coefficients compared to the individual peak analysis. 28,209 of the more than 1.1 billion additive peak combinations show a strong correlation signal (≥0.68) comprising 1,669 combinations outperforming the correlation of any individual peak (≥0.77). Interestingly, the majority of these outperforming combinations (>65%) comprise one of the 23 most-predictive individual peaks regarding antimicrobial activity against *B. subtilis* including all seven most-predictive peaks regarding antimicrobial activity and non-cytotoxicity (Figure 6 and Table S4). In particular, the experimentally validated peak with an *m/z* value of 333 is present in more than 13% of the outperforming peak combinations (225 of 1,669). Following this, additivity and/or synergy with respect to antimicrobial activity is, if indeed, very likely accomplished by a pair of peaks already detected in the individual peak analysis (Figure 6, lower left region). Our results furthermore indicate that the best-predictive peak pairs very likely comprise one of the most-predictive individual peaks (Figure 6, band-forming patterns). This in turn, reinforces the reliability of the 23 individual peaks assigned to operate antimicrobial active and, moreover, suggests that these pairs comprise one antimicrobial active peak against *B. subtilis* and one associate peak potentially having an accessory function.

In case of alternative effects, 684,428 of the more than 1.1 billion peak combinations showed strong correlations with respect to antimicrobial activity against *B. subtilis* (≥0.68) involving 105,761 combinations that outperform any individual peak correlation-wise. Approximately 31% of these outperforming peak combinations comprise at least one of the 23 most-predictive individual peaks regarding antimicrobial activity against *B. subtilis* (Table S5 and Figure S13). Even the experimentally validated peak with *m/z* 333 is present in these peak combinations depicting the most reliable candidates of peak pairs operating as functional equivalents or affording functional redundancy. However, only 3% of the outperforming peak combinations comprise this peak (3,790/105,761) and in only 17 cases another of the most-predictive individual peak participates. This demonstrates that the attention has mainly been devoted to peaks that have not shown up in the individual peak analysis. Interestingly, the correlation signals of the alternative peak combinations are more pronounced than the ones of the additive effects, i.e., the strongest additive/synergistic correlation is 0.89 while the highest alternative signal is 0.96, meaning almost perfect correlation. This corroborates the hypothesis of parallel evolved, functionally equivalent compounds or structurally associated ones, i.e., an intermediate in the synthesis or a shunt product of the later.

Overall, the results of our analysis suggest that antimicrobial activity, particularly against *B. subtilis*, in *Rhododendron* is a composite biological response, i.e., implemented by combinatory effects. For both, additive/synergistic and alternative effects, there exist several peak pairs, whose categorical correlation coefficients outperform that of any individual LC-MS peak. On the contrary, there are hardly any indications for cytotoxic effects toward keratinocytes or enterocytes being implemented by pairwise combinations of LC-MS peaks (Figures S14, S15). With respect to HaCaT keratinocytes, it is to state that a correlation analysis does not need to be evaluated in detail as only one of the 87 different extracts from *Rhododendron* species exhibited significant cytotoxicity toward these. Accordingly, predictions of additive and synergistic as well as alternative effects would result in prediction of false-positive all-or-none reactions/mechanisms. In contrast, the individual peak analysis determining the predictive power of how likely a specific *Rhododendron* leaf-extract exerts cytotoxic effects toward intestine epithelial cells reveals only few moderate correlations. Likewise, peak combinations do not unfold a stronger predictive power because only moderate levels of correlation resulted from these analyses (Figure S16). These findings underline the notion of a wide spectrum and broad phytochemical composition of *Rhododendron* leaf extracts, and the necessity to analyze individual compounds more extensively.

## Discussion

Arguably, *Rhododendron* is a promising candidate plant for discovering bioactive agents. First of all, its ethno-medical applications for treating inflammation, pain, common cold symptoms, skin ailments, and gastro-intestinal disorders suggest the presence of effective substances (Popescu and Kopp, 2013). Moreover, the exceptional genetic diversity of the genus *Rhododendron* involves a large variety of different plant secondary compounds (Jaiswal et al., 2012). Thus, we assembled the, to our knowledge, largest compilation of a woody plant genus to systemically analyze potential bioactive effects of the plant extracts. In particular, we were interested in antimicrobial activity, especially against *B. subtilis* accompanied by a lack of cytotoxicity exerted toward mammalian cell lines. Furthermore, we aimed for determining the phytochemical origins of bioactivities, which is essential to design an antibiotic agent or food additive safe for the application to humans.

The present comparative analysis of nearly 90 *Rhododendron* species comprising microbiological, cell biological, phylogenetic, and phytochemical data assessed first of all their relationship to elucidate the potential origin of bioactive effects. The strongest relation has been revealed for antimicrobial activity and the phytochemical fingerprint of the examined species. Less marked is the observed signal for a connection of cytotoxicity and phytochemistry indicating that cytotoxicity as phytochemical response is more elusive or implemented more intricately. Contrary to the controversial hypothesis mentioned above (Hegnauer, 1986; Wink, 2003; Rønsted et al., 2012), we found no evidences that phylogeny determines the phytochemical composition in the genus *Rhododendron*. In the future, extended comprehensive examinations with larger numbers of taxa and a wider scope of metabolite analyses will be needed in order to substantiate the herein made observations and drawn conclusions.

While the utilization of crude leaf extracts renders it impossible to unambiguously identify bioactive agents, it, nevertheless, allows to predict candidate compounds and, thus, to make a first move into the proper direction. Especially the simultaneous assessment of antibacterial activity and cytotoxicity permits reasonable estimators. Overall, we suggested seven potentially antimicrobial active but non-cytotoxic compounds in terms of *m/z* retention time pairs. For one of these potential compounds, with an *m/z* value of 333 and retention time of 65 min, the antimicrobial activity against *B. subtilis* was experimentally confirmed via an exemplarily activity-guided fractionation of *Rhododendron ambiguum* b – one of the species with the highest antimicrobial susceptibility in the collection.

In the practice of lead compound screening from plant extracts, frequently a loss of activity is observed during activity-guided fractionations. Often this depletion was traced to be rationalized through combinatory effects, mostly additivity or synergy. To explore the possibility that bioactivities in *Rhododendron* can be ascribed to compound interactions or co-occurrence, we extended the categorical correlation analysis to pairs of LC-MS profiles via Boolean operators. For qualitative exploration of different interconnections, such Boolean modeling approaches had accomplished successful predictions with respect to disease-related biomarkers and drug combinations, respectively (Vaidya et al., 2008; Flobak et al., 2015). In similar fashion, we investigated pairwise synergies (realized via AND) and functional redundancies (OR) for antimicrobial activity as well as for cytotoxicity.

We found plausible indications for both, antimicrobial activity in *Rhododendron* being a synergistic response as well as being caused by functionally redundant compounds. On the one hand, the majority of potential additive or synergistic peak combinations, which outperform any individual peak, involve precisely one of the most-predictive individual peaks. This indicates those peak pairs comprise an accessory peak enhancing the activity. On the other hand, among the functionally equivalent compound combinations we found mostly peaks that did not show up in the individual peak analysis. Together with the considerable increase in the correlation signal strength, one is drawn to the conclusion that alternative effects such as functional redundancy reveal the profile of antimicrobial activity. In this regard, please note that the activity against *B. subtilis* can just serve as a benchmark. While such functional redundancy in defense compounds is known within individuals (Lipka et al., 2005), we are not aware of any report that has demonstrated this across species despite being frequently suggested.

Conversely, hardly any indication was found for combinatory effects explaining cytotoxicity toward mammalian keratinocytes and intestinal cells. However, it is important to note that the categorical analysis only accounts for the presence-absence profile and, thus, a potential phytochemical origin of cytotoxicity might be non-detectable. Moreover, in this study we have only investigated combinations of two LC-MS peaks although intricate responses such as bioactivity might also be assigned to more than two compounds.

Tracing literature-reported bioactivity of identified polyphenolics in the correlation analysis can demonstrate its predictive power. Caffeoylquinic acids, for instance, have been reported to act as efflux pump inhibitors in Gram-positive bacteria (Fiamegos et al., 2011; Kumar and Pooja Patial, 2016). Inhibition of the efflux pump AcrAB/TolC will result in accumulation of antibacterial substances within the cell and thus increase their intracellular concentration and their antibiotic effect. Six caffeoylquinic acids were identified in *Rhododendron* extracts at *m/z* value of 353.1 (Table S6). While in the individual analysis these compounds did not show up as potentially antimicrobial active, the extended correlation analysis using the Boolean operator AND points toward their accessory function. In combination with a caffeoylquinic acid more than 64% of the LC-MS peaks show an enhanced correlation signal. Amongst others, this includes the identified polyphenol previously suggested as best indicator for antimicrobial activity against *B. subtilis*, vanillic acid-*O*-hexoside (8.1 min). Showing that caffeoylquinic acids act additive to known weakly antibacterial compounds and, thus, enhance their biological effect underlines the value of the extended analysis.

Moreover, chlorogenic acids constitute a group of functionally equivalent natural products. In neuraminidase inhibition, the pharmacophore is located on the caffeic acid side chain and structural diversity within the core quinic acid does not significantly alter the observed inhibition constants (Karar et al., 2016). In line with this, the 15 identified regioisomers of chlorogenic acids (with *m/z* value 337.1, 353.1, and 367.1; Table S7) appear to act as alternative structures with identical effects in the extended correlation analysis. Particularly, more than 65% of their Boolean OR combinations outperform the individual peak correlation signals. The same argument might apply to the various known quercetin-*O*-glycosides, which might act as a prodrug and whose antibacterial effect does not depend on the structure of the attached sugar, that is usually cleaved off upon bacterial uptake (Biasutto and Zoratti, 2014). Within the *Rhododendron* extracts, 16 quercetin-*O*-glycosides were identified at *m/z* values of 433.1, 463.1, and 609.1 (Table S8). Together with quercetin itself, more than 82% of their OR combinations exceed the sum of corresponding individual peak correlations. The affirmation of regioisomeric chlorogenic acids and quercetin-*O*-glycosides as functional alternative structures reinforces the value of the extended analysis.

The next steps to be taken toward the identification of antimicrobial, well-tolerated agents are the exact determination of the chemical compounds and the quantification of the bioactive potential of the respective *Rhododendron* species. These steps will include activity-guided fractionations, subsequent compound purification and full structure elucidation of the other highly antimicrobial active *Rhododendron* species, regarding the respective tester organism. Following this, determination of minimal inhibitory concentration and minimal bactericidal concentration, as well as dose-response curves of cytotoxicity levels of each bioactive compound for relevant species will allow quantifying the respective bioactive potential of *Rhododendron*. Particular attention has to be devoted to the reproducibility of extraction quality and quantity of the *Rhododendron* leaf samples. The large amount of plant material, which is required to guarantee comparable results for this extraction-identification pipeline (approximately 150 g dry weight per plant), will force us to pre-select promising candidate fractions. By use of the predicted compound candidates, in terms of *m/z* value and retention time, we expect a noticeably improved economy of time and resources for the final identification of *Rhododendron*-derived bioactive substances.

To our best knowledge, this is the most comprehensive investigation of bioactivity in a single plant genus. Apart from antimicrobial activity against *B. subtilis* and cytotoxicity exerted toward mammalian cell lines, the search for the phylogenetic and phytochemical origin of bioactivity was addressed. Through the combination of experimental and systems biology approaches we are now capable of scanning and correlating high-throughput LC-MS data to predict seven potentially antimicrobial active but non-cytotoxic compounds from the subgenus *Rhododendron* and of exploring synergistic effects and functional redundancy of antimicrobial activity in this plant genus. We believe that this integrative approach may provide the foundation not only for transferring the methodology to other plant genera known for applications of their bioactive extract such as ginseng (Cowan, 1999), but also for pushing forward the resource efficient identification of natural product-based drugs.

## Author contributions

MU, NK, KB, and DA designed and supervised the study; AS, AR, DA, and IHS performed the experiments; HS contributed material; AG, SG, and MH analyzed the data; and AG and MU wrote the manuscript.

### Conflict of interest statement

The authors declare that the research was conducted in the absence of any commercial or financial relationships that could be construed as a potential conflict of interest. The reviewer KT and handling Editor declared their shared affiliation, and the handling Editor states that the process nevertheless met the standards of a fair and objective review.
