# Supplementary material for: Bioactivity in Rhododendron: A Systemic Analysis of Antimicrobial and Cytotoxic Activities and Their Phylogenetic and Phytochemical Origins
Source: Front Plant Sci. 2017 Apr 13;8:551. doi: 10.3389/fpls.2017.00551 (PMC5390042; doi:10.3389/fpls.2017.00551)
Supplement: Supplementary file 1 [file SupplementaryMaterial.pdf]

Supplementary Material:

Bioactivity in *Rhododendron*: A systemic analysis of antimicrobial and cytotoxic activities and their phylogenetic and phytochemical origins

Anne Grimbs[1], Abhinandan Shrestha[1], Ahmed Sayed Deyab Rezk[1], Sergio Grimbs[1], Inamullah Hakeem Said[1], Hartwig Schepker[2], Marc-Thorsten Hütt[1], Dirk Carl Albach[3], Klaudia Brix[1], Nikolai Kuhnert[1] and Matthias S. Ullrich[1,*]

[1]Department for Life Sciences & Chemistry, Jacobs University Bremen, Bremen, Germany
[2]Stiftung Bremer Rhododendronpark, Bremen, Germany
[3]Institute for Biology and Environmental Sciences, Carl von Ossietzky University Oldenburg, Oldenburg, Germany

List of Figures

S1 PCA scores plots colored with respect to cytotoxicity classifications . . . 2
S2 PCA loadings plot . . . 2
S3 Chemical diversity across the subgenera of *Rhododendron* . . . 3
S4 Box plots of 282 identified polyphenolics by antimicrobial activity classification . . . 12
S5 Box plots of all 292 identified polyphenolics by cytotoxicity classification towards HaCaT cells . . . 22
S6 Box plots of all 292 identified polyphenolics by cytotoxicity classification towards IEC-6 cells . . . 31
S7 Distribution of the detected LC-MS peaks regarding *m/z* ratios and retention times . . . 32
S8 The 25 most- and least-predictive LC-MS peaks regarding Cohen's κ for cytotoxicity towards IEC-6 cells . . . 33
S9 The 25 most- and least-predictive LC-MS peaks regarding Cohen's κ for cytotoxicity towards HaCaT cells . . . 34
S10 The 27 most-predictive LC-MS peaks regarding Cohen's κ for antimicrobial activity and non-cytotoxicity towards both cell lines . . . 36
S11 The 50 most-predictive LC-MS peak combinations with additive effects regarding Cohen's κ for antimicrobial activity . . . 38
S12 The 50 most-predictive LC-MS peak combinations with alternative effects regarding Cohen's κ for antimicrobial activity . . . 39
S13 Heatmap of the most-predictive LC-MS peak combinations with alternative effects regarding Cohen's κ for antimicrobial activity . . . 40
S14 The 50 most-predictive LC-MS peak combinations with additive effects regarding Cohen's κ for cytotoxicity towards IEC-6 cells . . . 41
S15 The 50 most-predictive LC-MS peak combinations with alternative effects regarding Cohen's κ for cytotoxicity towards IEC-6 cells . . . 42
S16 Heatmap of the most-predictive LC-MS peak combinations with additive and alternative effects, respectively, regarding Cohen's κ for cytotoxicity towards IEC-6 cells . . . 43

List of Tables

S1 Average and standard deviation of *m/z* ratios and retention times for most-predictive LC-MS peaks . 32
S2 The 23 most-predictive LC-MS peaks regarding Cohen's κ for antimicrobial activity . . . 35
S3 The 27 most-predictive LC-MS peaks regarding Cohen's κ for antimicrobial activity and non-cytotoxicity 37
S4 The 12 out of 23 most-predictive individual LC-MS peaks involved in the most-predictive additive peak combinations regarding antimicrobial activity . . . 44
S5 The 23 most-predictive individual LC-MS peaks involved in the most-predictive alternative peak combinations regarding antimicrobial activity . . . 45
S6 The 6 identified caffeoylquinic acids acting additive in LC-MS peak combinations . . . 45
S7 The 15 identified regioisomeric chlorogenic acids acting as functional alternative structures in LC-MS peak combinations . . . 46
S8 Quercetin and the 16 identified quercetin-O-glycosides acting as functional alternative structures in LC-MS peak combinations . . . 46
S9 Sequence generation for phylogenetic analysis including DNA regions, primer sequences, and respective PCR protocols . . . 47

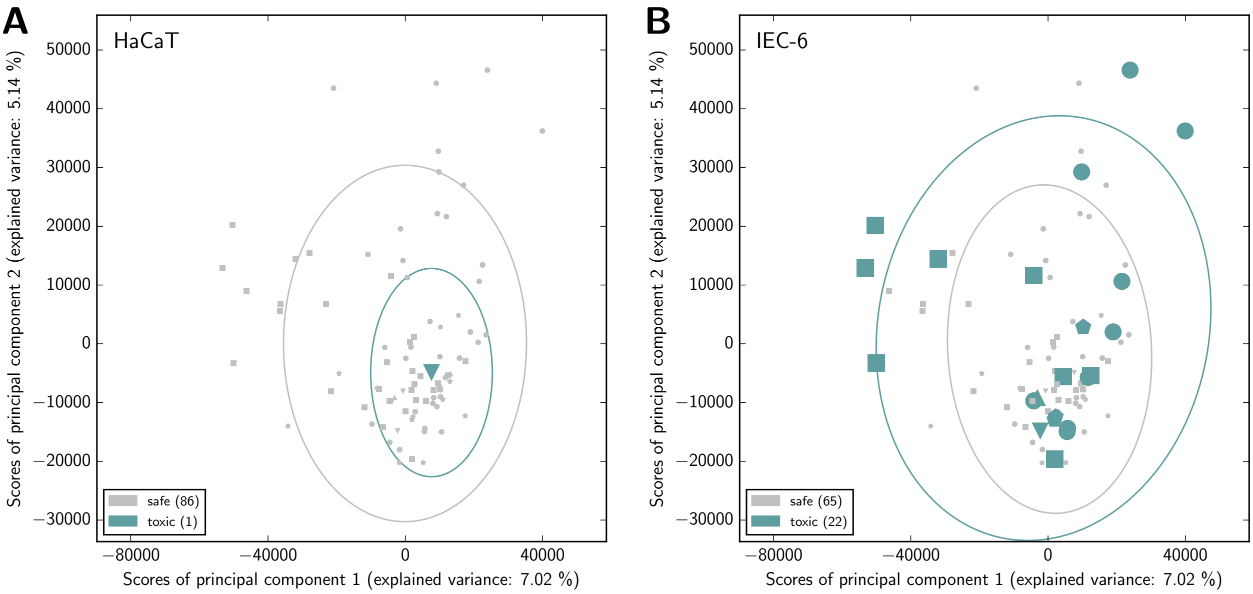

Figure S1 | Principal component analysis (using Pareto scaling) of the phytochemical data for all 87 *Rhododendron* species. The scores of the principal components corresponding to the species are colored with respect to the cytotoxicity towards HaCaT cells (**A**) and IEC-6 cells (**B**), respectively. The item shape highlights the subgenus of each species (△:*Azaleastrum*, □:*Hymenanthes*, ⬠:*Pentanthera*, ○:*Rhododendron*, ▽:*Tsutsusi*). The item size as well illustrates the cytotoxicity classification, toxic – large and turquoise, safe – small and gray.

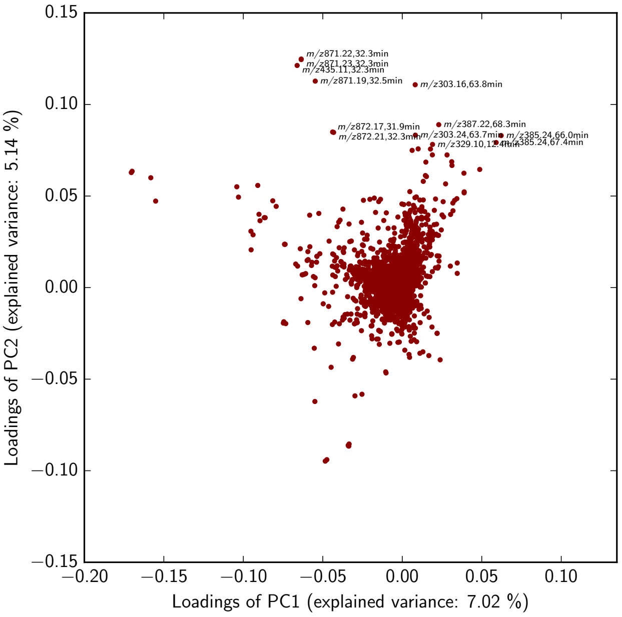

Figure S2 | Loadings of the principal component analysis of the phytochemical data of all 87 *Rhododendron* species. Labeled are the 12 peaks (defined by mass-to-charge ratio and retention time) which are most related to the antimicrobial active *Rhododendron* samples based on the underlying separation.

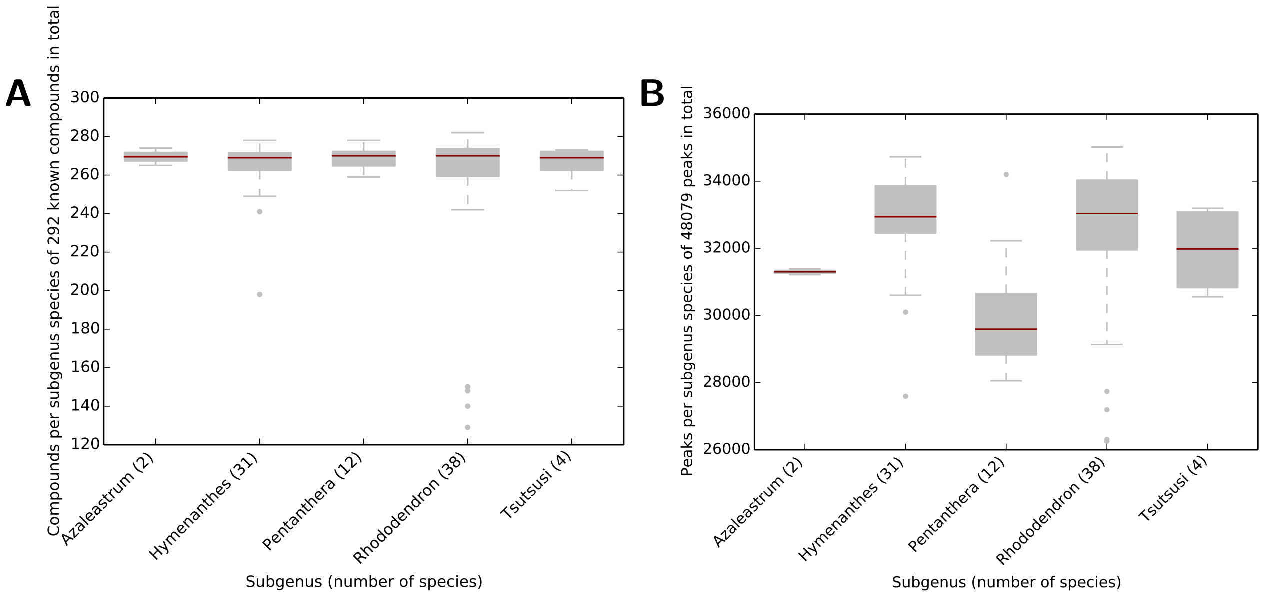

Figure S3 | Chemical diversity with respect to the subgenus classification of the 87 *Rhododendron* species in terms of total number of identified polyphenolics (A) and of detected LC-MS peaks (B).

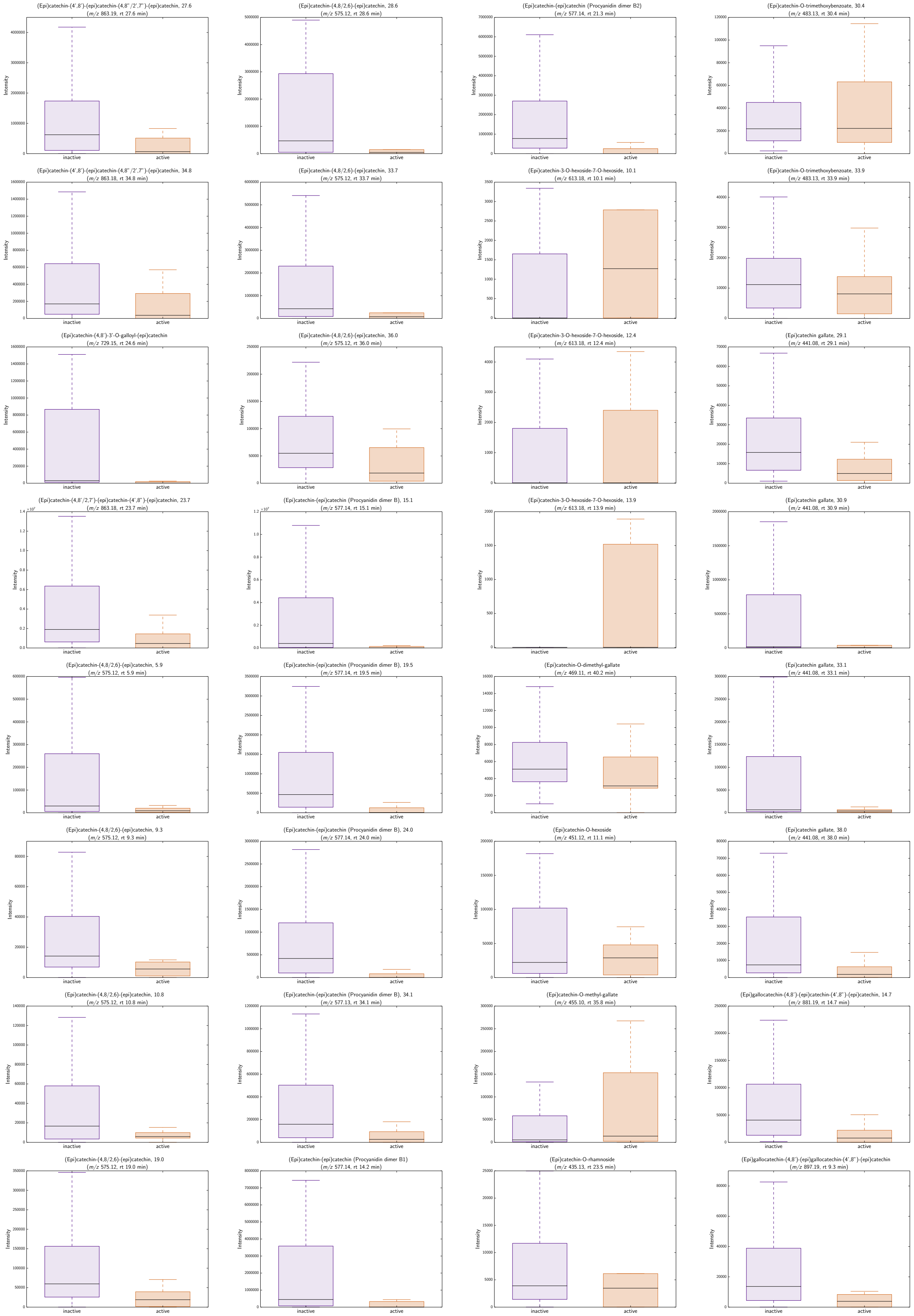
(Epi)catechin-(4',8')-(epi)catechin-(4,8''/2',7'')-(epi)catechin, 27.6
(m/z 863.19, rt 27.6 min)
(Epi)catechin-(4,8/2,6)-(epi)catechin, 28.6
(m/z 575.12, rt 28.6 min)
(Epi)catechin-(epi)catechin (Procyanidin dimer B2)
(m/z 577.14, rt 21.3 min)
(Epi)catechin-O-trimethoxybenzoate, 30.4
(m/z 483.13, rt 30.4 min)
(Epi)catechin-(4',8')-(epi)catechin-(4,8''/2',7'')-(epi)catechin, 34.8
(m/z 863.18, rt 34.8 min)
(Epi)catechin-(4,8/2,6)-(epi)catechin, 33.7
(m/z 575.12, rt 33.7 min)
(Epi)catechin-3-O-hexoside-7-O-hexoside, 10.1
(m/z 613.18, rt 10.1 min)
(Epi)catechin-O-trimethoxybenzoate, 33.9
(m/z 483.13, rt 33.9 min)
(Epi)catechin-(4,8')-3'-O-galloyl-(epi)catechin
(m/z 729.15, rt 24.6 min)
(Epi)catechin-(4,8/2,6)-(epi)catechin, 36.0
(m/z 575.12, rt 36.0 min)
(Epi)catechin-3-O-hexoside-7-O-hexoside, 12.4
(m/z 613.18, rt 12.4 min)
(Epi)catechin gallate, 29.1
(m/z 441.08, rt 29.1 min)
(Epi)catechin-(4,8'/2,7')-(epi)catechin-(4',8'')-(epi)catechin, 23.7
(m/z 863.18, rt 23.7 min)
(Epi)catechin-(epi)catechin (Procyanidin dimer B), 15.1
(m/z 577.14, rt 15.1 min)
(Epi)catechin-3-O-hexoside-7-O-hexoside, 13.9
(m/z 613.18, rt 13.9 min)
(Epi)catechin gallate, 30.9
(m/z 441.08, rt 30.9 min)
(Epi)catechin-(4,8/2,6)-(epi)catechin, 5.9
(m/z 575.12, rt 5.9 min)
(Epi)catechin-(epi)catechin (Procyanidin dimer B), 19.5
(m/z 577.14, rt 19.5 min)
(Epi)catechin-O-dimethyl-gallate
(m/z 469.11, rt 40.2 min)
(Epi)catechin gallate, 33.1
(m/z 441.08, rt 33.1 min)
(Epi)catechin-(4,8/2,6)-(epi)catechin, 9.3
(m/z 575.12, rt 9.3 min)
(Epi)catechin-(epi)catechin (Procyanidin dimer B), 24.0
(m/z 577.14, rt 24.0 min)
(Epi)catechin-O-hexoside
(m/z 451.12, rt 11.1 min)
(Epi)catechin gallate, 38.0
(m/z 441.08, rt 38.0 min)
(Epi)catechin-(4,8/2,6)-(epi)catechin, 10.8
(m/z 575.12, rt 10.8 min)
(Epi)catechin-(epi)catechin (Procyanidin dimer B), 34.1
(m/z 577.13, rt 34.1 min)
(Epi)catechin-O-methyl-gallate
(m/z 455.10, rt 35.8 min)
(Epi)gallocatechin-(4,8')-(epi)catechin-(4',8'')-(epi)catechin, 14.7
(m/z 881.19, rt 14.7 min)
(Epi)catechin-(4,8/2,6)-(epi)catechin, 19.0
(m/z 575.12, rt 19.0 min)
(Epi)catechin-(epi)catechin (Procyanidin dimer B1)
(m/z 577.14, rt 14.2 min)
(Epi)catechin-O-rhamnoside
(m/z 435.13, rt 23.5 min)
(Epi)gallocatechin-(4,8')-(epi)gallocatechin-(4',8'')-(epi)catechin
(m/z 897.19, rt 9.3 min)
Intensity
inactive
active

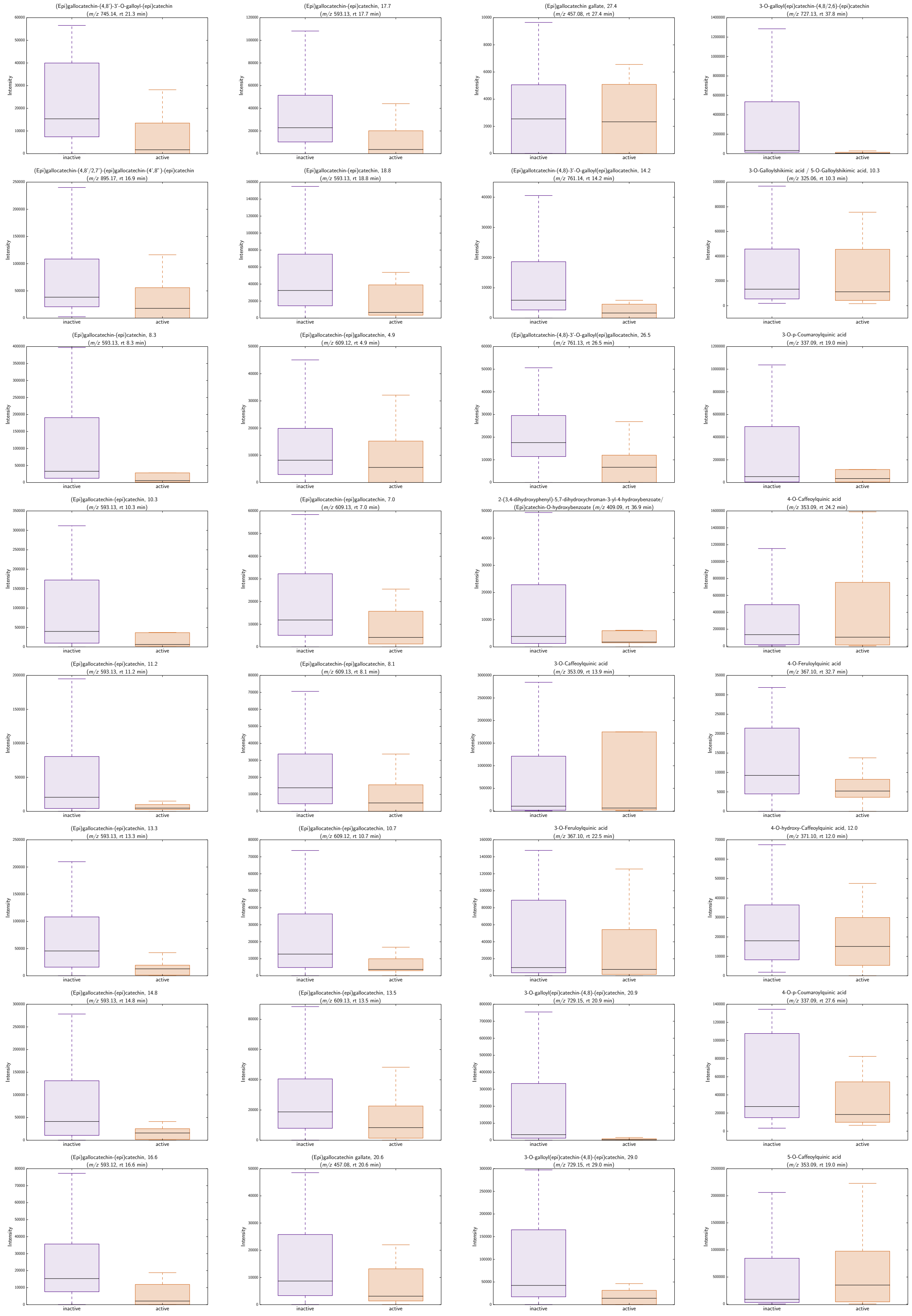
(Epi)gallocatechin-(4,8')-3'-O-galloyl-(epi)catechin
(m/z 745.14, rt 21.3 min)
(Epi)gallocatechin-(epi)catechin, 17.7
(m/z 593.13, rt 17.7 min)
(Epi)gallocatechin gallate, 27.4
(m/z 457.08, rt 27.4 min)
3-O-galloyl(epi)catechin-(4,8/2,6)-(epi)catechin
(m/z 727.13, rt 37.8 min)
(Epi)gallocatechin-(4,8'/2,7')-(epi)gallocatechin-(4',8'')-(epi)catechin
(m/z 895.17, rt 16.9 min)
(Epi)gallocatechin-(epi)catechin, 18.8
(m/z 593.13, rt 18.8 min)
(Epi)gallotcatechin-(4,8)-3'-O-galloyl(epi)gallocatechin, 14.2
(m/z 761.14, rt 14.2 min)
3-O-Galloylshikimic acid / 5-O-Galloylshikimic acid, 10.3
(m/z 325.06, rt 10.3 min)
(Epi)gallocatechin-(epi)catechin, 8.3
(m/z 593.13, rt 8.3 min)
(Epi)gallocatechin-(epi)gallocatechin, 4.9
(m/z 609.12, rt 4.9 min)
(Epi)gallotcatechin-(4,8)-3'-O-galloyl(epi)gallocatechin, 26.5
(m/z 761.13, rt 26.5 min)
3-O-p-Coumaroylquinic acid
(m/z 337.09, rt 19.0 min)
(Epi)gallocatechin-(epi)catechin, 10.3
(m/z 593.13, rt 10.3 min)
(Epi)gallocatechin-(epi)gallocatechin, 7.0
(m/z 609.13, rt 7.0 min)
2-(3,4-dihydroxyphenyl)-5,7-dihydroxychroman-3-yl-4-hydroxybenzoate/
(Epi)catechin-O-hydroxybenzoate (m/z 409.09, rt 36.9 min)
4-O-Caffeoylquinic acid
(m/z 353.09, rt 24.2 min)
(Epi)gallocatechin-(epi)catechin, 11.2
(m/z 593.13, rt 11.2 min)
(Epi)gallocatechin-(epi)gallocatechin, 8.1
(m/z 609.13, rt 8.1 min)
3-O-Caffeoylquinic acid
(m/z 353.09, rt 13.9 min)
4-O-Feruloylquinic acid
(m/z 367.10, rt 32.7 min)
(Epi)gallocatechin-(epi)catechin, 13.3
(m/z 593.13, rt 13.3 min)
(Epi)gallocatechin-(epi)gallocatechin, 10.7
(m/z 609.12, rt 10.7 min)
3-O-Feruloylquinic acid
(m/z 367.10, rt 22.5 min)
4-O-hydroxy-Caffeoylquinic acid, 12.0
(m/z 371.10, rt 12.0 min)
(Epi)gallocatechin-(epi)catechin, 14.8
(m/z 593.13, rt 14.8 min)
(Epi)gallocatechin-(epi)gallocatechin, 13.5
(m/z 609.13, rt 13.5 min)
3-O-galloyl(epi)catechin-(4,8)-(epi)catechin, 20.9
(m/z 729.15, rt 20.9 min)
4-O-p-Coumaroylquinic acid
(m/z 337.09, rt 27.6 min)
(Epi)gallocatechin-(epi)catechin, 16.6
(m/z 593.12, rt 16.6 min)
(Epi)gallocatechin gallate, 20.6
(m/z 457.08, rt 20.6 min)
3-O-galloyl(epi)catechin-(4,8)-(epi)catechin, 29.0
(m/z 729.15, rt 29.0 min)
5-O-Caffeoylquinic acid
(m/z 353.09, rt 19.0 min)
Intensity
inactive
active

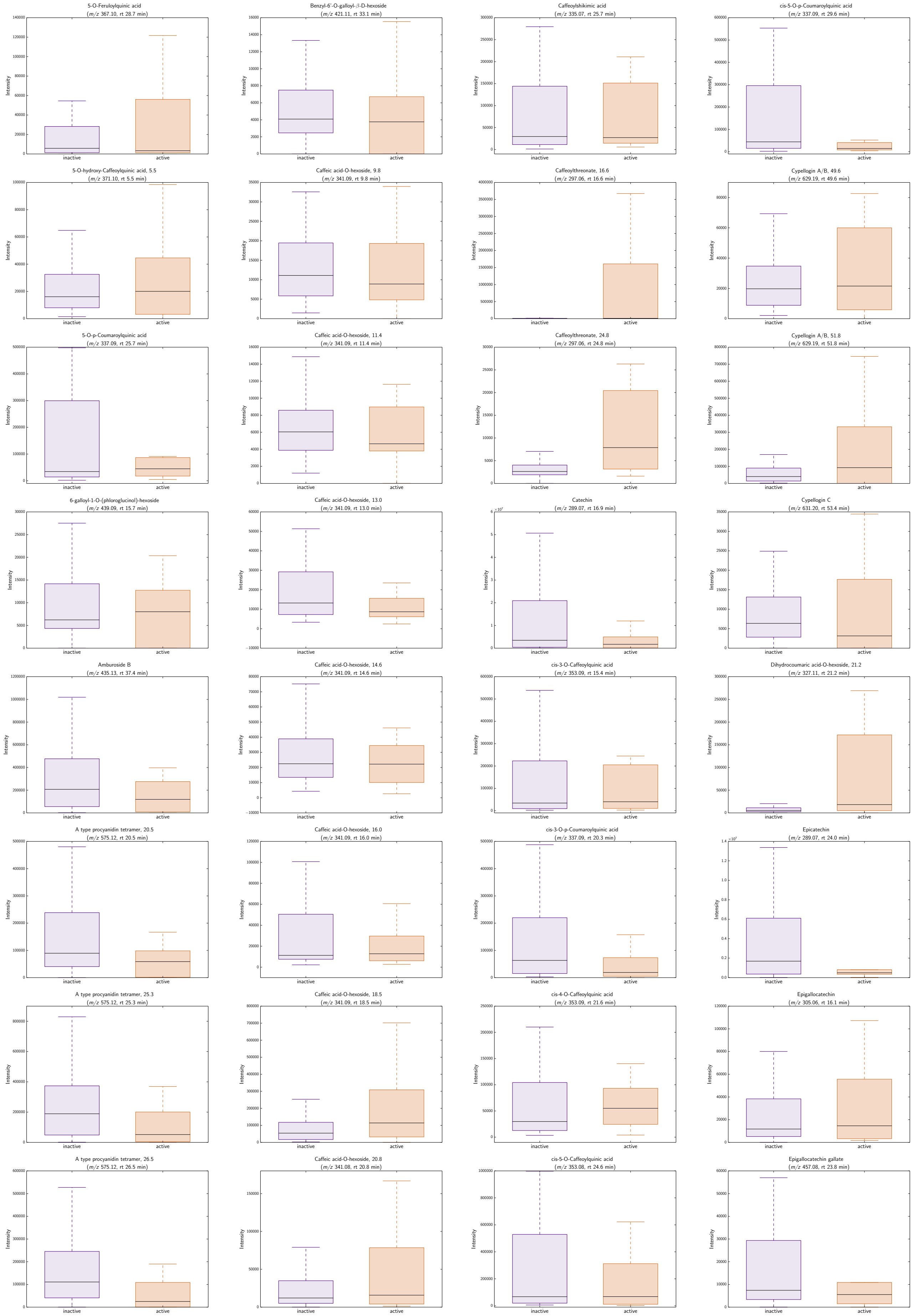
5-O-Feruloylquinic acid (m/z 367.10, rt 28.7 min)
Benzyl-6'-O-galloyl-β-D-hexoside (m/z 421.11, rt 33.1 min)
Caffeoylshikimic acid (m/z 335.07, rt 25.7 min)
cis-5-O-p-Coumaroylquinic acid (m/z 337.09, rt 29.6 min)
5-O-hydroxy-Caffeoylquinic acid, 5.5 (m/z 371.10, rt 5.5 min)
Caffeic acid-O-hexoside, 9.8 (m/z 341.09, rt 9.8 min)
Caffeoylthreonate, 16.6 (m/z 297.06, rt 16.6 min)
Cypellogin A/B, 49.6 (m/z 629.19, rt 49.6 min)
5-O-p-Coumaroylquinic acid (m/z 337.09, rt 25.7 min)
Caffeic acid-O-hexoside, 11.4 (m/z 341.09, rt 11.4 min)
Caffeoylthreonate, 24.8 (m/z 297.06, rt 24.8 min)
Cypellogin A/B, 51.8 (m/z 629.19, rt 51.8 min)
6-galloyl-1-O-(phloroglucinol)-hexoside (m/z 439.09, rt 15.7 min)
Caffeic acid-O-hexoside, 13.0 (m/z 341.09, rt 13.0 min)
Catechin (m/z 289.07, rt 16.9 min)
Cypellogin C (m/z 631.20, rt 53.4 min)
Amburoside B (m/z 435.13, rt 37.4 min)
Caffeic acid-O-hexoside, 14.6 (m/z 341.09, rt 14.6 min)
cis-3-O-Caffeoylquinic acid (m/z 353.09, rt 15.4 min)
Dihydrocoumaric acid-O-hexoside, 21.2 (m/z 327.11, rt 21.2 min)
A type procyanidin tetramer, 20.5 (m/z 575.12, rt 20.5 min)
Caffeic acid-O-hexoside, 16.0 (m/z 341.09, rt 16.0 min)
cis-3-O-p-Coumaroylquinic acid (m/z 337.09, rt 20.3 min)
Epicatechin (m/z 289.07, rt 24.0 min)
A type procyanidin tetramer, 25.3 (m/z 575.12, rt 25.3 min)
Caffeic acid-O-hexoside, 18.5 (m/z 341.09, rt 18.5 min)
cis-4-O-Caffeoylquinic acid (m/z 353.09, rt 21.6 min)
Epigallocatechin (m/z 305.06, rt 16.1 min)
A type procyanidin tetramer, 26.5 (m/z 575.12, rt 26.5 min)
Caffeic acid-O-hexoside, 20.8 (m/z 341.08, rt 20.8 min)
cis-5-O-Caffeoylquinic acid (m/z 353.08, rt 24.6 min)
Epigallocatechin gallate (m/z 457.08, rt 23.8 min)
Intensity
inactive
active

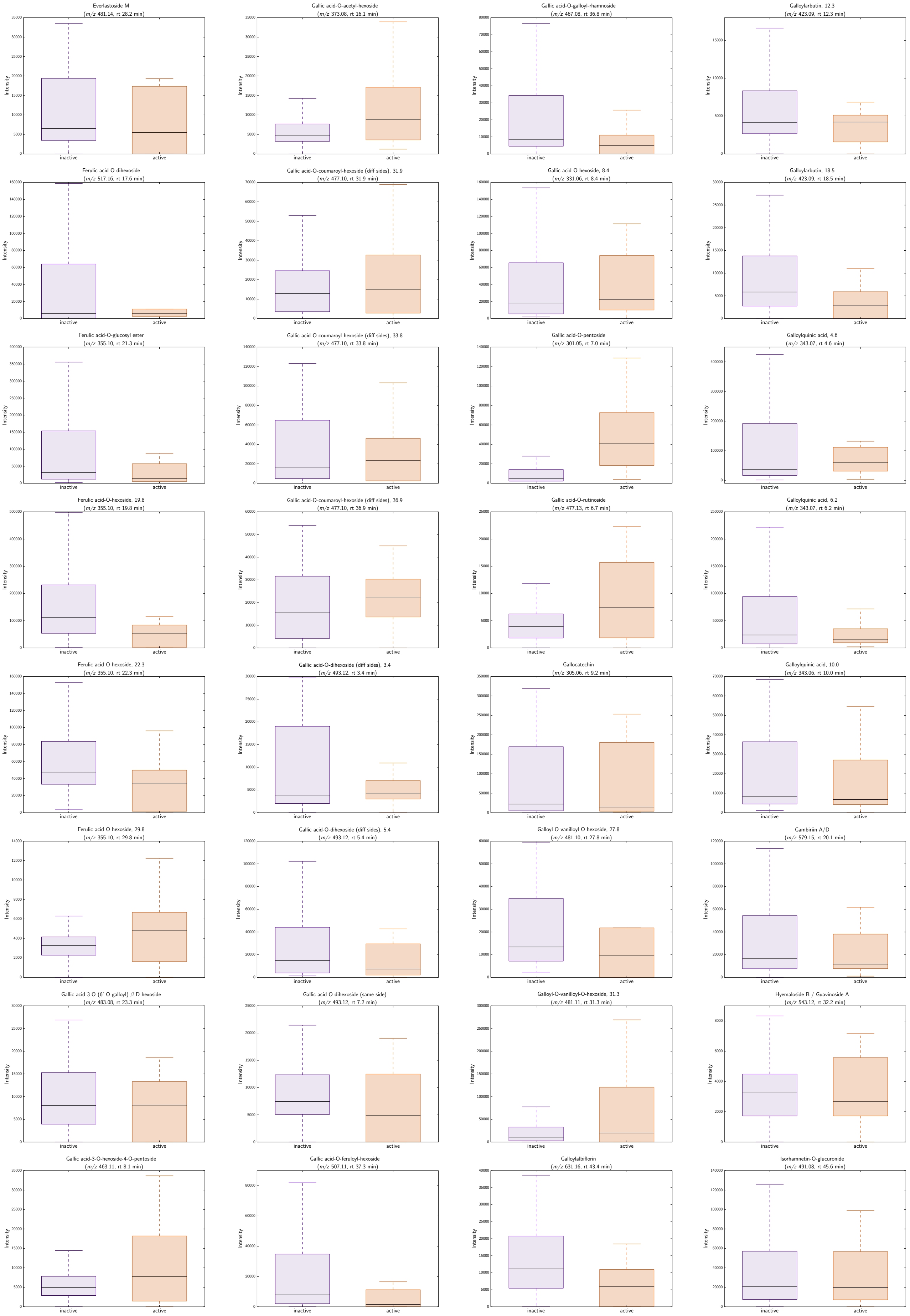
Everlastoside M
(m/z 481.14, rt 28.2 min)
Gallic acid-O-acetyl-hexoside
(m/z 373.08, rt 16.1 min)
Gallic acid-O-galloyl-rhamnoside
(m/z 467.08, rt 36.8 min)
Galloylarbutin, 12.3
(m/z 423.09, rt 12.3 min)
Ferulic acid-O-dihexoside
(m/z 517.16, rt 17.6 min)
Gallic acid-O-coumaroyl-hexoside (diff sides), 31.9
(m/z 477.10, rt 31.9 min)
Gallic acid-O-hexoside, 8.4
(m/z 331.06, rt 8.4 min)
Galloylarbutin, 18.5
(m/z 423.09, rt 18.5 min)
Ferulic acid-O-glucosyl ester
(m/z 355.10, rt 21.3 min)
Gallic acid-O-coumaroyl-hexoside (diff sides), 33.8
(m/z 477.10, rt 33.8 min)
Gallic acid-O-pentoside
(m/z 301.05, rt 7.0 min)
Galloylquinic acid, 4.6
(m/z 343.07, rt 4.6 min)
Ferulic acid-O-hexoside, 19.8
(m/z 355.10, rt 19.8 min)
Gallic acid-O-coumaroyl-hexoside (diff sides), 36.9
(m/z 477.10, rt 36.9 min)
Gallic acid-O-rutinoside
(m/z 477.13, rt 6.7 min)
Galloylquinic acid, 6.2
(m/z 343.07, rt 6.2 min)
Ferulic acid-O-hexoside, 22.3
(m/z 355.10, rt 22.3 min)
Gallic acid-O-dihexoside (diff sides), 3.4
(m/z 493.12, rt 3.4 min)
Gallocatechin
(m/z 305.06, rt 9.2 min)
Galloylquinic acid, 10.0
(m/z 343.06, rt 10.0 min)
Ferulic acid-O-hexoside, 29.8
(m/z 355.10, rt 29.8 min)
Gallic acid-O-dihexoside (diff sides), 5.4
(m/z 493.12, rt 5.4 min)
Galloyl-O-vanilloyl-O-hexoside, 27.8
(m/z 481.10, rt 27.8 min)
Gambiriin A/D
(m/z 579.15, rt 20.1 min)
Gallic acid-3-O-(6'-O-galloyl)-β-D-hexoside
(m/z 483.08, rt 23.3 min)
Gallic acid-O-dihexoside (same side)
(m/z 493.12, rt 7.2 min)
Galloyl-O-vanilloyl-O-hexoside, 31.3
(m/z 481.11, rt 31.3 min)
Hyemaloside B / Guavinoside A
(m/z 543.12, rt 32.2 min)
Gallic acid-3-O-hexoside-4-O-pentoside
(m/z 463.11, rt 8.1 min)
Gallic acid-O-feruloyl-hexoside
(m/z 507.11, rt 37.3 min)
Galloylalbiflorin
(m/z 631.16, rt 43.4 min)
Isorhamnetin-O-glucuronide
(m/z 491.08, rt 45.6 min)
Intensity
inactive
active

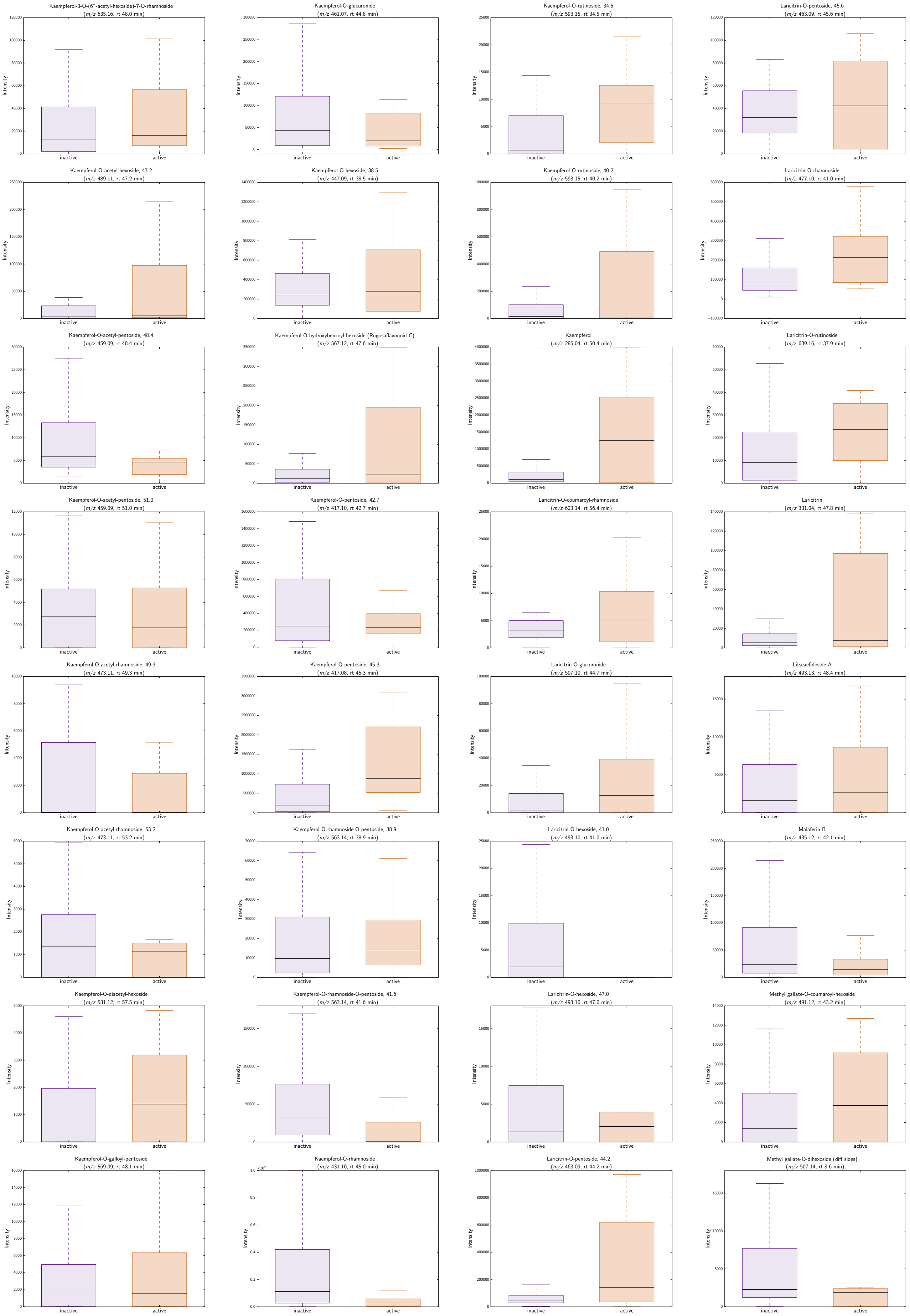
Kaempferol-3-O-(6''-acetyl-hexoside)-7-O-rhamnoside
(m/z 635.16, rt 48.0 min)
Kaempferol-O-glucuronide
(m/z 461.07, rt 44.8 min)
Kaempferol-O-rutinoside, 34.5
(m/z 593.15, rt 34.5 min)
Laricitrin-O-pentoside, 45.6
(m/z 463.09, rt 45.6 min)
Kaempferol-O-acetyl-hexoside, 47.2
(m/z 489.11, rt 47.2 min)
Kaempferol-O-hexoside, 38.5
(m/z 447.09, rt 38.5 min)
Kaempferol-O-rutinoside, 40.2
(m/z 593.15, rt 40.2 min)
Laricitrin-O-rhamnoside
(m/z 477.10, rt 41.0 min)
Kaempferol-O-acetyl-pentoside, 48.4
(m/z 459.09, rt 48.4 min)
Kaempferol-O-hydroxybenzoyl-hexoside (Rugosaflavonoid C)
(m/z 567.12, rt 47.6 min)
Kaempferol
(m/z 285.04, rt 50.4 min)
Laricitrin-O-rutinoside
(m/z 639.16, rt 37.9 min)
Kaempferol-O-acetyl-pentoside, 51.0
(m/z 459.09, rt 51.0 min)
Kaempferol-O-pentoside, 42.7
(m/z 417.10, rt 42.7 min)
Laricitrin-O-coumaroyl-rhamnoside
(m/z 623.14, rt 56.4 min)
Laricitrin
(m/z 331.04, rt 47.8 min)
Kaempferol-O-acetyl-rhamnoside, 49.3
(m/z 473.11, rt 49.3 min)
Kaempferol-O-pentoside, 45.3
(m/z 417.08, rt 45.3 min)
Laricitrin-O-glucoronide
(m/z 507.10, rt 44.7 min)
Litseaefoloside A
(m/z 493.13, rt 48.4 min)
Kaempferol-O-acetyl-rhamnoside, 53.2
(m/z 473.11, rt 53.2 min)
Kaempferol-O-rhamnoside-O-pentoside, 38.9
(m/z 563.14, rt 38.9 min)
Laricitrin-O-hexoside, 41.0
(m/z 493.10, rt 41.0 min)
Malaferin B
(m/z 435.12, rt 42.1 min)
Kaempferol-O-diacetyl-hexoside
(m/z 531.12, rt 57.5 min)
Kaempferol-O-rhamnoside-O-pentoside, 41.6
(m/z 563.14, rt 41.6 min)
Laricitrin-O-hexoside, 47.0
(m/z 493.10, rt 47.0 min)
Methyl gallate-O-coumaroyl-hexoside
(m/z 491.12, rt 43.2 min)
Kaempferol-O-galloyl-pentoside
(m/z 569.09, rt 48.1 min)
Kaempferol-O-rhamnoside
(m/z 431.10, rt 45.0 min)
Laricitrin-O-pentoside, 44.2
(m/z 463.09, rt 44.2 min)
Methyl gallate-O-dihexoside (diff sides)
(m/z 507.14, rt 8.6 min)
Intensity
inactive
active

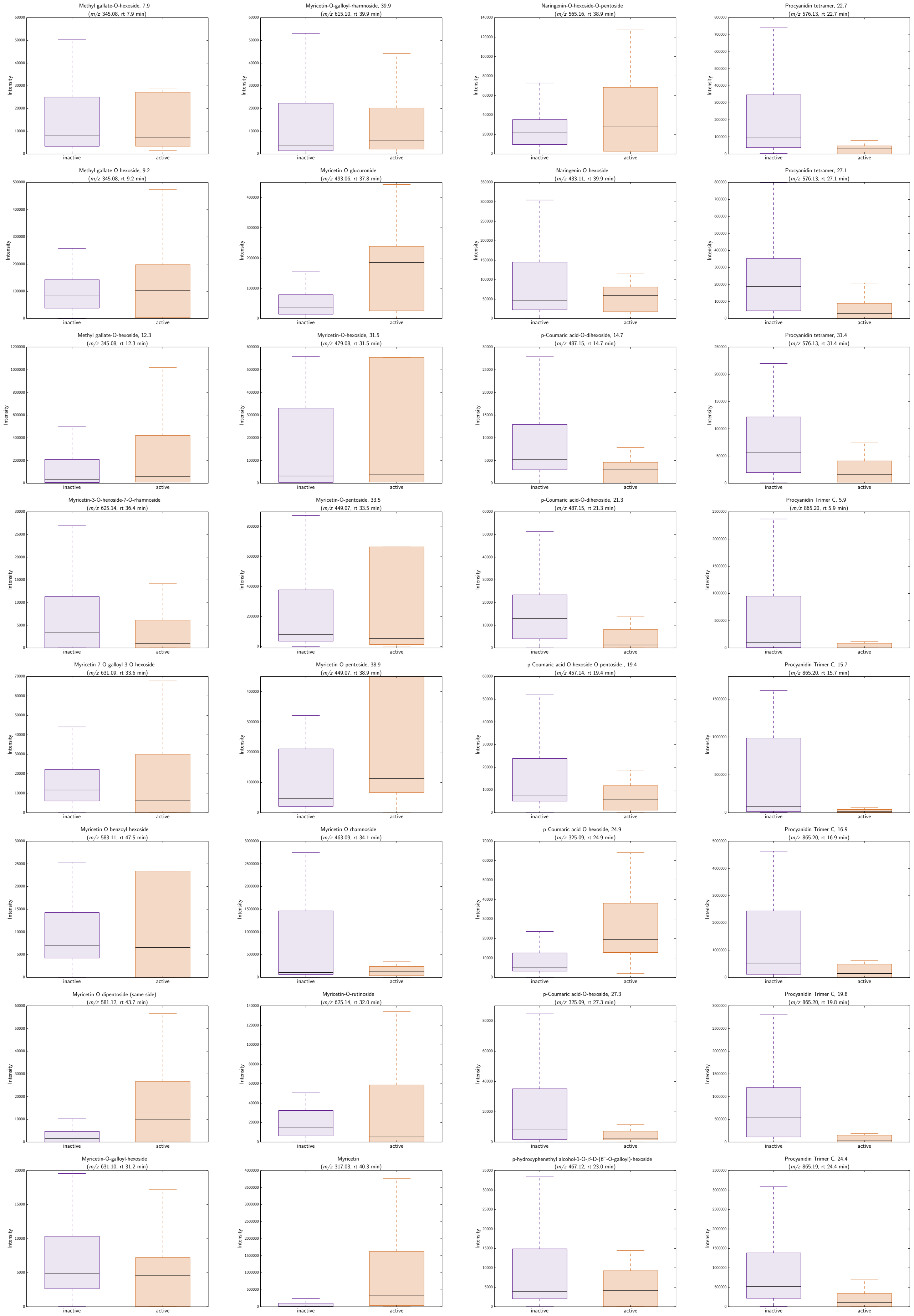
Methyl gallate-O-hexoside, 7.9
(m/z 345.08, rt 7.9 min)
Myricetin-O-galloyl-rhamnoside, 39.9
(m/z 615.10, rt 39.9 min)
Naringenin-O-hexoside-O-pentoside
(m/z 565.16, rt 38.9 min)
Procyanidin tetramer, 22.7
(m/z 576.13, rt 22.7 min)
Methyl gallate-O-hexoside, 9.2
(m/z 345.08, rt 9.2 min)
Myricetin-O-glucuronide
(m/z 493.06, rt 37.8 min)
Naringenin-O-hexoside
(m/z 433.11, rt 39.9 min)
Procyanidin tetramer, 27.1
(m/z 576.13, rt 27.1 min)
Methyl gallate-O-hexoside, 12.3
(m/z 345.08, rt 12.3 min)
Myricetin-O-hexoside, 31.5
(m/z 479.08, rt 31.5 min)
p-Coumaric acid-O-dihexoside, 14.7
(m/z 487.15, rt 14.7 min)
Procyanidin tetramer, 31.4
(m/z 576.13, rt 31.4 min)
Myricetin-3-O-hexoside-7-O-rhamnoside
(m/z 625.14, rt 36.4 min)
Myricetin-O-pentoside, 33.5
(m/z 449.07, rt 33.5 min)
p-Coumaric acid-O-dihexoside, 21.3
(m/z 487.15, rt 21.3 min)
Procyanidin Trimer C, 5.9
(m/z 865.20, rt 5.9 min)
Myricetin-7-O-galloyl-3-O-hexoside
(m/z 631.09, rt 33.6 min)
Myricetin-O-pentoside, 38.9
(m/z 449.07, rt 38.9 min)
p-Coumaric acid-O-hexoside-O-pentoside , 19.4
(m/z 457.14, rt 19.4 min)
Procyanidin Trimer C, 15.7
(m/z 865.20, rt 15.7 min)
Myricetin-O-benzoyl-hexoside
(m/z 583.11, rt 47.5 min)
Myricetin-O-rhamnoside
(m/z 463.09, rt 34.1 min)
p-Coumaric acid-O-hexoside, 24.9
(m/z 325.09, rt 24.9 min)
Procyanidin Trimer C, 16.9
(m/z 865.20, rt 16.9 min)
Myricetin-O-dipentoside (same side)
(m/z 581.12, rt 43.7 min)
Myricetin-O-rutinoside
(m/z 625.14, rt 32.0 min)
p-Coumaric acid-O-hexoside, 27.3
(m/z 325.09, rt 27.3 min)
Procyanidin Trimer C, 19.8
(m/z 865.20, rt 19.8 min)
Myricetin-O-galloyl-hexoside
(m/z 631.10, rt 31.2 min)
Myricetin
(m/z 317.03, rt 40.3 min)
p-hydroxyphenethyl alcohol-1-O-β-D-(6''-O-galloyl)-hexoside
(m/z 467.12, rt 23.0 min)
Procyanidin Trimer C, 24.4
(m/z 865.19, rt 24.4 min)
Intensity
inactive
active

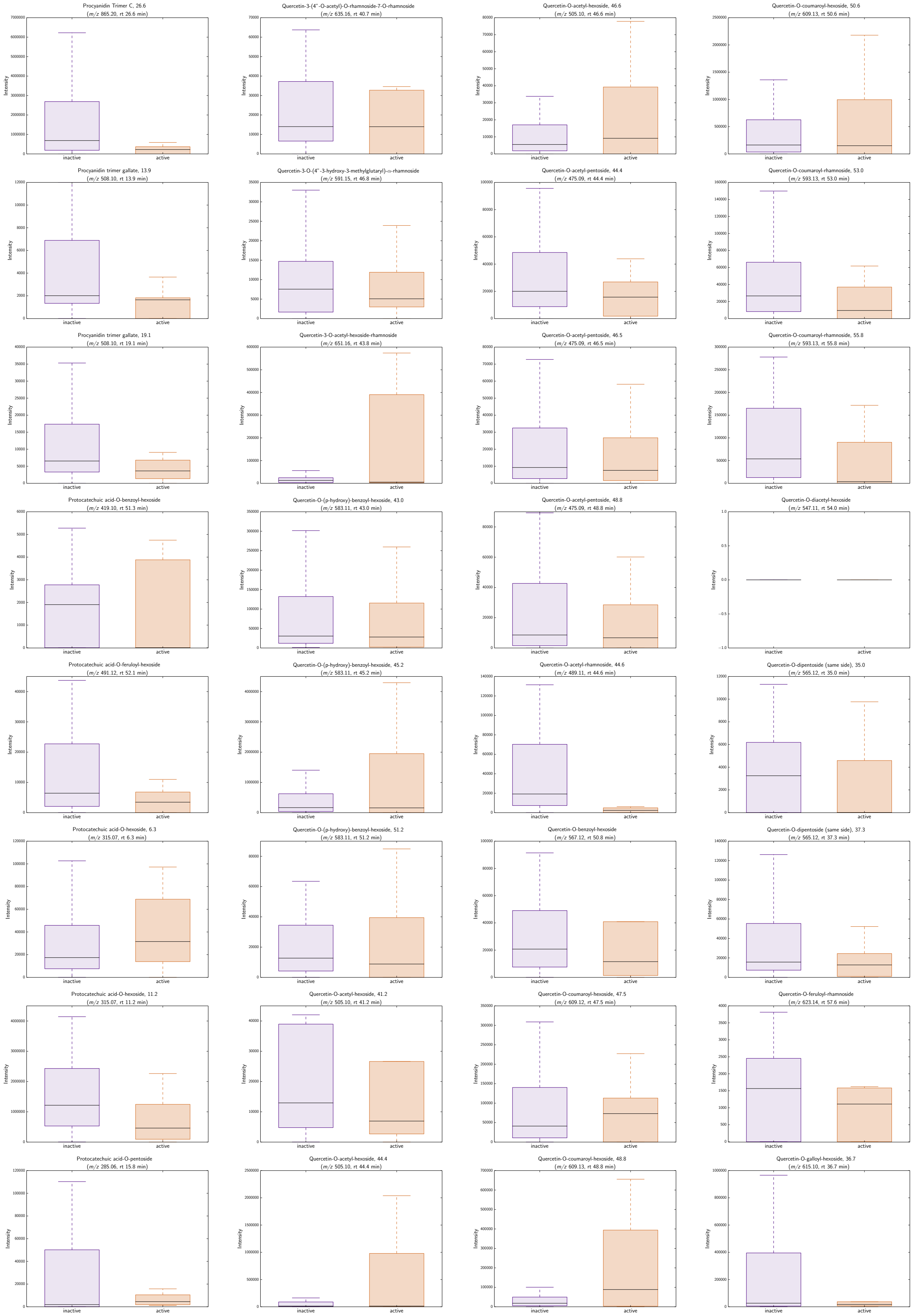
Procyanidin Trimer C, 26.6 (m/z 865.20, rt 26.6 min)
Quercetin-3-(4''-O-acetyl)-O-rhamnoside-7-O-rhamnoside (m/z 635.16, rt 40.7 min)
Quercetin-O-acetyl-hexoside, 46.6 (m/z 505.10, rt 46.6 min)
Quercetin-O-coumaroyl-hexoside, 50.6 (m/z 609.13, rt 50.6 min)
Procyanidin trimer gallate, 13.9 (m/z 508.10, rt 13.9 min)
Quercetin-3-O-(4''-3-hydroxy-3-methylglutaryl)-α-rhamnoside (m/z 591.15, rt 46.8 min)
Quercetin-O-acetyl-pentoside, 44.4 (m/z 475.09, rt 44.4 min)
Quercetin-O-coumaroyl-rhamnoside, 53.0 (m/z 593.13, rt 53.0 min)
Procyanidin trimer gallate, 19.1 (m/z 508.10, rt 19.1 min)
Quercetin-3-O-acetyl-hexoside-rhamnoside (m/z 651.16, rt 43.8 min)
Quercetin-O-acetyl-pentoside, 46.5 (m/z 475.09, rt 46.5 min)
Quercetin-O-coumaroyl-rhamnoside, 55.8 (m/z 593.13, rt 55.8 min)
Protocatechuic acid-O-benzoyl-hexoside (m/z 419.10, rt 51.3 min)
Quercetin-O-(p-hydroxy)-benzoyl-hexoside, 43.0 (m/z 583.11, rt 43.0 min)
Quercetin-O-acetyl-pentoside, 48.8 (m/z 475.09, rt 48.8 min)
Quercetin-O-diacetyl-hexoside (m/z 547.11, rt 54.0 min)
Protocatechuic acid-O-feruloyl-hexoside (m/z 491.12, rt 52.1 min)
Quercetin-O-(p-hydroxy)-benzoyl-hexoside, 45.2 (m/z 583.11, rt 45.2 min)
Quercetin-O-acetyl-rhamnoside, 44.6 (m/z 489.11, rt 44.6 min)
Quercetin-O-dipentoside (same side), 35.0 (m/z 565.12, rt 35.0 min)
Protocatechuic acid-O-hexoside, 6.3 (m/z 315.07, rt 6.3 min)
Quercetin-O-(p-hydroxy)-benzoyl-hexoside, 51.2 (m/z 583.11, rt 51.2 min)
Quercetin-O-benzoyl-hexoside (m/z 567.12, rt 50.8 min)
Quercetin-O-dipentoside (same side), 37.3 (m/z 565.12, rt 37.3 min)
Protocatechuic acid-O-hexoside, 11.2 (m/z 315.07, rt 11.2 min)
Quercetin-O-acetyl-hexoside, 41.2 (m/z 505.10, rt 41.2 min)
Quercetin-O-coumaroyl-hexoside, 47.5 (m/z 609.12, rt 47.5 min)
Quercetin-O-feruloyl-rhamnoside (m/z 623.14, rt 57.6 min)
Protocatechuic acid-O-pentoside (m/z 285.06, rt 15.8 min)
Quercetin-O-acetyl-hexoside, 44.4 (m/z 505.10, rt 44.4 min)
Quercetin-O-coumaroyl-hexoside, 48.8 (m/z 609.13, rt 48.8 min)
Quercetin-O-galloyl-hexoside, 36.7 (m/z 615.10, rt 36.7 min)
Intensity
inactive
active

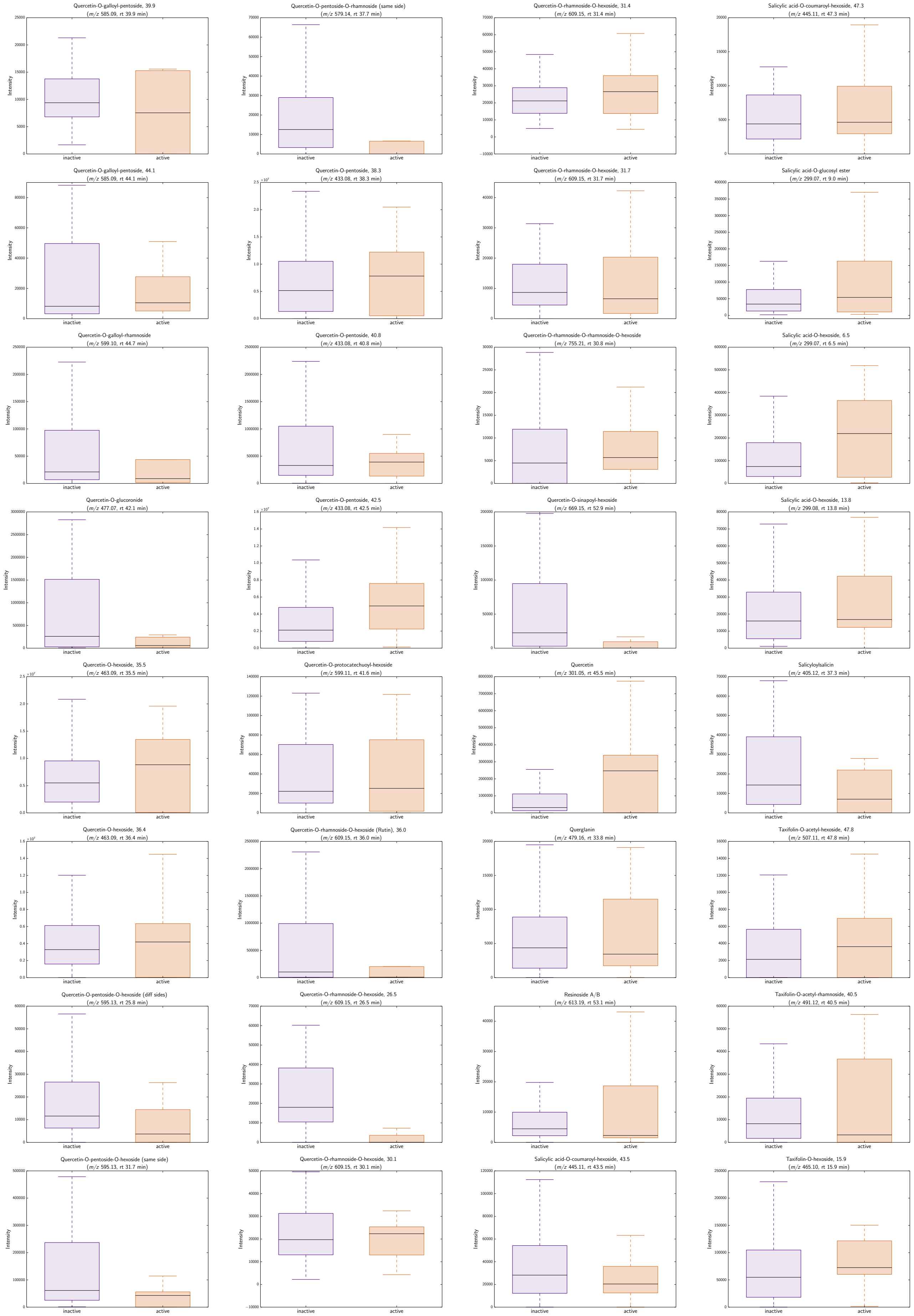
Quercetin-O-galloyl-pentoside, 39.9
(m/z 585.09, rt 39.9 min)
Quercetin-O-pentoside-O-rhamnoside (same side)
(m/z 579.14, rt 37.7 min)
Quercetin-O-rhamnoside-O-hexoside, 31.4
(m/z 609.15, rt 31.4 min)
Salicylic acid-O-coumaroyl-hexoside, 47.3
(m/z 445.11, rt 47.3 min)
Quercetin-O-galloyl-pentoside, 44.1
(m/z 585.09, rt 44.1 min)
Quercetin-O-pentoside, 38.3
(m/z 433.08, rt 38.3 min)
Quercetin-O-rhamnoside-O-hexoside, 31.7
(m/z 609.15, rt 31.7 min)
Salicylic acid-O-glucosyl ester
(m/z 299.07, rt 9.0 min)
Quercetin-O-galloyl-rhamnoside
(m/z 599.10, rt 44.7 min)
Quercetin-O-pentoside, 40.8
(m/z 433.08, rt 40.8 min)
Quercetin-O-rhamnoside-O-rhamnoside-O-hexoside
(m/z 755.21, rt 30.8 min)
Salicylic acid-O-hexoside, 6.5
(m/z 299.07, rt 6.5 min)
Quercetin-O-glucoronide
(m/z 477.07, rt 42.1 min)
Quercetin-O-pentoside, 42.5
(m/z 433.08, rt 42.5 min)
Quercetin-O-sinapoyl-hexoside
(m/z 669.15, rt 52.9 min)
Salicylic acid-O-hexoside, 13.8
(m/z 299.08, rt 13.8 min)
Quercetin-O-hexoside, 35.5
(m/z 463.09, rt 35.5 min)
Quercetin-O-protocatechuoyl-hexoside
(m/z 599.11, rt 41.6 min)
Quercetin
(m/z 301.05, rt 45.5 min)
Salicyloylsalicin
(m/z 405.12, rt 37.3 min)
Quercetin-O-hexoside, 36.4
(m/z 463.09, rt 36.4 min)
Quercetin-O-rhamnoside-O-hexoside (Rutin), 36.0
(m/z 609.15, rt 36.0 min)
Querglanin
(m/z 479.16, rt 33.8 min)
Taxifolin-O-acetyl-hexoside, 47.8
(m/z 507.11, rt 47.8 min)
Quercetin-O-pentoside-O-hexoside (diff sides)
(m/z 595.13, rt 25.8 min)
Quercetin-O-rhamnoside-O-hexoside, 26.5
(m/z 609.15, rt 26.5 min)
Resinoside A/B
(m/z 613.19, rt 53.1 min)
Taxifolin-O-acetyl-rhamnoside, 40.5
(m/z 491.12, rt 40.5 min)
Quercetin-O-pentoside-O-hexoside (same side)
(m/z 595.13, rt 31.7 min)
Quercetin-O-rhamnoside-O-hexoside, 30.1
(m/z 609.15, rt 30.1 min)
Salicylic acid-O-coumaroyl-hexoside, 43.5
(m/z 445.11, rt 43.5 min)
Taxifolin-O-hexoside, 15.9
(m/z 465.10, rt 15.9 min)
Intensity
inactive
active

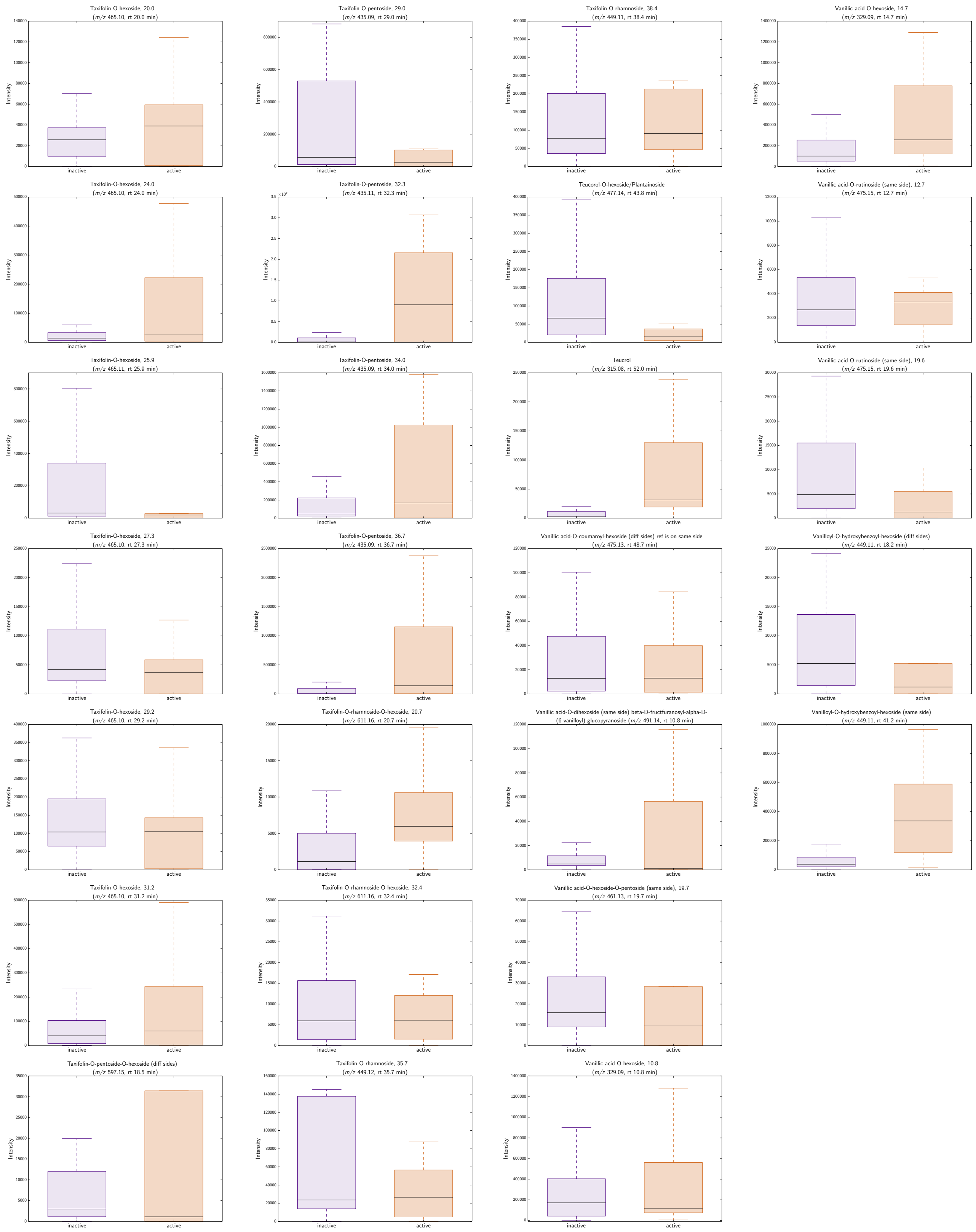

Figure S4 | Box plots of the 282 identified polyphenolics with no significant difference in LC-MS intesity with respect to antimicrobial activity classification of all 87 *Rhododendron* species. Those 10 compounds showing significant differences are given in Fig 4. 17 of the 87 *Rhododendron* species are denoted as antimicrobial active (orange), *i.e.*, the radius of the agar diffusion assay is ≥ 0.6 cm, and, thus, 70 species are characterized as antimicrobial inactive (violet).

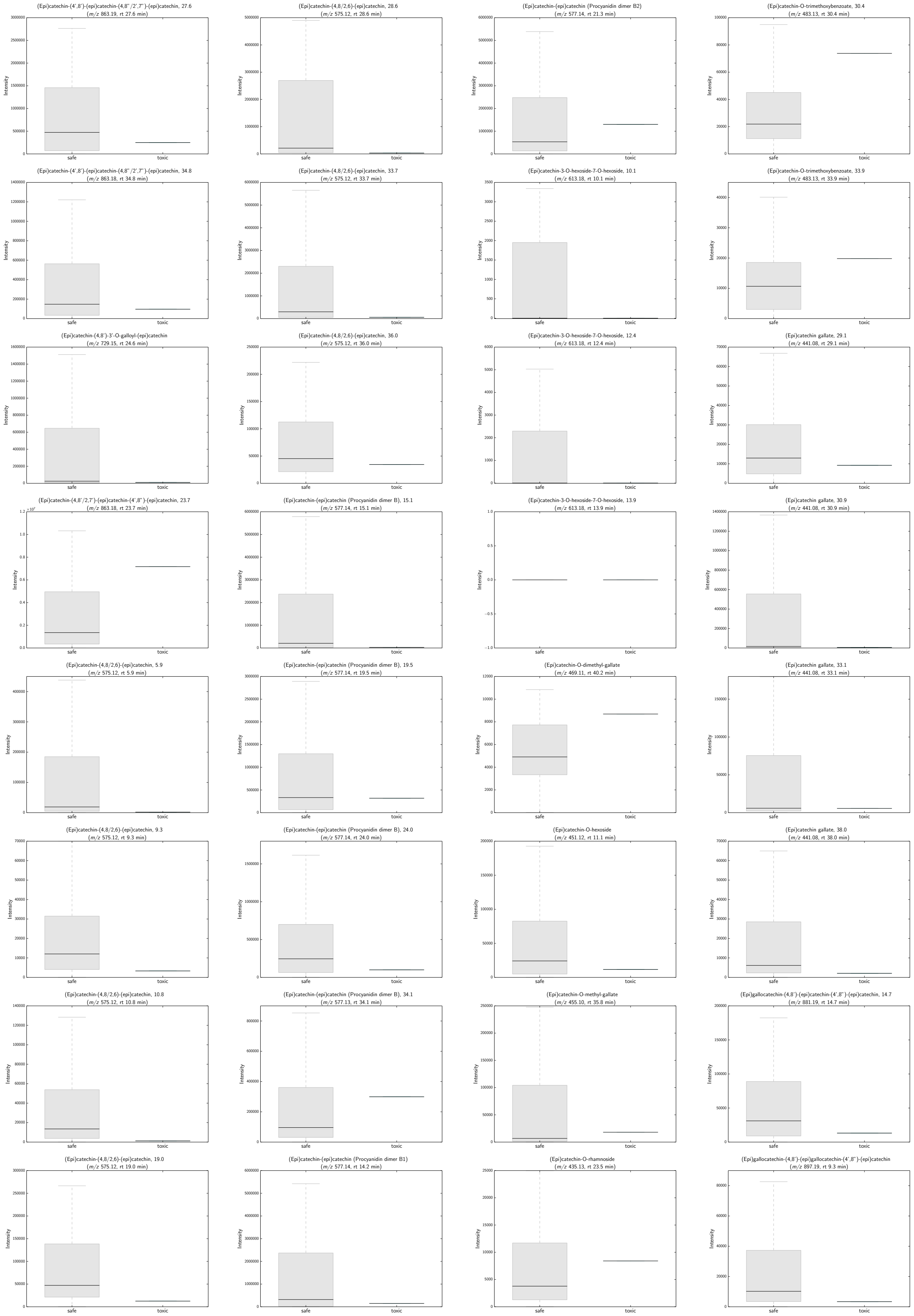
(Epi)catechin-(4',8')-(epi)catechin-(4,8''/2',7'')-(epi)catechin, 27.6
(m/z 863.19, rt 27.6 min)
(Epi)catechin-(4,8/2,6)-(epi)catechin, 28.6
(m/z 575.12, rt 28.6 min)
(Epi)catechin-(epi)catechin (Procyanidin dimer B2)
(m/z 577.14, rt 21.3 min)
(Epi)catechin-O-trimethoxybenzoate, 30.4
(m/z 483.13, rt 30.4 min)
(Epi)catechin-(4',8')-(epi)catechin-(4,8''/2',7'')-(epi)catechin, 34.8
(m/z 863.18, rt 34.8 min)
(Epi)catechin-(4,8/2,6)-(epi)catechin, 33.7
(m/z 575.12, rt 33.7 min)
(Epi)catechin-3-O-hexoside-7-O-hexoside, 10.1
(m/z 613.18, rt 10.1 min)
(Epi)catechin-O-trimethoxybenzoate, 33.9
(m/z 483.13, rt 33.9 min)
(Epi)catechin-(4,8')-3'-O-galloyl-(epi)catechin
(m/z 729.15, rt 24.6 min)
(Epi)catechin-(4,8/2,6)-(epi)catechin, 36.0
(m/z 575.12, rt 36.0 min)
(Epi)catechin-3-O-hexoside-7-O-hexoside, 12.4
(m/z 613.18, rt 12.4 min)
(Epi)catechin gallate, 29.1
(m/z 441.08, rt 29.1 min)
(Epi)catechin-(4,8'/2,7')-(epi)catechin-(4',8'')-(epi)catechin, 23.7
(m/z 863.18, rt 23.7 min)
(Epi)catechin-(epi)catechin (Procyanidin dimer B), 15.1
(m/z 577.14, rt 15.1 min)
(Epi)catechin-3-O-hexoside-7-O-hexoside, 13.9
(m/z 613.18, rt 13.9 min)
(Epi)catechin gallate, 30.9
(m/z 441.08, rt 30.9 min)
(Epi)catechin-(4,8/2,6)-(epi)catechin, 5.9
(m/z 575.12, rt 5.9 min)
(Epi)catechin-(epi)catechin (Procyanidin dimer B), 19.5
(m/z 577.14, rt 19.5 min)
(Epi)catechin-O-dimethyl-gallate
(m/z 469.11, rt 40.2 min)
(Epi)catechin gallate, 33.1
(m/z 441.08, rt 33.1 min)
(Epi)catechin-(4,8/2,6)-(epi)catechin, 9.3
(m/z 575.12, rt 9.3 min)
(Epi)catechin-(epi)catechin (Procyanidin dimer B), 24.0
(m/z 577.14, rt 24.0 min)
(Epi)catechin-O-hexoside
(m/z 451.12, rt 11.1 min)
(Epi)catechin gallate, 38.0
(m/z 441.08, rt 38.0 min)
(Epi)catechin-(4,8/2,6)-(epi)catechin, 10.8
(m/z 575.12, rt 10.8 min)
(Epi)catechin-(epi)catechin (Procyanidin dimer B), 34.1
(m/z 577.13, rt 34.1 min)
(Epi)catechin-O-methyl-gallate
(m/z 455.10, rt 35.8 min)
(Epi)gallocatechin-(4,8')-(epi)catechin-(4',8'')-(epi)catechin, 14.7
(m/z 881.19, rt 14.7 min)
(Epi)catechin-(4,8/2,6)-(epi)catechin, 19.0
(m/z 575.12, rt 19.0 min)
(Epi)catechin-(epi)catechin (Procyanidin dimer B1)
(m/z 577.14, rt 14.2 min)
(Epi)catechin-O-rhamnoside
(m/z 435.13, rt 23.5 min)
(Epi)gallocatechin-(4,8')-(epi)gallocatechin-(4',8'')-(epi)catechin
(m/z 897.19, rt 9.3 min)
Intensity
safe
toxic

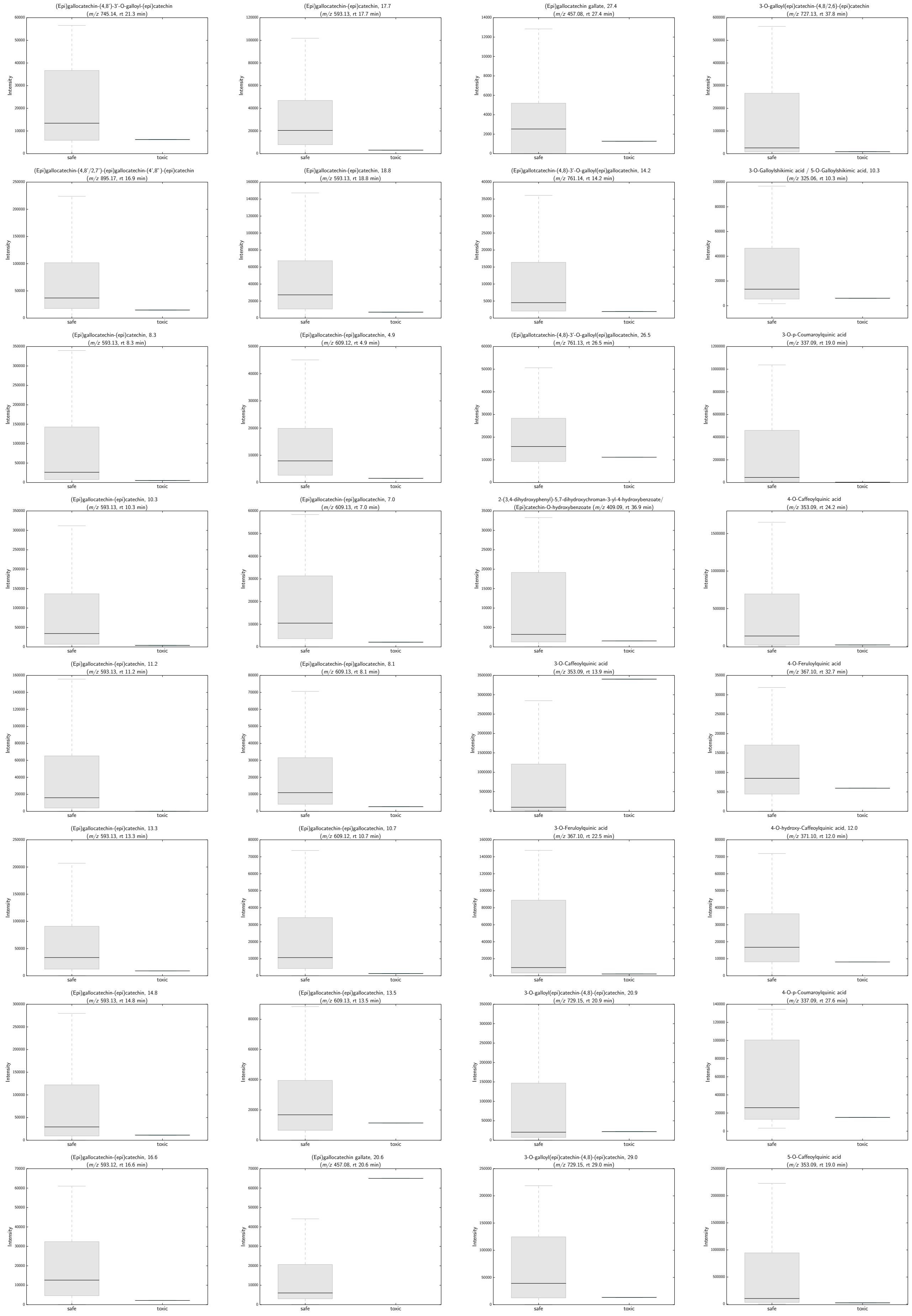
(Epi)gallocatechin-(4,8')-3'-O-galloyl-(epi)catechin
(m/z 745.14, rt 21.3 min)
(Epi)gallocatechin-(epi)catechin, 17.7
(m/z 593.13, rt 17.7 min)
(Epi)gallocatechin gallate, 27.4
(m/z 457.08, rt 27.4 min)
3-O-galloyl(epi)catechin-(4,8/2,6)-(epi)catechin
(m/z 727.13, rt 37.8 min)
(Epi)gallocatechin-(4,8'/2,7')-(epi)gallocatechin-(4',8'')-(epi)catechin
(m/z 895.17, rt 16.9 min)
(Epi)gallocatechin-(epi)catechin, 18.8
(m/z 593.13, rt 18.8 min)
(Epi)gallotcatechin-(4,8)-3'-O-galloyl(epi)gallocatechin, 14.2
(m/z 761.14, rt 14.2 min)
3-O-Galloylshikimic acid / 5-O-Galloylshikimic acid, 10.3
(m/z 325.06, rt 10.3 min)
(Epi)gallocatechin-(epi)catechin, 8.3
(m/z 593.13, rt 8.3 min)
(Epi)gallocatechin-(epi)gallocatechin, 4.9
(m/z 609.12, rt 4.9 min)
(Epi)gallotcatechin-(4,8)-3'-O-galloyl(epi)gallocatechin, 26.5
(m/z 761.13, rt 26.5 min)
3-O-p-Coumaroylquinic acid
(m/z 337.09, rt 19.0 min)
(Epi)gallocatechin-(epi)catechin, 10.3
(m/z 593.13, rt 10.3 min)
(Epi)gallocatechin-(epi)gallocatechin, 7.0
(m/z 609.13, rt 7.0 min)
2-(3,4-dihydroxyphenyl)-5,7-dihydroxychroman-3-yl-4-hydroxybenzoate/
(Epi)catechin-O-hydroxybenzoate (m/z 409.09, rt 36.9 min)
4-O-Caffeoylquinic acid
(m/z 353.09, rt 24.2 min)
(Epi)gallocatechin-(epi)catechin, 11.2
(m/z 593.13, rt 11.2 min)
(Epi)gallocatechin-(epi)gallocatechin, 8.1
(m/z 609.13, rt 8.1 min)
3-O-Caffeoylquinic acid
(m/z 353.09, rt 13.9 min)
4-O-Feruloylquinic acid
(m/z 367.10, rt 32.7 min)
(Epi)gallocatechin-(epi)catechin, 13.3
(m/z 593.13, rt 13.3 min)
(Epi)gallocatechin-(epi)gallocatechin, 10.7
(m/z 609.12, rt 10.7 min)
3-O-Feruloylquinic acid
(m/z 367.10, rt 22.5 min)
4-O-hydroxy-Caffeoylquinic acid, 12.0
(m/z 371.10, rt 12.0 min)
(Epi)gallocatechin-(epi)catechin, 14.8
(m/z 593.13, rt 14.8 min)
(Epi)gallocatechin-(epi)gallocatechin, 13.5
(m/z 609.13, rt 13.5 min)
3-O-galloyl(epi)catechin-(4,8)-(epi)catechin, 20.9
(m/z 729.15, rt 20.9 min)
4-O-p-Coumaroylquinic acid
(m/z 337.09, rt 27.6 min)
(Epi)gallocatechin-(epi)catechin, 16.6
(m/z 593.12, rt 16.6 min)
(Epi)gallocatechin gallate, 20.6
(m/z 457.08, rt 20.6 min)
3-O-galloyl(epi)catechin-(4,8)-(epi)catechin, 29.0
(m/z 729.15, rt 29.0 min)
5-O-Caffeoylquinic acid
(m/z 353.09, rt 19.0 min)
Intensity
safe
toxic

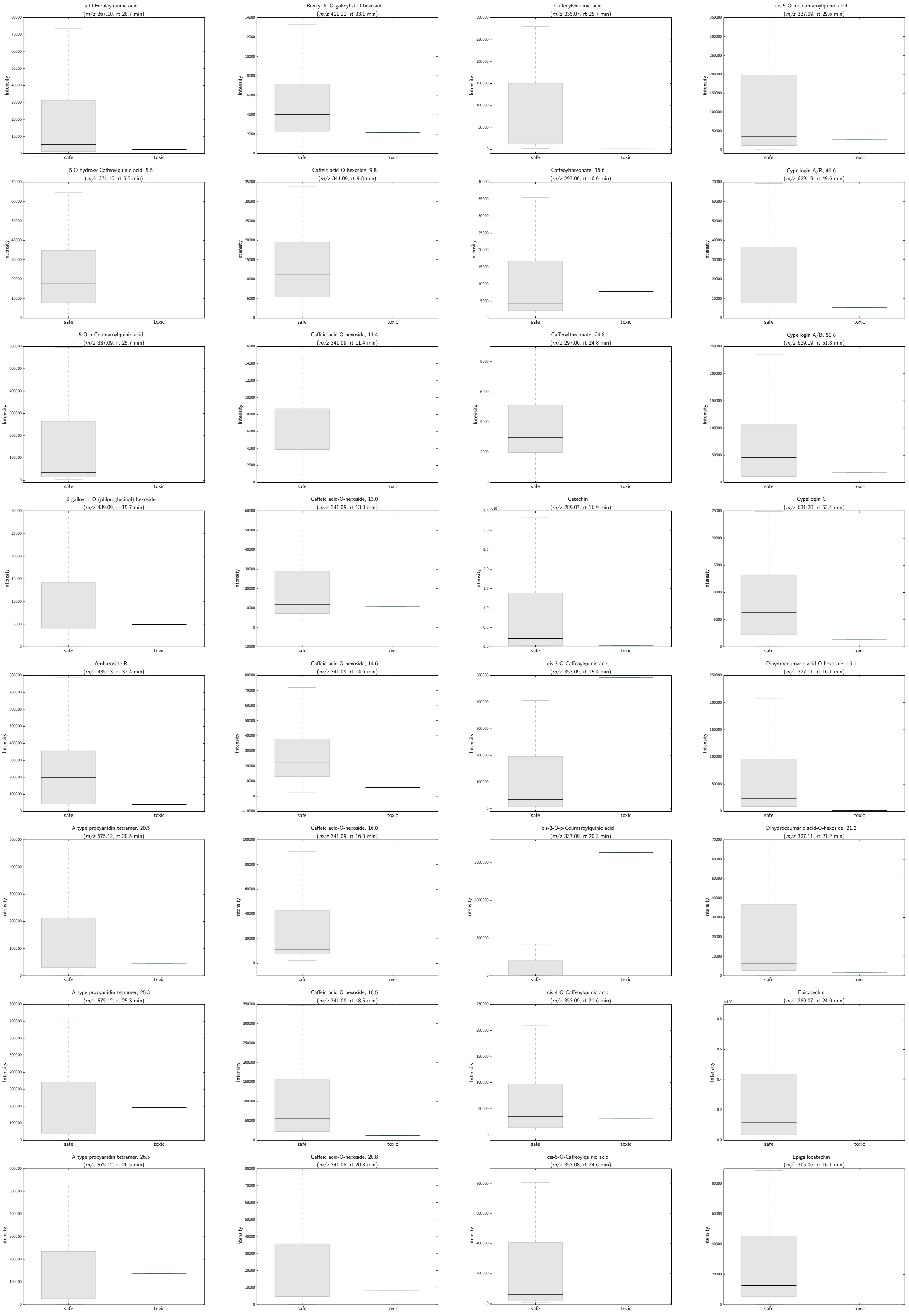
5-O-Feruloylquinic acid
(m/z 367.10, rt 28.7 min)
Benzyl-6'-O-galloyl-β-D-hexoside
(m/z 421.11, rt 33.1 min)
Caffeoylshikimic acid
(m/z 335.07, rt 25.7 min)
cis-5-O-p-Coumaroylquinic acid
(m/z 337.09, rt 29.6 min)
5-O-hydroxy-Caffeoylquinic acid, 5.5
(m/z 371.10, rt 5.5 min)
Caffeic acid-O-hexoside, 9.8
(m/z 341.09, rt 9.8 min)
Caffeoylthreonate, 16.6
(m/z 297.06, rt 16.6 min)
Cypellogin A/B, 49.6
(m/z 629.19, rt 49.6 min)
5-O-p-Coumaroylquinic acid
(m/z 337.09, rt 25.7 min)
Caffeic acid-O-hexoside, 11.4
(m/z 341.09, rt 11.4 min)
Caffeoylthreonate, 24.8
(m/z 297.06, rt 24.8 min)
Cypellogin A/B, 51.8
(m/z 629.19, rt 51.8 min)
6-galloyl-1-O-(phloroglucinol)-hexoside
(m/z 439.09, rt 15.7 min)
Caffeic acid-O-hexoside, 13.0
(m/z 341.09, rt 13.0 min)
Catechin
(m/z 289.07, rt 16.9 min)
Cypellogin C
(m/z 631.20, rt 53.4 min)
Amburoside B
(m/z 435.13, rt 37.4 min)
Caffeic acid-O-hexoside, 14.6
(m/z 341.09, rt 14.6 min)
cis-3-O-Caffeoylquinic acid
(m/z 353.09, rt 15.4 min)
Dihydrocoumaric acid-O-hexoside, 16.1
(m/z 327.11, rt 16.1 min)
A type procyanidin tetramer, 20.5
(m/z 575.12, rt 20.5 min)
Caffeic acid-O-hexoside, 16.0
(m/z 341.09, rt 16.0 min)
cis-3-O-p-Coumaroylquinic acid
(m/z 337.09, rt 20.3 min)
Dihydrocoumaric acid-O-hexoside, 21.2
(m/z 327.11, rt 21.2 min)
A type procyanidin tetramer, 25.3
(m/z 575.12, rt 25.3 min)
Caffeic acid-O-hexoside, 18.5
(m/z 341.09, rt 18.5 min)
cis-4-O-Caffeoylquinic acid
(m/z 353.09, rt 21.6 min)
Epicatechin
(m/z 289.07, rt 24.0 min)
A type procyanidin tetramer, 26.5
(m/z 575.12, rt 26.5 min)
Caffeic acid-O-hexoside, 20.8
(m/z 341.08, rt 20.8 min)
cis-5-O-Caffeoylquinic acid
(m/z 353.08, rt 24.6 min)
Epigallocatechin
(m/z 305.06, rt 16.1 min)
Intensity
safe
toxic

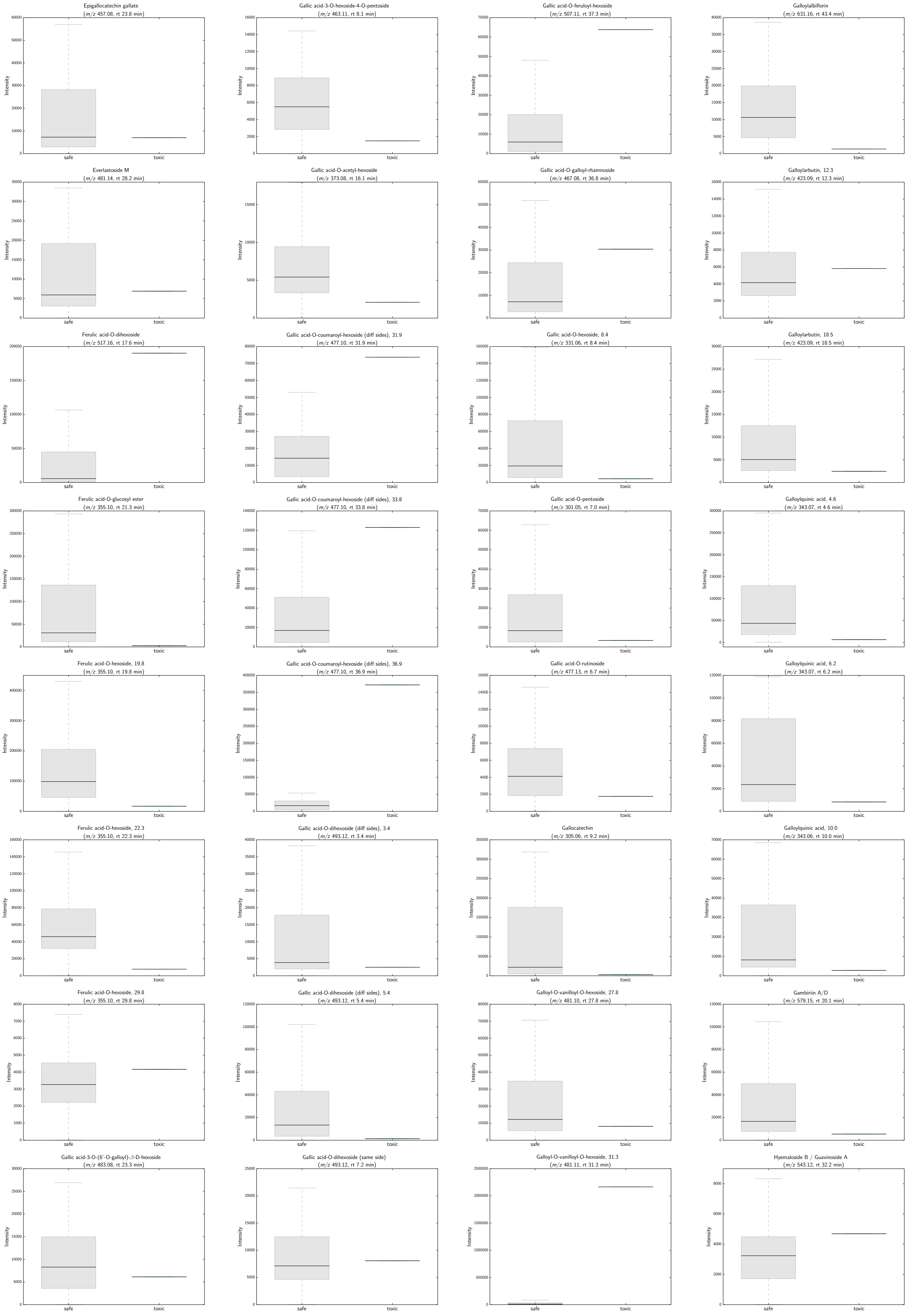
Epigallocatechin gallate
(m/z 457.08, rt 23.8 min)
Gallic acid-3-O-hexoside-4-O-pentoside
(m/z 463.11, rt 8.1 min)
Gallic acid-O-feruloyl-hexoside
(m/z 507.11, rt 37.3 min)
Galloylalbiflorin
(m/z 631.16, rt 43.4 min)
Everlastoside M
(m/z 481.14, rt 28.2 min)
Gallic acid-O-acetyl-hexoside
(m/z 373.08, rt 16.1 min)
Gallic acid-O-galloyl-rhamnoside
(m/z 467.08, rt 36.8 min)
Galloylarbutin, 12.3
(m/z 423.09, rt 12.3 min)
Ferulic acid-O-dihexoside
(m/z 517.16, rt 17.6 min)
Gallic acid-O-coumaroyl-hexoside (diff sides), 31.9
(m/z 477.10, rt 31.9 min)
Gallic acid-O-hexoside, 8.4
(m/z 331.06, rt 8.4 min)
Galloylarbutin, 18.5
(m/z 423.09, rt 18.5 min)
Ferulic acid-O-glucosyl ester
(m/z 355.10, rt 21.3 min)
Gallic acid-O-coumaroyl-hexoside (diff sides), 33.8
(m/z 477.10, rt 33.8 min)
Gallic acid-O-pentoside
(m/z 301.05, rt 7.0 min)
Galloylquinic acid, 4.6
(m/z 343.07, rt 4.6 min)
Ferulic acid-O-hexoside, 19.8
(m/z 355.10, rt 19.8 min)
Gallic acid-O-coumaroyl-hexoside (diff sides), 36.9
(m/z 477.10, rt 36.9 min)
Gallic acid-O-rutinoside
(m/z 477.13, rt 6.7 min)
Galloylquinic acid, 6.2
(m/z 343.07, rt 6.2 min)
Ferulic acid-O-hexoside, 22.3
(m/z 355.10, rt 22.3 min)
Gallic acid-O-dihexoside (diff sides), 3.4
(m/z 493.12, rt 3.4 min)
Gallocatechin
(m/z 305.06, rt 9.2 min)
Galloylquinic acid, 10.0
(m/z 343.06, rt 10.0 min)
Ferulic acid-O-hexoside, 29.8
(m/z 355.10, rt 29.8 min)
Gallic acid-O-dihexoside (diff sides), 5.4
(m/z 493.12, rt 5.4 min)
Galloyl-O-vanilloyl-O-hexoside, 27.8
(m/z 481.10, rt 27.8 min)
Gambiriin A/D
(m/z 579.15, rt 20.1 min)
Gallic acid-3-O-(6'-O-galloyl)-β-D-hexoside
(m/z 483.08, rt 23.3 min)
Gallic acid-O-dihexoside (same side)
(m/z 493.12, rt 7.2 min)
Galloyl-O-vanilloyl-O-hexoside, 31.3
(m/z 481.11, rt 31.3 min)
Hyemaloside B / Guavinoside A
(m/z 543.12, rt 32.2 min)
Intensity
safe
toxic

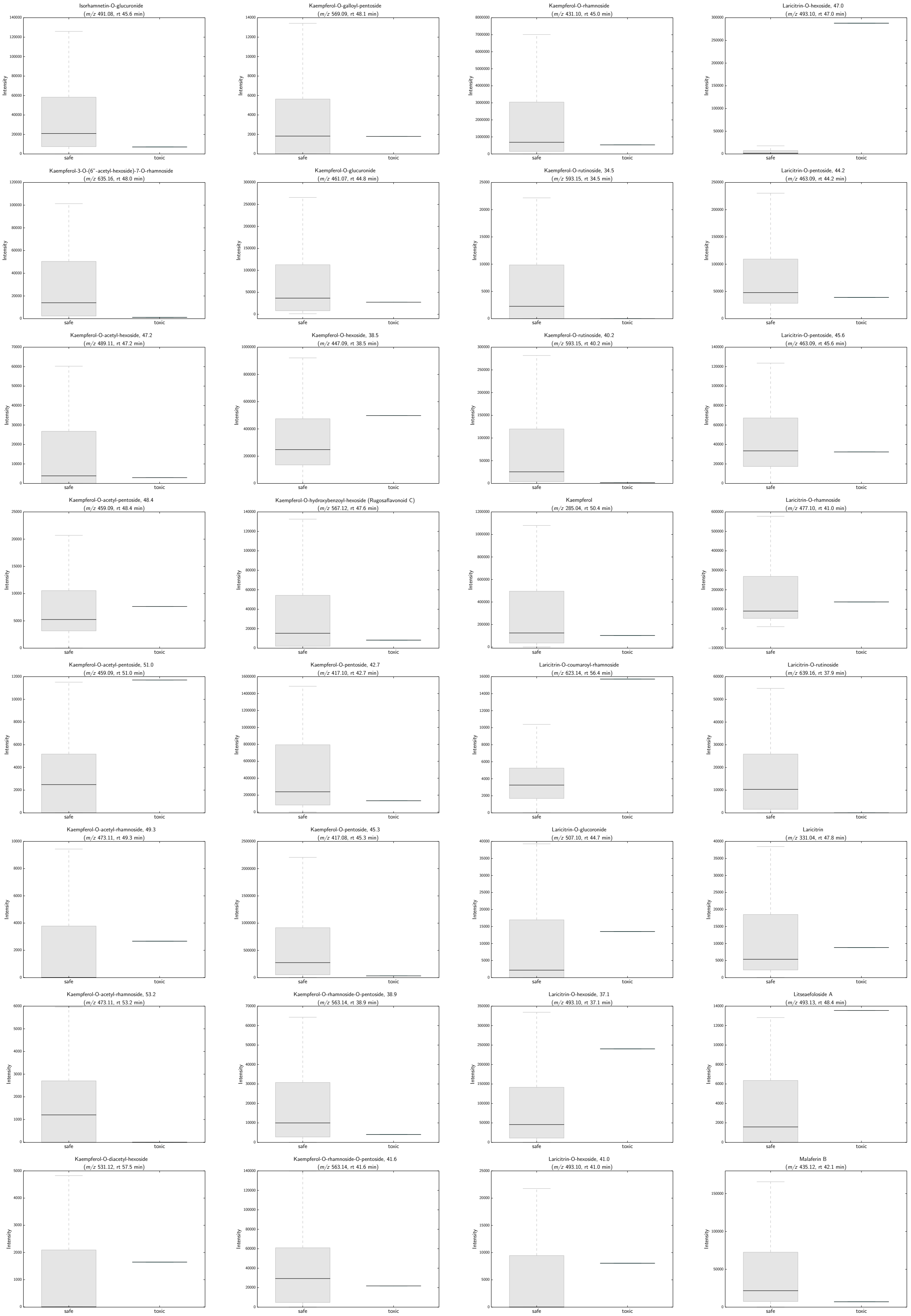
Isorhamnetin-O-glucuronide
(m/z 491.08, rt 45.6 min)
Kaempferol-O-galloyl-pentoside
(m/z 569.09, rt 48.1 min)
Kaempferol-O-rhamnoside
(m/z 431.10, rt 45.0 min)
Laricitrin-O-hexoside, 47.0
(m/z 493.10, rt 47.0 min)
Kaempferol-3-O-(6''-acetyl-hexoside)-7-O-rhamnoside
(m/z 635.16, rt 48.0 min)
Kaempferol-O-glucuronide
(m/z 461.07, rt 44.8 min)
Kaempferol-O-rutinoside, 34.5
(m/z 593.15, rt 34.5 min)
Laricitrin-O-pentoside, 44.2
(m/z 463.09, rt 44.2 min)
Kaempferol-O-acetyl-hexoside, 47.2
(m/z 489.11, rt 47.2 min)
Kaempferol-O-hexoside, 38.5
(m/z 447.09, rt 38.5 min)
Kaempferol-O-rutinoside, 40.2
(m/z 593.15, rt 40.2 min)
Laricitrin-O-pentoside, 45.6
(m/z 463.09, rt 45.6 min)
Kaempferol-O-acetyl-pentoside, 48.4
(m/z 459.09, rt 48.4 min)
Kaempferol-O-hydroxybenzoyl-hexoside (Rugosaflavonoid C)
(m/z 567.12, rt 47.6 min)
Kaempferol
(m/z 285.04, rt 50.4 min)
Laricitrin-O-rhamnoside
(m/z 477.10, rt 41.0 min)
Kaempferol-O-acetyl-pentoside, 51.0
(m/z 459.09, rt 51.0 min)
Kaempferol-O-pentoside, 42.7
(m/z 417.10, rt 42.7 min)
Laricitrin-O-coumaroyl-rhamnoside
(m/z 623.14, rt 56.4 min)
Laricitrin-O-rutinoside
(m/z 639.16, rt 37.9 min)
Kaempferol-O-acetyl-rhamnoside, 49.3
(m/z 473.11, rt 49.3 min)
Kaempferol-O-pentoside, 45.3
(m/z 417.08, rt 45.3 min)
Laricitrin-O-glucoronide
(m/z 507.10, rt 44.7 min)
Laricitrin
(m/z 331.04, rt 47.8 min)
Kaempferol-O-acetyl-rhamnoside, 53.2
(m/z 473.11, rt 53.2 min)
Kaempferol-O-rhamnoside-O-pentoside, 38.9
(m/z 563.14, rt 38.9 min)
Laricitrin-O-hexoside, 37.1
(m/z 493.10, rt 37.1 min)
Litseaefoloside A
(m/z 493.13, rt 48.4 min)
Kaempferol-O-diacetyl-hexoside
(m/z 531.12, rt 57.5 min)
Kaempferol-O-rhamnoside-O-pentoside, 41.6
(m/z 563.14, rt 41.6 min)
Laricitrin-O-hexoside, 41.0
(m/z 493.10, rt 41.0 min)
Malaferin B
(m/z 435.12, rt 42.1 min)
Intensity
safe
toxic

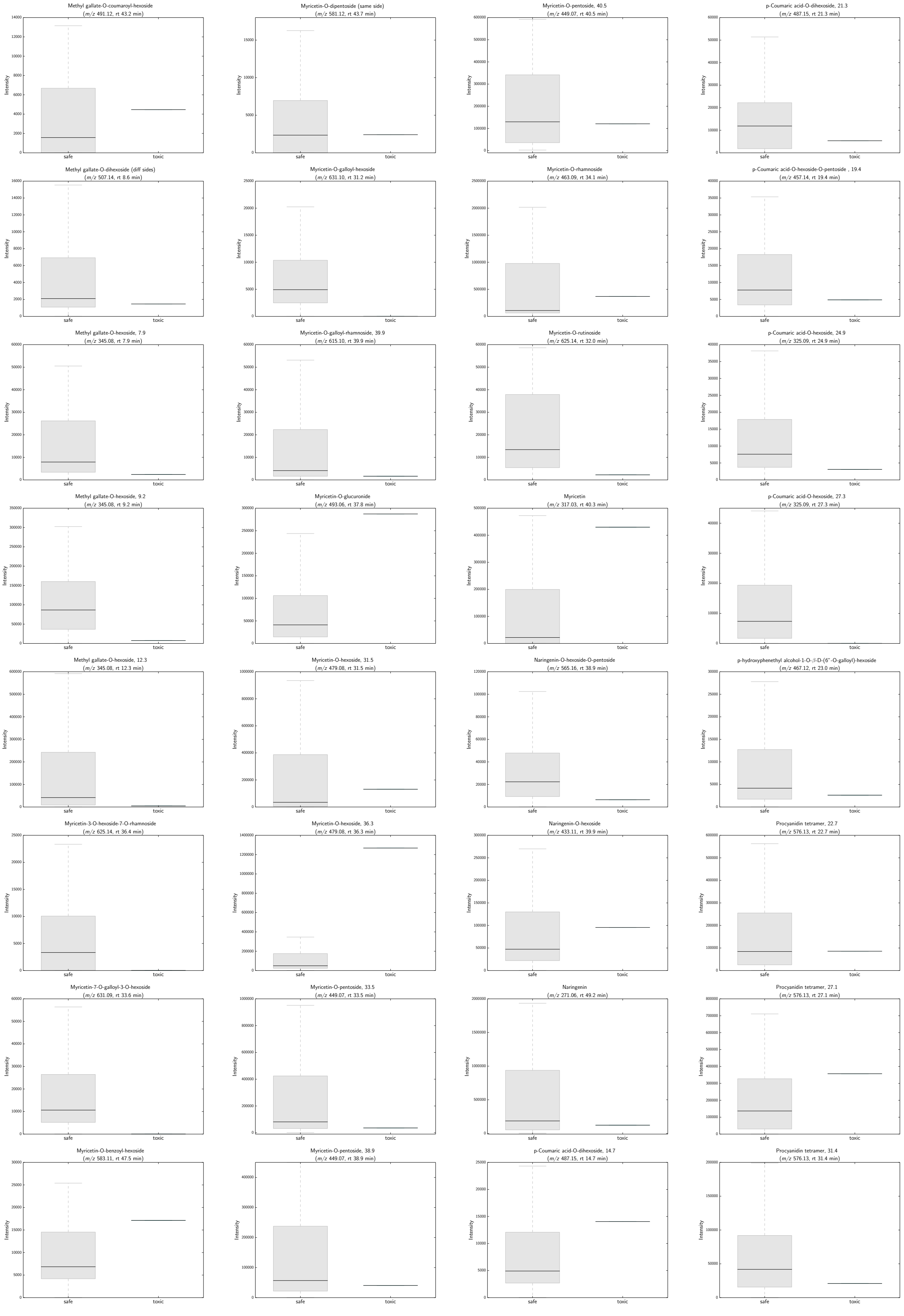
Methyl gallate-O-coumaroyl-hexoside (m/z 491.12, rt 43.2 min)
Myricetin-O-dipentoside (same side) (m/z 581.12, rt 43.7 min)
Myricetin-O-pentoside, 40.5 (m/z 449.07, rt 40.5 min)
p-Coumaric acid-O-dihexoside, 21.3 (m/z 487.15, rt 21.3 min)
Methyl gallate-O-dihexoside (diff sides) (m/z 507.14, rt 8.6 min)
Myricetin-O-galloyl-hexoside (m/z 631.10, rt 31.2 min)
Myricetin-O-rhamnoside (m/z 463.09, rt 34.1 min)
p-Coumaric acid-O-hexoside-O-pentoside , 19.4 (m/z 457.14, rt 19.4 min)
Methyl gallate-O-hexoside, 7.9 (m/z 345.08, rt 7.9 min)
Myricetin-O-galloyl-rhamnoside, 39.9 (m/z 615.10, rt 39.9 min)
Myricetin-O-rutinoside (m/z 625.14, rt 32.0 min)
p-Coumaric acid-O-hexoside, 24.9 (m/z 325.09, rt 24.9 min)
Methyl gallate-O-hexoside, 9.2 (m/z 345.08, rt 9.2 min)
Myricetin-O-glucuronide (m/z 493.06, rt 37.8 min)
Myricetin (m/z 317.03, rt 40.3 min)
p-Coumaric acid-O-hexoside, 27.3 (m/z 325.09, rt 27.3 min)
Methyl gallate-O-hexoside, 12.3 (m/z 345.08, rt 12.3 min)
Myricetin-O-hexoside, 31.5 (m/z 479.08, rt 31.5 min)
Naringenin-O-hexoside-O-pentoside (m/z 565.16, rt 38.9 min)
p-hydroxyphenethyl alcohol-1-O-β-D-(6''-O-galloyl)-hexoside (m/z 467.12, rt 23.0 min)
Myricetin-3-O-hexoside-7-O-rhamnoside (m/z 625.14, rt 36.4 min)
Myricetin-O-hexoside, 36.3 (m/z 479.08, rt 36.3 min)
Naringenin-O-hexoside (m/z 433.11, rt 39.9 min)
Procyanidin tetramer, 22.7 (m/z 576.13, rt 22.7 min)
Myricetin-7-O-galloyl-3-O-hexoside (m/z 631.09, rt 33.6 min)
Myricetin-O-pentoside, 33.5 (m/z 449.07, rt 33.5 min)
Naringenin (m/z 271.06, rt 49.2 min)
Procyanidin tetramer, 27.1 (m/z 576.13, rt 27.1 min)
Myricetin-O-benzoyl-hexoside (m/z 583.11, rt 47.5 min)
Myricetin-O-pentoside, 38.9 (m/z 449.07, rt 38.9 min)
p-Coumaric acid-O-dihexoside, 14.7 (m/z 487.15, rt 14.7 min)
Procyanidin tetramer, 31.4 (m/z 576.13, rt 31.4 min)
Intensity
safe
toxic

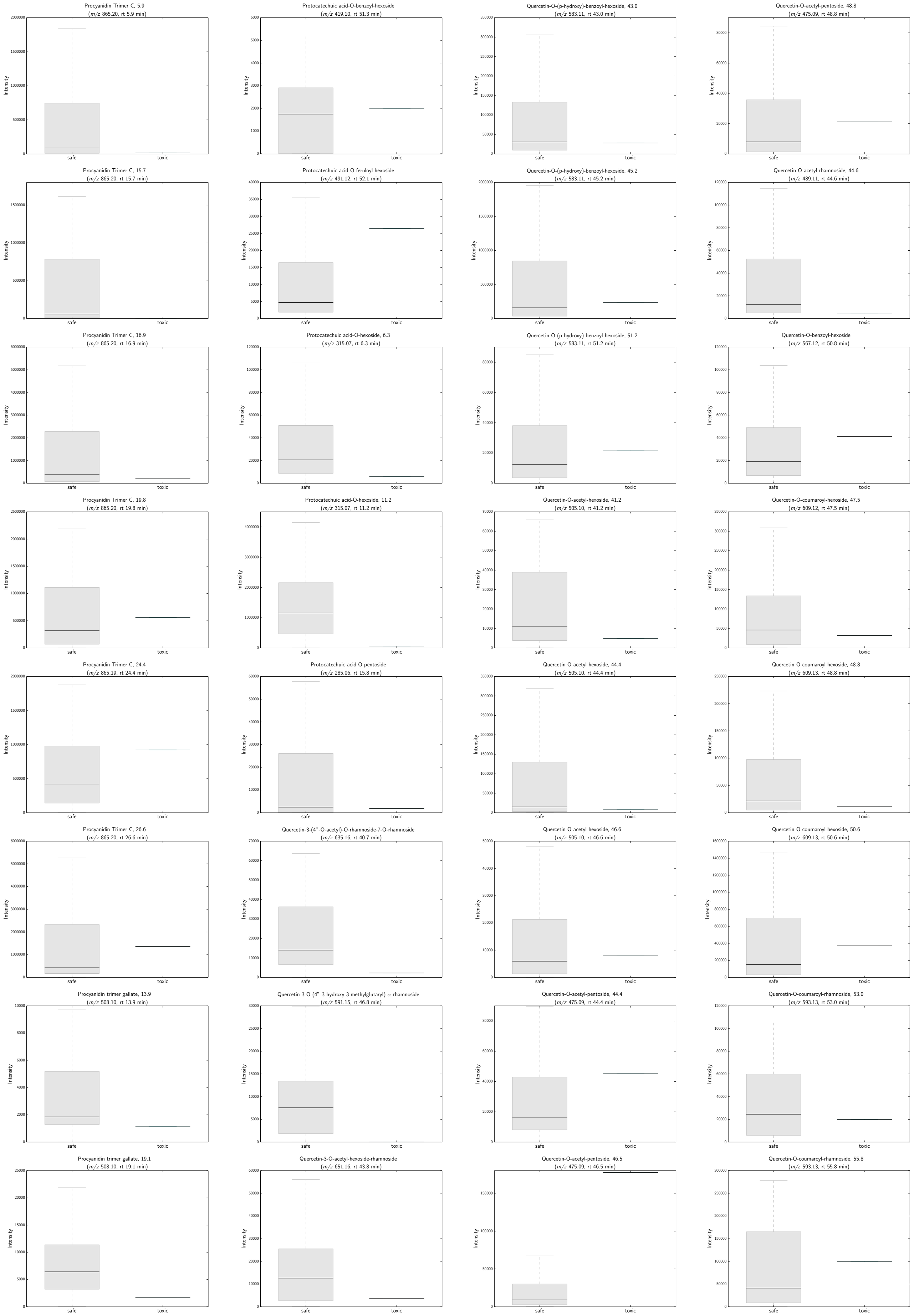
Procyanidin Trimer C, 5.9
(m/z 865.20, rt 5.9 min)
Protocatechuic acid-O-benzoyl-hexoside
(m/z 419.10, rt 51.3 min)
Quercetin-O-(p-hydroxy)-benzoyl-hexoside, 43.0
(m/z 583.11, rt 43.0 min)
Quercetin-O-acetyl-pentoside, 48.8
(m/z 475.09, rt 48.8 min)
Procyanidin Trimer C, 15.7
(m/z 865.20, rt 15.7 min)
Protocatechuic acid-O-feruloyl-hexoside
(m/z 491.12, rt 52.1 min)
Quercetin-O-(p-hydroxy)-benzoyl-hexoside, 45.2
(m/z 583.11, rt 45.2 min)
Quercetin-O-acetyl-rhamnoside, 44.6
(m/z 489.11, rt 44.6 min)
Procyanidin Trimer C, 16.9
(m/z 865.20, rt 16.9 min)
Protocatechuic acid-O-hexoside, 6.3
(m/z 315.07, rt 6.3 min)
Quercetin-O-(p-hydroxy)-benzoyl-hexoside, 51.2
(m/z 583.11, rt 51.2 min)
Quercetin-O-benzoyl-hexoside
(m/z 567.12, rt 50.8 min)
Procyanidin Trimer C, 19.8
(m/z 865.20, rt 19.8 min)
Protocatechuic acid-O-hexoside, 11.2
(m/z 315.07, rt 11.2 min)
Quercetin-O-acetyl-hexoside, 41.2
(m/z 505.10, rt 41.2 min)
Quercetin-O-coumaroyl-hexoside, 47.5
(m/z 609.12, rt 47.5 min)
Procyanidin Trimer C, 24.4
(m/z 865.19, rt 24.4 min)
Protocatechuic acid-O-pentoside
(m/z 285.06, rt 15.8 min)
Quercetin-O-acetyl-hexoside, 44.4
(m/z 505.10, rt 44.4 min)
Quercetin-O-coumaroyl-hexoside, 48.8
(m/z 609.13, rt 48.8 min)
Procyanidin Trimer C, 26.6
(m/z 865.20, rt 26.6 min)
Quercetin-3-(4''-O-acetyl)-O-rhamnoside-7-O-rhamnoside
(m/z 635.16, rt 40.7 min)
Quercetin-O-acetyl-hexoside, 46.6
(m/z 505.10, rt 46.6 min)
Quercetin-O-coumaroyl-hexoside, 50.6
(m/z 609.13, rt 50.6 min)
Procyanidin trimer gallate, 13.9
(m/z 508.10, rt 13.9 min)
Quercetin-3-O-(4''-3-hydroxy-3-methylglutaryl)-α-rhamnoside
(m/z 591.15, rt 46.8 min)
Quercetin-O-acetyl-pentoside, 44.4
(m/z 475.09, rt 44.4 min)
Quercetin-O-coumaroyl-rhamnoside, 53.0
(m/z 593.13, rt 53.0 min)
Procyanidin trimer gallate, 19.1
(m/z 508.10, rt 19.1 min)
Quercetin-3-O-acetyl-hexoside-rhamnoside
(m/z 651.16, rt 43.8 min)
Quercetin-O-acetyl-pentoside, 46.5
(m/z 475.09, rt 46.5 min)
Quercetin-O-coumaroyl-rhamnoside, 55.8
(m/z 593.13, rt 55.8 min)
Intensity
safe
toxic

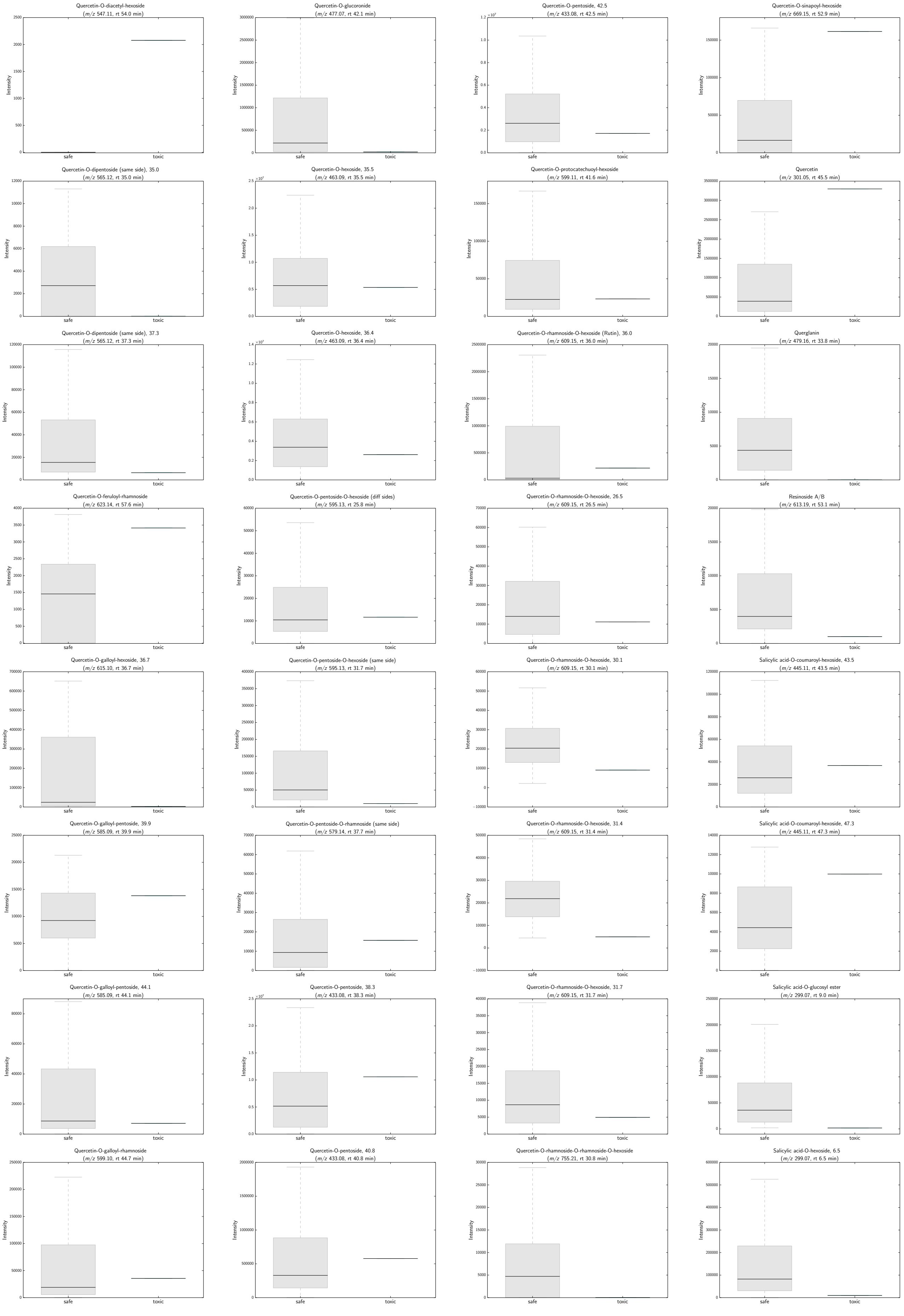
Quercetin-O-diacetyl-hexoside
(m/z 547.11, rt 54.0 min)
Quercetin-O-glucoronide
(m/z 477.07, rt 42.1 min)
Quercetin-O-pentoside, 42.5
(m/z 433.08, rt 42.5 min)
Quercetin-O-sinapoyl-hexoside
(m/z 669.15, rt 52.9 min)
Quercetin-O-dipentoside (same side), 35.0
(m/z 565.12, rt 35.0 min)
Quercetin-O-hexoside, 35.5
(m/z 463.09, rt 35.5 min)
Quercetin-O-protocatechuoyl-hexoside
(m/z 599.11, rt 41.6 min)
Quercetin
(m/z 301.05, rt 45.5 min)
Quercetin-O-dipentoside (same side), 37.3
(m/z 565.12, rt 37.3 min)
Quercetin-O-hexoside, 36.4
(m/z 463.09, rt 36.4 min)
Quercetin-O-rhamnoside-O-hexoside (Rutin), 36.0
(m/z 609.15, rt 36.0 min)
Querglanin
(m/z 479.16, rt 33.8 min)
Quercetin-O-feruloyl-rhamnoside
(m/z 623.14, rt 57.6 min)
Quercetin-O-pentoside-O-hexoside (diff sides)
(m/z 595.13, rt 25.8 min)
Quercetin-O-rhamnoside-O-hexoside, 26.5
(m/z 609.15, rt 26.5 min)
Resinoside A/B
(m/z 613.19, rt 53.1 min)
Quercetin-O-galloyl-hexoside, 36.7
(m/z 615.10, rt 36.7 min)
Quercetin-O-pentoside-O-hexoside (same side)
(m/z 595.13, rt 31.7 min)
Quercetin-O-rhamnoside-O-hexoside, 30.1
(m/z 609.15, rt 30.1 min)
Salicylic acid-O-coumaroyl-hexoside, 43.5
(m/z 445.11, rt 43.5 min)
Quercetin-O-galloyl-pentoside, 39.9
(m/z 585.09, rt 39.9 min)
Quercetin-O-pentoside-O-rhamnoside (same side)
(m/z 579.14, rt 37.7 min)
Quercetin-O-rhamnoside-O-hexoside, 31.4
(m/z 609.15, rt 31.4 min)
Salicylic acid-O-coumaroyl-hexoside, 47.3
(m/z 445.11, rt 47.3 min)
Quercetin-O-galloyl-pentoside, 44.1
(m/z 585.09, rt 44.1 min)
Quercetin-O-pentoside, 38.3
(m/z 433.08, rt 38.3 min)
Quercetin-O-rhamnoside-O-hexoside, 31.7
(m/z 609.15, rt 31.7 min)
Salicylic acid-O-glucosyl ester
(m/z 299.07, rt 9.0 min)
Quercetin-O-galloyl-rhamnoside
(m/z 599.10, rt 44.7 min)
Quercetin-O-pentoside, 40.8
(m/z 433.08, rt 40.8 min)
Quercetin-O-rhamnoside-O-rhamnoside-O-hexoside
(m/z 755.21, rt 30.8 min)
Salicylic acid-O-hexoside, 6.5
(m/z 299.07, rt 6.5 min)
Intensity
safe
toxic

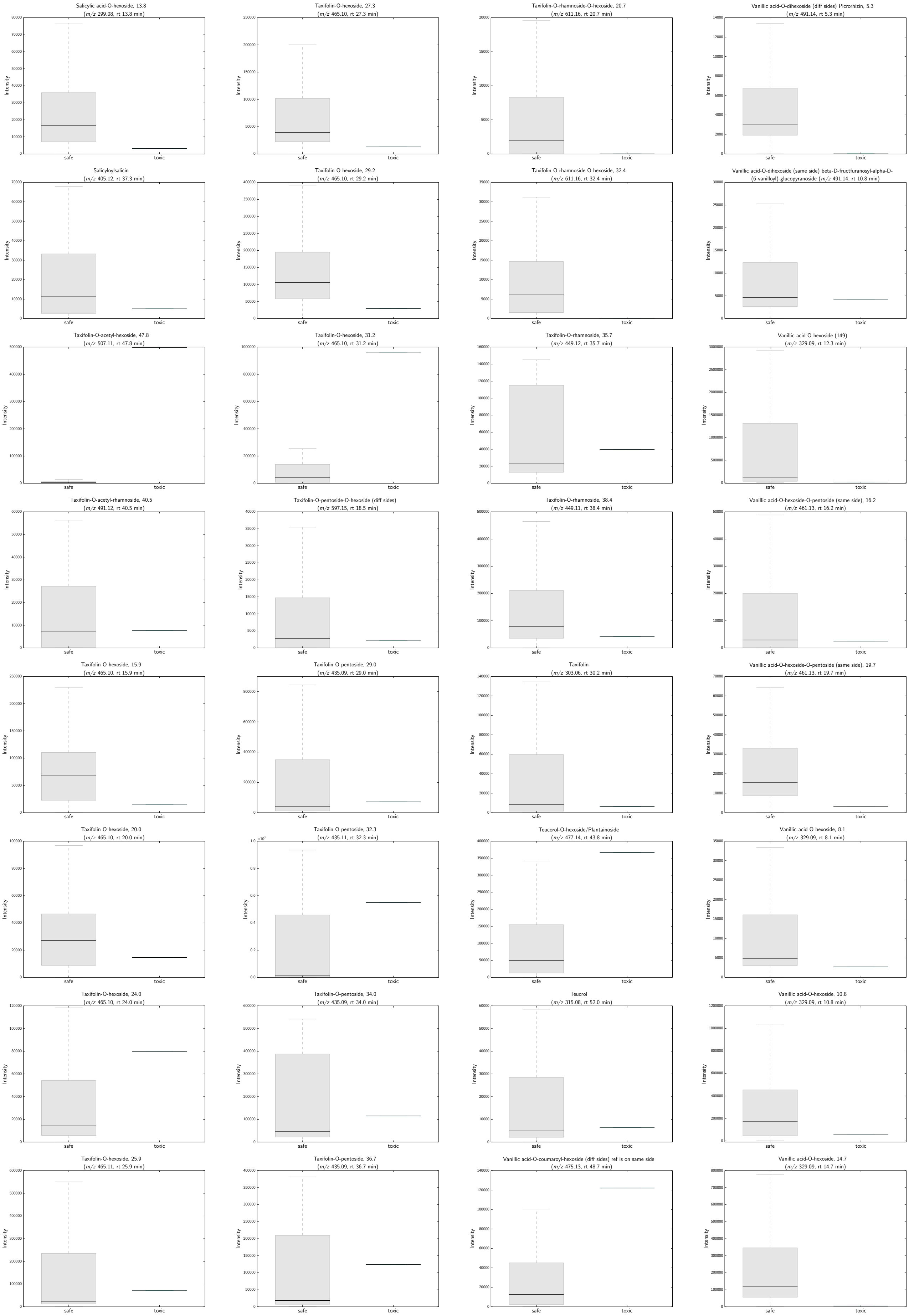
Salicylic acid-O-hexoside, 13.8
(m/z 299.08, rt 13.8 min)
Taxifolin-O-hexoside, 27.3
(m/z 465.10, rt 27.3 min)
Taxifolin-O-rhamnoside-O-hexoside, 20.7
(m/z 611.16, rt 20.7 min)
Vanillic acid-O-dihexoside (diff sides) Picrorhizin, 5.3
(m/z 491.14, rt 5.3 min)
Salicyloylsalicin
(m/z 405.12, rt 37.3 min)
Taxifolin-O-hexoside, 29.2
(m/z 465.10, rt 29.2 min)
Taxifolin-O-rhamnoside-O-hexoside, 32.4
(m/z 611.16, rt 32.4 min)
Vanillic acid-O-dihexoside (same side) beta-D-fructfuranosyl-alpha-D-
(6-vanilloyl)-glucopyranoside (m/z 491.14, rt 10.8 min)
Taxifolin-O-acetyl-hexoside, 47.8
(m/z 507.11, rt 47.8 min)
Taxifolin-O-hexoside, 31.2
(m/z 465.10, rt 31.2 min)
Taxifolin-O-rhamnoside, 35.7
(m/z 449.12, rt 35.7 min)
Vanillic acid-O-hexoside (149)
(m/z 329.09, rt 12.3 min)
Taxifolin-O-acetyl-rhamnoside, 40.5
(m/z 491.12, rt 40.5 min)
Taxifolin-O-pentoside-O-hexoside (diff sides)
(m/z 597.15, rt 18.5 min)
Taxifolin-O-rhamnoside, 38.4
(m/z 449.11, rt 38.4 min)
Vanillic acid-O-hexoside-O-pentoside (same side), 16.2
(m/z 461.13, rt 16.2 min)
Taxifolin-O-hexoside, 15.9
(m/z 465.10, rt 15.9 min)
Taxifolin-O-pentoside, 29.0
(m/z 435.09, rt 29.0 min)
Taxifolin
(m/z 303.06, rt 30.2 min)
Vanillic acid-O-hexoside-O-pentoside (same side), 19.7
(m/z 461.13, rt 19.7 min)
Taxifolin-O-hexoside, 20.0
(m/z 465.10, rt 20.0 min)
Taxifolin-O-pentoside, 32.3
(m/z 435.11, rt 32.3 min)
Teucorol-O-hexoside/Plantainoside
(m/z 477.14, rt 43.8 min)
Vanillic acid-O-hexoside, 8.1
(m/z 329.09, rt 8.1 min)
Taxifolin-O-hexoside, 24.0
(m/z 465.10, rt 24.0 min)
Taxifolin-O-pentoside, 34.0
(m/z 435.09, rt 34.0 min)
Teucrol
(m/z 315.08, rt 52.0 min)
Vanillic acid-O-hexoside, 10.8
(m/z 329.09, rt 10.8 min)
Taxifolin-O-hexoside, 25.9
(m/z 465.11, rt 25.9 min)
Taxifolin-O-pentoside, 36.7
(m/z 435.09, rt 36.7 min)
Vanillic acid-O-coumaroyl-hexoside (diff sides) ref is on same side
(m/z 475.13, rt 48.7 min)
Vanillic acid-O-hexoside, 14.7
(m/z 329.09, rt 14.7 min)
Intensity
safe
toxic

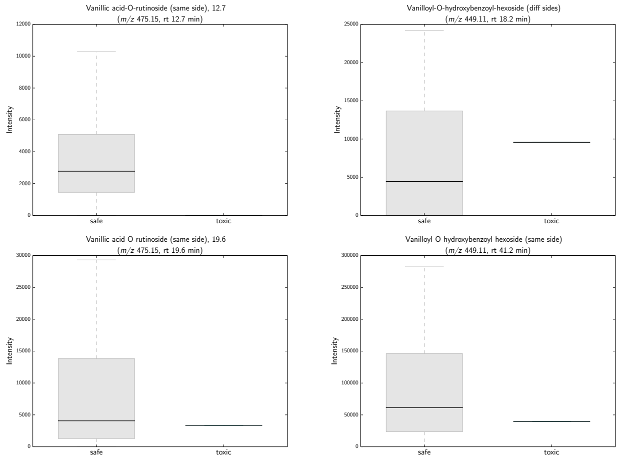

Figure S5 | Box plots of all 292 identified polyphenolics showing no significant difference in LC-MS intesity with respect to cytotoxicity classification towards HaCaT cells of all 87 *Rhododendron* species divided in cytotoxic (1 species) and non-cytotoxic (86 species, gray).

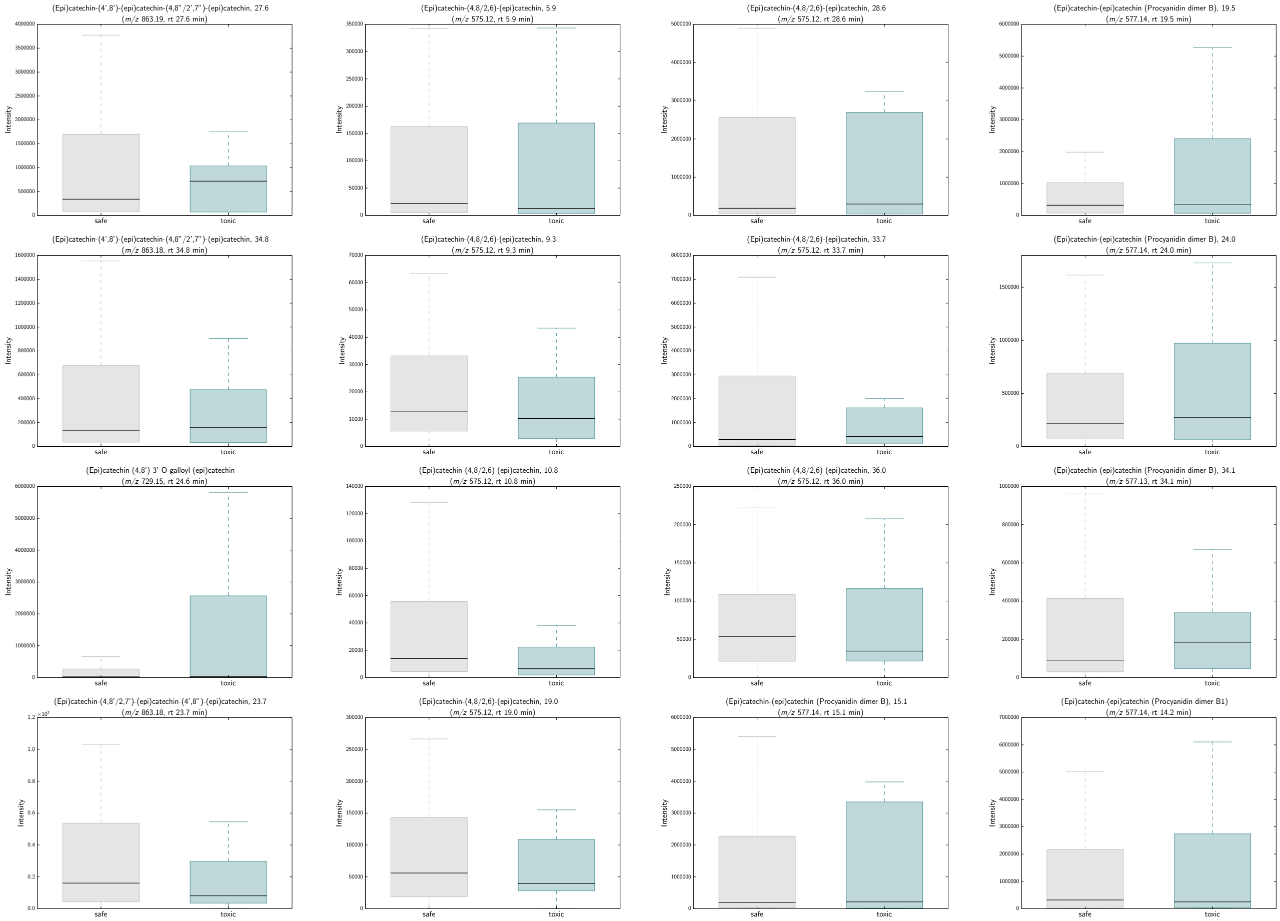

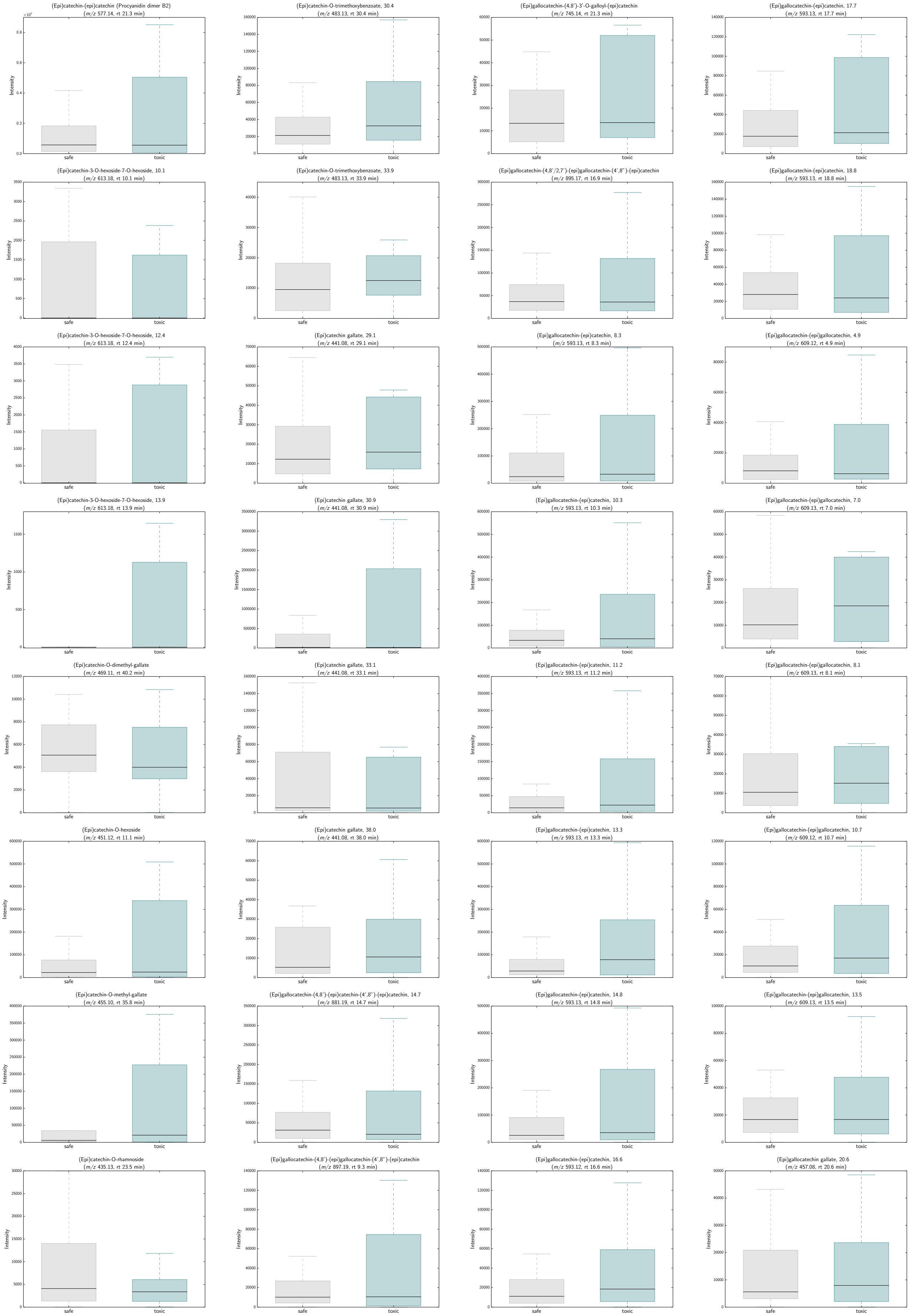
(Epi)catechin-(epi)catechin (Procyanidin dimer B2)
(m/z 577.14, rt 21.3 min)
(Epi)catechin-O-trimethoxybenzoate, 30.4
(m/z 483.13, rt 30.4 min)
(Epi)gallocatechin-(4,8')-3'-O-galloyl-(epi)catechin
(m/z 745.14, rt 21.3 min)
(Epi)gallocatechin-(epi)catechin, 17.7
(m/z 593.13, rt 17.7 min)
(Epi)catechin-3-O-hexoside-7-O-hexoside, 10.1
(m/z 613.18, rt 10.1 min)
(Epi)catechin-O-trimethoxybenzoate, 33.9
(m/z 483.13, rt 33.9 min)
(Epi)gallocatechin-(4,8'/2,7')-(epi)gallocatechin-(4',8")-(epi)catechin
(m/z 895.17, rt 16.9 min)
(Epi)gallocatechin-(epi)catechin, 18.8
(m/z 593.13, rt 18.8 min)
(Epi)catechin-3-O-hexoside-7-O-hexoside, 12.4
(m/z 613.18, rt 12.4 min)
(Epi)catechin gallate, 29.1
(m/z 441.08, rt 29.1 min)
(Epi)gallocatechin-(epi)catechin, 8.3
(m/z 593.13, rt 8.3 min)
(Epi)gallocatechin-(epi)gallocatechin, 4.9
(m/z 609.12, rt 4.9 min)
(Epi)catechin-3-O-hexoside-7-O-hexoside, 13.9
(m/z 613.18, rt 13.9 min)
(Epi)catechin gallate, 30.9
(m/z 441.08, rt 30.9 min)
(Epi)gallocatechin-(epi)catechin, 10.3
(m/z 593.13, rt 10.3 min)
(Epi)gallocatechin-(epi)gallocatechin, 7.0
(m/z 609.13, rt 7.0 min)
(Epi)catechin-O-dimethyl-gallate
(m/z 469.11, rt 40.2 min)
(Epi)catechin gallate, 33.1
(m/z 441.08, rt 33.1 min)
(Epi)gallocatechin-(epi)catechin, 11.2
(m/z 593.13, rt 11.2 min)
(Epi)gallocatechin-(epi)gallocatechin, 8.1
(m/z 609.13, rt 8.1 min)
(Epi)catechin-O-hexoside
(m/z 451.12, rt 11.1 min)
(Epi)catechin gallate, 38.0
(m/z 441.08, rt 38.0 min)
(Epi)gallocatechin-(epi)catechin, 13.3
(m/z 593.13, rt 13.3 min)
(Epi)gallocatechin-(epi)gallocatechin, 10.7
(m/z 609.12, rt 10.7 min)
(Epi)catechin-O-methyl-gallate
(m/z 455.10, rt 35.8 min)
(Epi)gallocatechin-(4,8')-(epi)catechin-(4',8")-(epi)catechin, 14.7
(m/z 881.19, rt 14.7 min)
(Epi)gallocatechin-(epi)catechin, 14.8
(m/z 593.13, rt 14.8 min)
(Epi)gallocatechin-(epi)gallocatechin, 13.5
(m/z 609.13, rt 13.5 min)
(Epi)catechin-O-rhamnoside
(m/z 435.13, rt 23.5 min)
(Epi)gallocatechin-(4,8')-(epi)gallocatechin-(4',8")-(epi)catechin
(m/z 897.19, rt 9.3 min)
(Epi)gallocatechin-(epi)catechin, 16.6
(m/z 593.12, rt 16.6 min)
(Epi)gallocatechin gallate, 20.6
(m/z 457.08, rt 20.6 min)
Intensity
safe
toxic

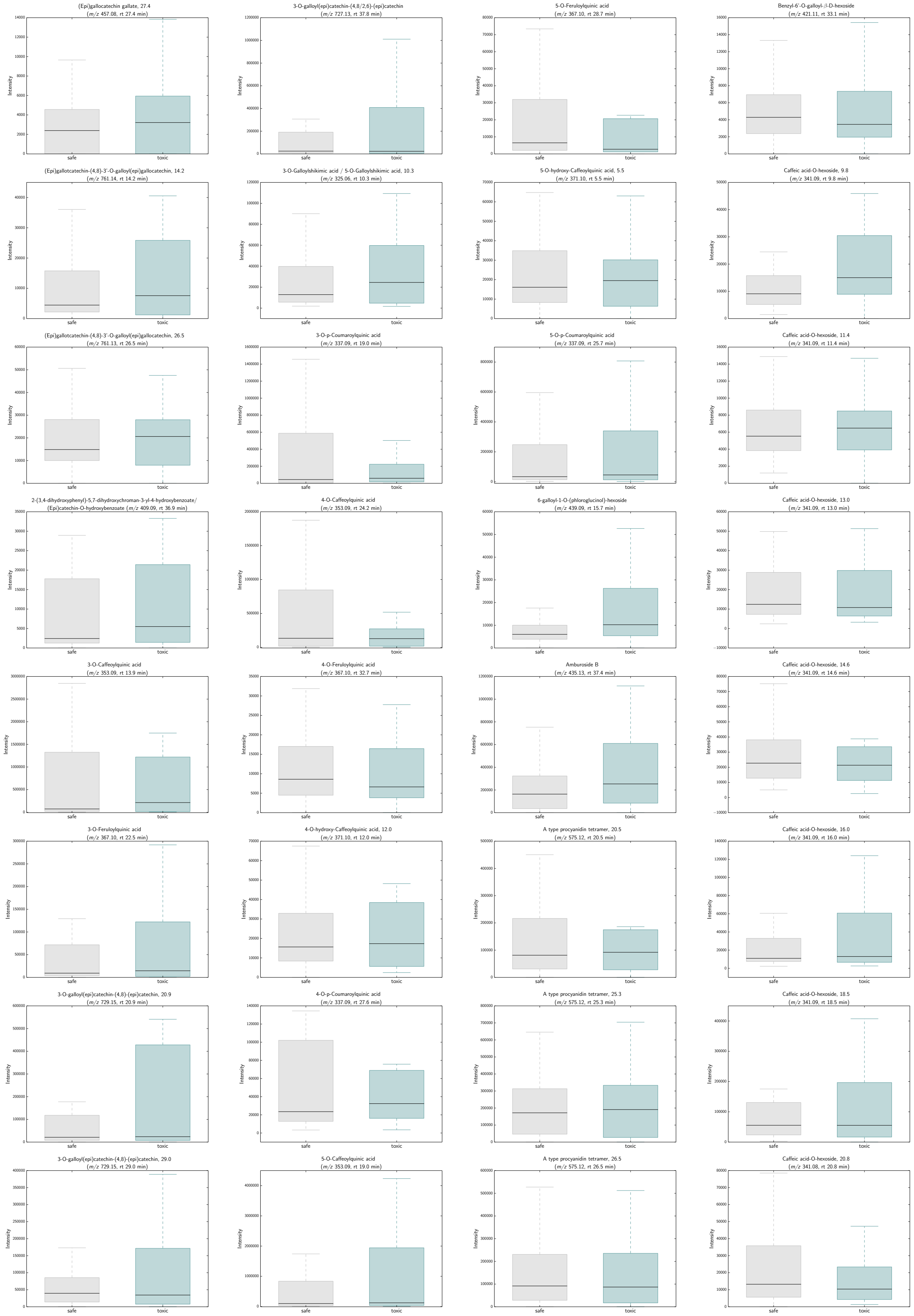
(Epi)gallocatechin gallate, 27.4
(m/z 457.08, rt 27.4 min)
3-O-galloyl(epi)catechin-(4,8/2,6)-(epi)catechin
(m/z 727.13, rt 37.8 min)
5-O-Feruloylquinic acid
(m/z 367.10, rt 28.7 min)
Benzyl-6'-O-galloyl-β-D-hexoside
(m/z 421.11, rt 33.1 min)
(Epi)gallotcatechin-(4,8)-3'-O-galloyl(epi)gallocatechin, 14.2
(m/z 761.14, rt 14.2 min)
3-O-Galloylshikimic acid / 5-O-Galloylshikimic acid, 10.3
(m/z 325.06, rt 10.3 min)
5-O-hydroxy-Caffeoylquinic acid, 5.5
(m/z 371.10, rt 5.5 min)
Caffeic acid-O-hexoside, 9.8
(m/z 341.09, rt 9.8 min)
(Epi)gallotcatechin-(4,8)-3'-O-galloyl(epi)gallocatechin, 26.5
(m/z 761.13, rt 26.5 min)
3-O-p-Coumaroylquinic acid
(m/z 337.09, rt 19.0 min)
5-O-p-Coumaroylquinic acid
(m/z 337.09, rt 25.7 min)
Caffeic acid-O-hexoside, 11.4
(m/z 341.09, rt 11.4 min)
2-(3,4-dihydroxyphenyl)-5,7-dihydroxychroman-3-yl-4-hydroxybenzoate/
(Epi)catechin-O-hydroxybenzoate (m/z 409.09, rt 36.9 min)
4-O-Caffeoylquinic acid
(m/z 353.09, rt 24.2 min)
6-galloyl-1-O-(phloroglucinol)-hexoside
(m/z 439.09, rt 15.7 min)
Caffeic acid-O-hexoside, 13.0
(m/z 341.09, rt 13.0 min)
3-O-Caffeoylquinic acid
(m/z 353.09, rt 13.9 min)
4-O-Feruloylquinic acid
(m/z 367.10, rt 32.7 min)
Amburoside B
(m/z 435.13, rt 37.4 min)
Caffeic acid-O-hexoside, 14.6
(m/z 341.09, rt 14.6 min)
3-O-Feruloylquinic acid
(m/z 367.10, rt 22.5 min)
4-O-hydroxy-Caffeoylquinic acid, 12.0
(m/z 371.10, rt 12.0 min)
A type procyanidin tetramer, 20.5
(m/z 575.12, rt 20.5 min)
Caffeic acid-O-hexoside, 16.0
(m/z 341.09, rt 16.0 min)
3-O-galloyl(epi)catechin-(4,8)-(epi)catechin, 20.9
(m/z 729.15, rt 20.9 min)
4-O-p-Coumaroylquinic acid
(m/z 337.09, rt 27.6 min)
A type procyanidin tetramer, 25.3
(m/z 575.12, rt 25.3 min)
Caffeic acid-O-hexoside, 18.5
(m/z 341.09, rt 18.5 min)
3-O-galloyl(epi)catechin-(4,8)-(epi)catechin, 29.0
(m/z 729.15, rt 29.0 min)
5-O-Caffeoylquinic acid
(m/z 353.09, rt 19.0 min)
A type procyanidin tetramer, 26.5
(m/z 575.12, rt 26.5 min)
Caffeic acid-O-hexoside, 20.8
(m/z 341.08, rt 20.8 min)
Intensity
safe
toxic

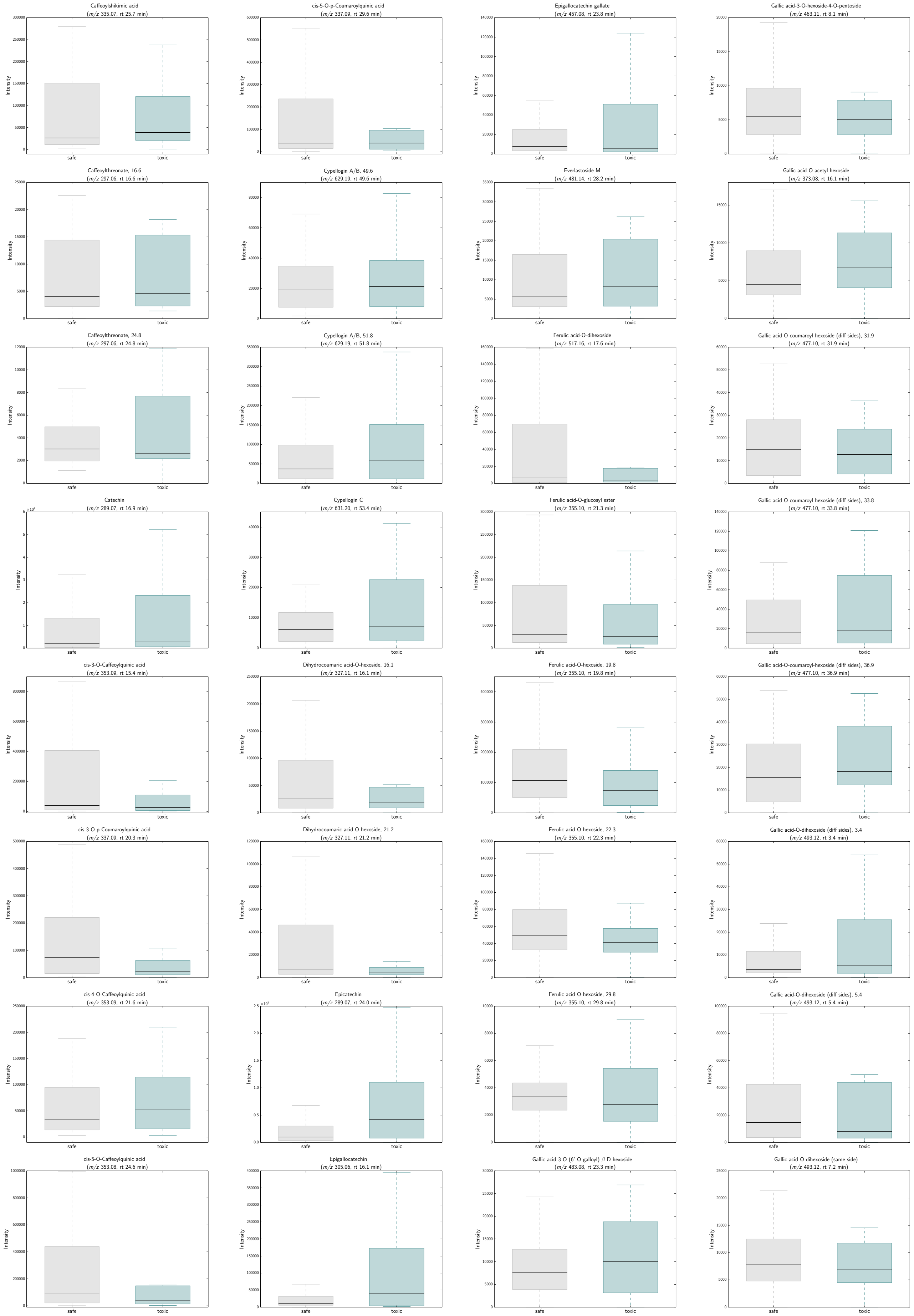
Caffeoylshikimic acid
(m/z 335.07, rt 25.7 min)
cis-5-O-p-Coumaroylquinic acid
(m/z 337.09, rt 29.6 min)
Epigallocatechin gallate
(m/z 457.08, rt 23.8 min)
Gallic acid-3-O-hexoside-4-O-pentoside
(m/z 463.11, rt 8.1 min)
Caffeoylthreonate, 16.6
(m/z 297.06, rt 16.6 min)
Cypellogin A/B, 49.6
(m/z 629.19, rt 49.6 min)
Everlastoside M
(m/z 481.14, rt 28.2 min)
Gallic acid-O-acetyl-hexoside
(m/z 373.08, rt 16.1 min)
Caffeoylthreonate, 24.8
(m/z 297.06, rt 24.8 min)
Cypellogin A/B, 51.8
(m/z 629.19, rt 51.8 min)
Ferulic acid-O-dihexoside
(m/z 517.16, rt 17.6 min)
Gallic acid-O-coumaroyl-hexoside (diff sides), 31.9
(m/z 477.10, rt 31.9 min)
Catechin
(m/z 289.07, rt 16.9 min)
Cypellogin C
(m/z 631.20, rt 53.4 min)
Ferulic acid-O-glucosyl ester
(m/z 355.10, rt 21.3 min)
Gallic acid-O-coumaroyl-hexoside (diff sides), 33.8
(m/z 477.10, rt 33.8 min)
cis-3-O-Caffeoylquinic acid
(m/z 353.09, rt 15.4 min)
Dihydrocoumaric acid-O-hexoside, 16.1
(m/z 327.11, rt 16.1 min)
Ferulic acid-O-hexoside, 19.8
(m/z 355.10, rt 19.8 min)
Gallic acid-O-coumaroyl-hexoside (diff sides), 36.9
(m/z 477.10, rt 36.9 min)
cis-3-O-p-Coumaroylquinic acid
(m/z 337.09, rt 20.3 min)
Dihydrocoumaric acid-O-hexoside, 21.2
(m/z 327.11, rt 21.2 min)
Ferulic acid-O-hexoside, 22.3
(m/z 355.10, rt 22.3 min)
Gallic acid-O-dihexoside (diff sides), 3.4
(m/z 493.12, rt 3.4 min)
cis-4-O-Caffeoylquinic acid
(m/z 353.09, rt 21.6 min)
Epicatechin
(m/z 289.07, rt 24.0 min)
Ferulic acid-O-hexoside, 29.8
(m/z 355.10, rt 29.8 min)
Gallic acid-O-dihexoside (diff sides), 5.4
(m/z 493.12, rt 5.4 min)
cis-5-O-Caffeoylquinic acid
(m/z 353.08, rt 24.6 min)
Epigallocatechin
(m/z 305.06, rt 16.1 min)
Gallic acid-3-O-(6'-O-galloyl)-β-D-hexoside
(m/z 483.08, rt 23.3 min)
Gallic acid-O-dihexoside (same side)
(m/z 493.12, rt 7.2 min)
Intensity
safe
toxic

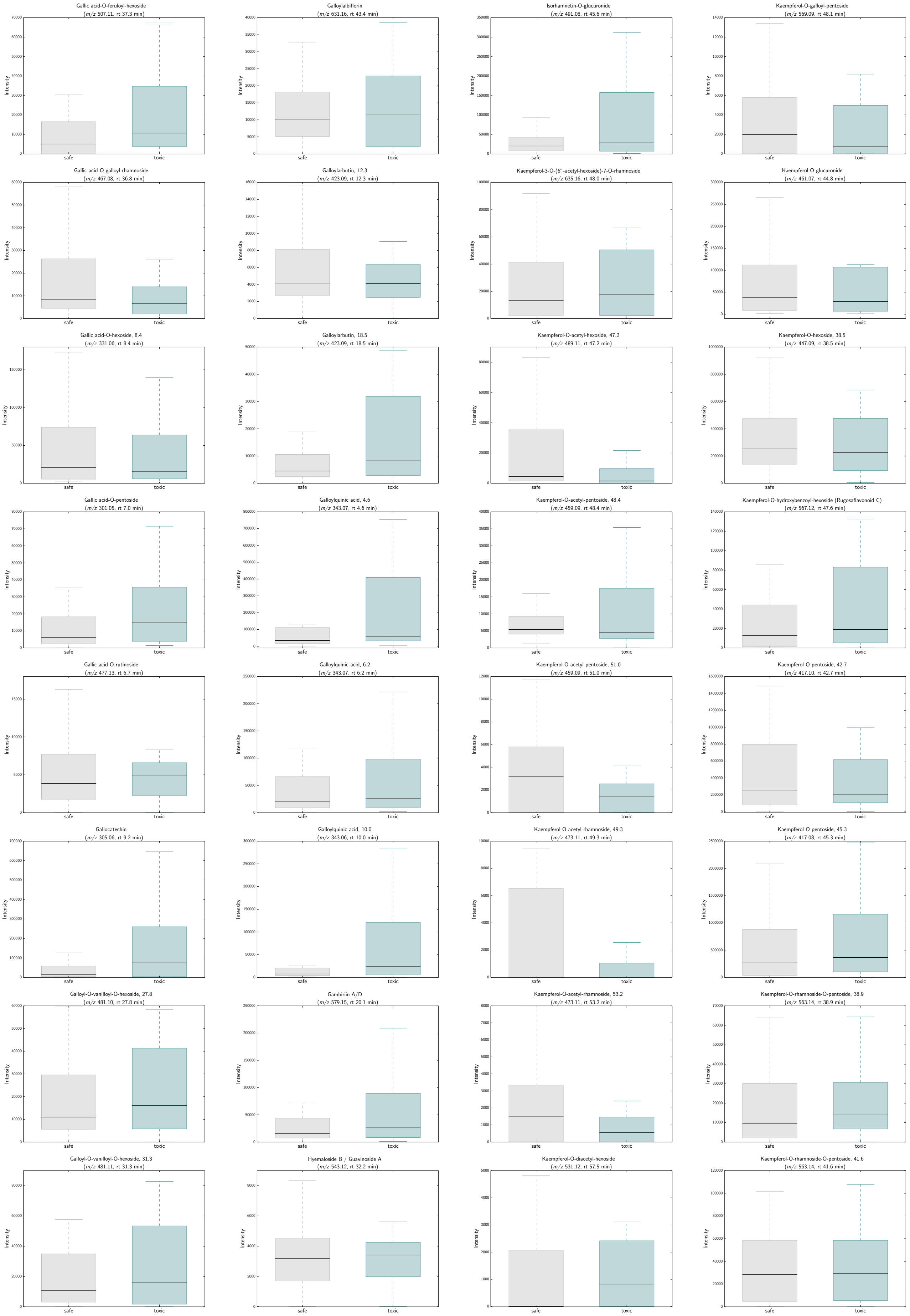

Gallic acid-O-feruloyl-hexoside
(m/z 507.11, rt 37.3 min)
Galloylalbiflorin
(m/z 631.16, rt 43.4 min)
Isorhamnetin-O-glucuronide
(m/z 491.08, rt 45.6 min)
Kaempferol-O-galloyl-pentoside
(m/z 569.09, rt 48.1 min)
Gallic acid-O-galloyl-rhamnoside
(m/z 467.08, rt 36.8 min)
Galloylarbutin, 12.3
(m/z 423.09, rt 12.3 min)
Kaempferol-3-O-(6''-acetyl-hexoside)-7-O-rhamnoside
(m/z 635.16, rt 48.0 min)
Kaempferol-O-glucuronide
(m/z 461.07, rt 44.8 min)
Gallic acid-O-hexoside, 8.4
(m/z 331.06, rt 8.4 min)
Galloylarbutin, 18.5
(m/z 423.09, rt 18.5 min)
Kaempferol-O-acetyl-hexoside, 47.2
(m/z 489.11, rt 47.2 min)
Kaempferol-O-hexoside, 38.5
(m/z 447.09, rt 38.5 min)
Gallic acid-O-pentoside
(m/z 301.05, rt 7.0 min)
Galloylquinic acid, 4.6
(m/z 343.07, rt 4.6 min)
Kaempferol-O-acetyl-pentoside, 48.4
(m/z 459.09, rt 48.4 min)
Kaempferol-O-hydroxybenzoyl-hexoside (Rugosaflavonoid C)
(m/z 567.12, rt 47.6 min)
Gallic acid-O-rutinoside
(m/z 477.13, rt 6.7 min)
Galloylquinic acid, 6.2
(m/z 343.07, rt 6.2 min)
Kaempferol-O-acetyl-pentoside, 51.0
(m/z 459.09, rt 51.0 min)
Kaempferol-O-pentoside, 42.7
(m/z 417.10, rt 42.7 min)
Gallocatechin
(m/z 305.06, rt 9.2 min)
Galloylquinic acid, 10.0
(m/z 343.06, rt 10.0 min)
Kaempferol-O-acetyl-rhamnoside, 49.3
(m/z 473.11, rt 49.3 min)
Kaempferol-O-pentoside, 45.3
(m/z 417.08, rt 45.3 min)
Galloyl-O-vanilloyl-O-hexoside, 27.8
(m/z 481.10, rt 27.8 min)
Gambiriin A/D
(m/z 579.15, rt 20.1 min)
Kaempferol-O-acetyl-rhamnoside, 53.2
(m/z 473.11, rt 53.2 min)
Kaempferol-O-rhamnoside-O-pentoside, 38.9
(m/z 563.14, rt 38.9 min)
Galloyl-O-vanilloyl-O-hexoside, 31.3
(m/z 481.11, rt 31.3 min)
Hyemaloside B / Guavinoside A
(m/z 543.12, rt 32.2 min)
Kaempferol-O-diacetyl-hexoside
(m/z 531.12, rt 57.5 min)
Kaempferol-O-rhamnoside-O-pentoside, 41.6
(m/z 563.14, rt 41.6 min)
Intensity
safe
toxic

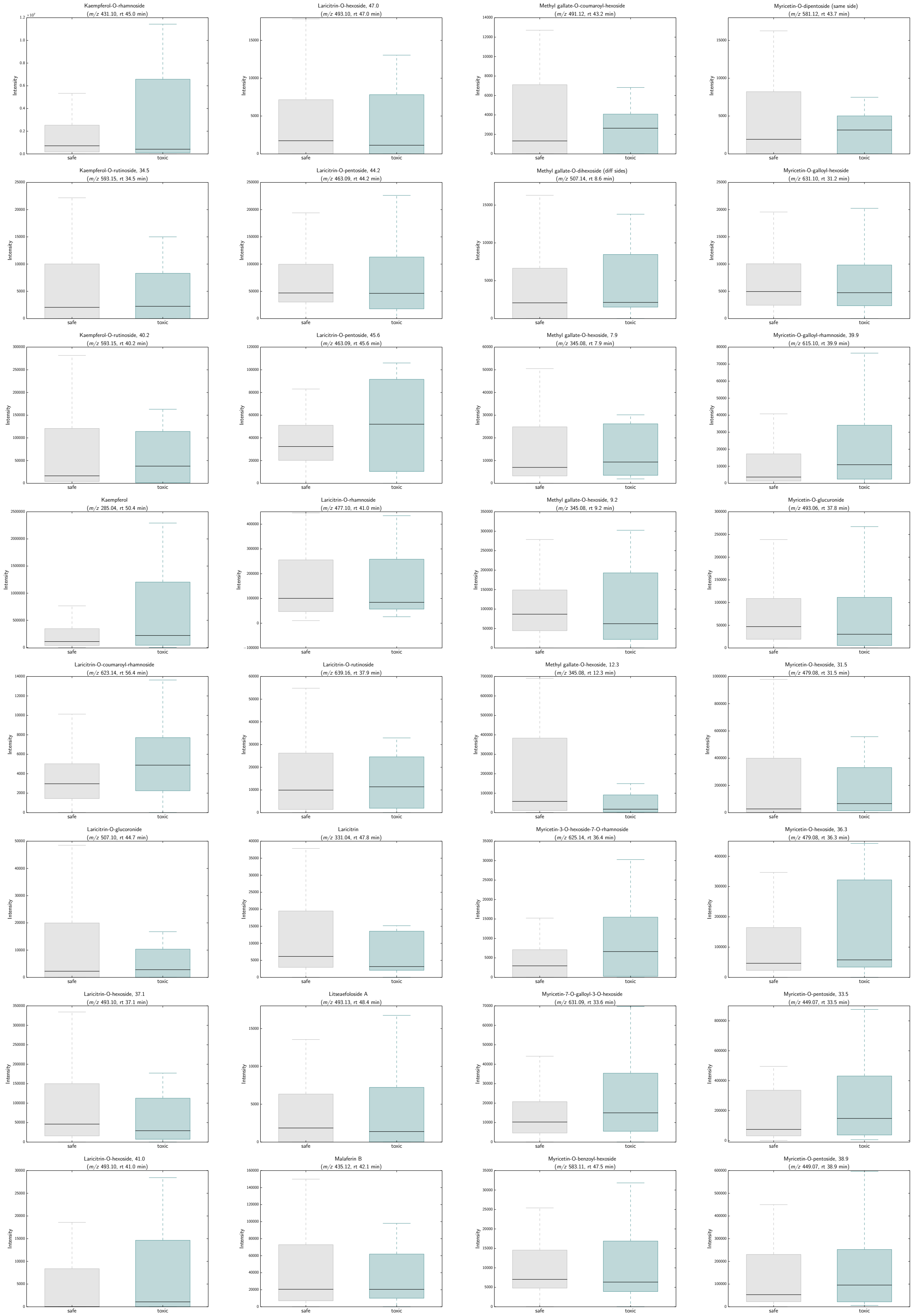
Kaempferol-O-rhamnoside
(m/z 431.10, rt 45.0 min)
Laricitrin-O-hexoside, 47.0
(m/z 493.10, rt 47.0 min)
Methyl gallate-O-coumaroyl-hexoside
(m/z 491.12, rt 43.2 min)
Myricetin-O-dipentoside (same side)
(m/z 581.12, rt 43.7 min)
Kaempferol-O-rutinoside, 34.5
(m/z 593.15, rt 34.5 min)
Laricitrin-O-pentoside, 44.2
(m/z 463.09, rt 44.2 min)
Methyl gallate-O-dihexoside (diff sides)
(m/z 507.14, rt 8.6 min)
Myricetin-O-galloyl-hexoside
(m/z 631.10, rt 31.2 min)
Kaempferol-O-rutinoside, 40.2
(m/z 593.15, rt 40.2 min)
Laricitrin-O-pentoside, 45.6
(m/z 463.09, rt 45.6 min)
Methyl gallate-O-hexoside, 7.9
(m/z 345.08, rt 7.9 min)
Myricetin-O-galloyl-rhamnoside, 39.9
(m/z 615.10, rt 39.9 min)
Kaempferol
(m/z 285.04, rt 50.4 min)
Laricitrin-O-rhamnoside
(m/z 477.10, rt 41.0 min)
Methyl gallate-O-hexoside, 9.2
(m/z 345.08, rt 9.2 min)
Myricetin-O-glucuronide
(m/z 493.06, rt 37.8 min)
Laricitrin-O-coumaroyl-rhamnoside
(m/z 623.14, rt 56.4 min)
Laricitrin-O-rutinoside
(m/z 639.16, rt 37.9 min)
Methyl gallate-O-hexoside, 12.3
(m/z 345.08, rt 12.3 min)
Myricetin-O-hexoside, 31.5
(m/z 479.08, rt 31.5 min)
Laricitrin-O-glucoronide
(m/z 507.10, rt 44.7 min)
Laricitrin
(m/z 331.04, rt 47.8 min)
Myricetin-3-O-hexoside-7-O-rhamnoside
(m/z 625.14, rt 36.4 min)
Myricetin-O-hexoside, 36.3
(m/z 479.08, rt 36.3 min)
Laricitrin-O-hexoside, 37.1
(m/z 493.10, rt 37.1 min)
Litseaefoloside A
(m/z 493.13, rt 48.4 min)
Myricetin-7-O-galloyl-3-O-hexoside
(m/z 631.09, rt 33.6 min)
Myricetin-O-pentoside, 33.5
(m/z 449.07, rt 33.5 min)
Laricitrin-O-hexoside, 41.0
(m/z 493.10, rt 41.0 min)
Malaferin B
(m/z 435.12, rt 42.1 min)
Myricetin-O-benzoyl-hexoside
(m/z 583.11, rt 47.5 min)
Myricetin-O-pentoside, 38.9
(m/z 449.07, rt 38.9 min)
Intensity
safe
toxic

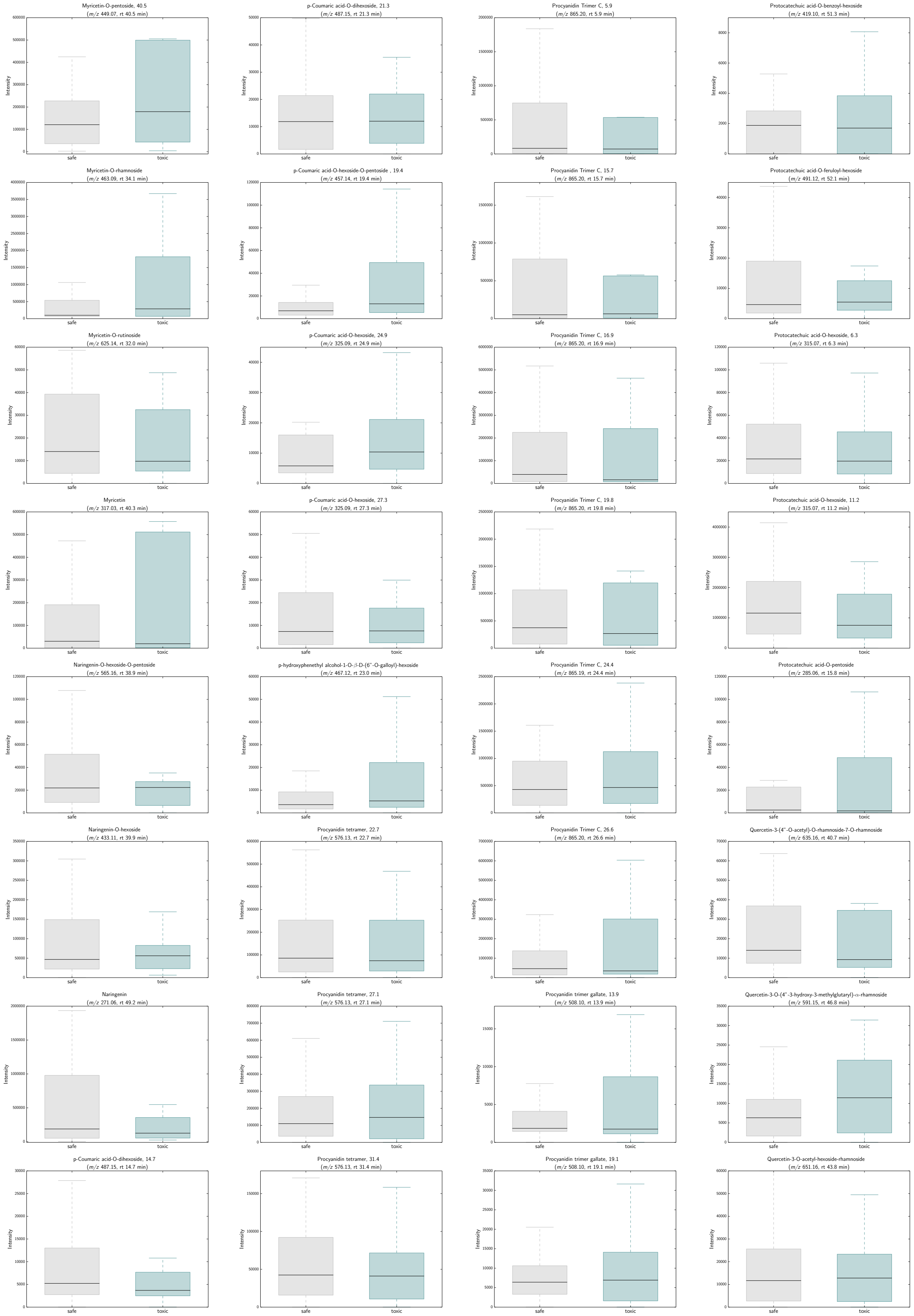
Myricetin-O-pentoside, 40.5
(m/z 449.07, rt 40.5 min)
p-Coumaric acid-O-dihexoside, 21.3
(m/z 487.15, rt 21.3 min)
Procyanidin Trimer C, 5.9
(m/z 865.20, rt 5.9 min)
Protocatechuic acid-O-benzoyl-hexoside
(m/z 419.10, rt 51.3 min)
Myricetin-O-rhamnoside
(m/z 463.09, rt 34.1 min)
p-Coumaric acid-O-hexoside-O-pentoside , 19.4
(m/z 457.14, rt 19.4 min)
Procyanidin Trimer C, 15.7
(m/z 865.20, rt 15.7 min)
Protocatechuic acid-O-feruloyl-hexoside
(m/z 491.12, rt 52.1 min)
Myricetin-O-rutinoside
(m/z 625.14, rt 32.0 min)
p-Coumaric acid-O-hexoside, 24.9
(m/z 325.09, rt 24.9 min)
Procyanidin Trimer C, 16.9
(m/z 865.20, rt 16.9 min)
Protocatechuic acid-O-hexoside, 6.3
(m/z 315.07, rt 6.3 min)
Myricetin
(m/z 317.03, rt 40.3 min)
p-Coumaric acid-O-hexoside, 27.3
(m/z 325.09, rt 27.3 min)
Procyanidin Trimer C, 19.8
(m/z 865.20, rt 19.8 min)
Protocatechuic acid-O-hexoside, 11.2
(m/z 315.07, rt 11.2 min)
Naringenin-O-hexoside-O-pentoside
(m/z 565.16, rt 38.9 min)
p-hydroxyphenethyl alcohol-1-O-β-D-(6''-O-galloyl)-hexoside
(m/z 467.12, rt 23.0 min)
Procyanidin Trimer C, 24.4
(m/z 865.19, rt 24.4 min)
Protocatechuic acid-O-pentoside
(m/z 285.06, rt 15.8 min)
Naringenin-O-hexoside
(m/z 433.11, rt 39.9 min)
Procyanidin tetramer, 22.7
(m/z 576.13, rt 22.7 min)
Procyanidin Trimer C, 26.6
(m/z 865.20, rt 26.6 min)
Quercetin-3-(4''-O-acetyl)-O-rhamnoside-7-O-rhamnoside
(m/z 635.16, rt 40.7 min)
Naringenin
(m/z 271.06, rt 49.2 min)
Procyanidin tetramer, 27.1
(m/z 576.13, rt 27.1 min)
Procyanidin trimer gallate, 13.9
(m/z 508.10, rt 13.9 min)
Quercetin-3-O-(4''-3-hydroxy-3-methylglutaryl)-α-rhamnoside
(m/z 591.15, rt 46.8 min)
p-Coumaric acid-O-dihexoside, 14.7
(m/z 487.15, rt 14.7 min)
Procyanidin tetramer, 31.4
(m/z 576.13, rt 31.4 min)
Procyanidin trimer gallate, 19.1
(m/z 508.10, rt 19.1 min)
Quercetin-3-O-acetyl-hexoside-rhamnoside
(m/z 651.16, rt 43.8 min)

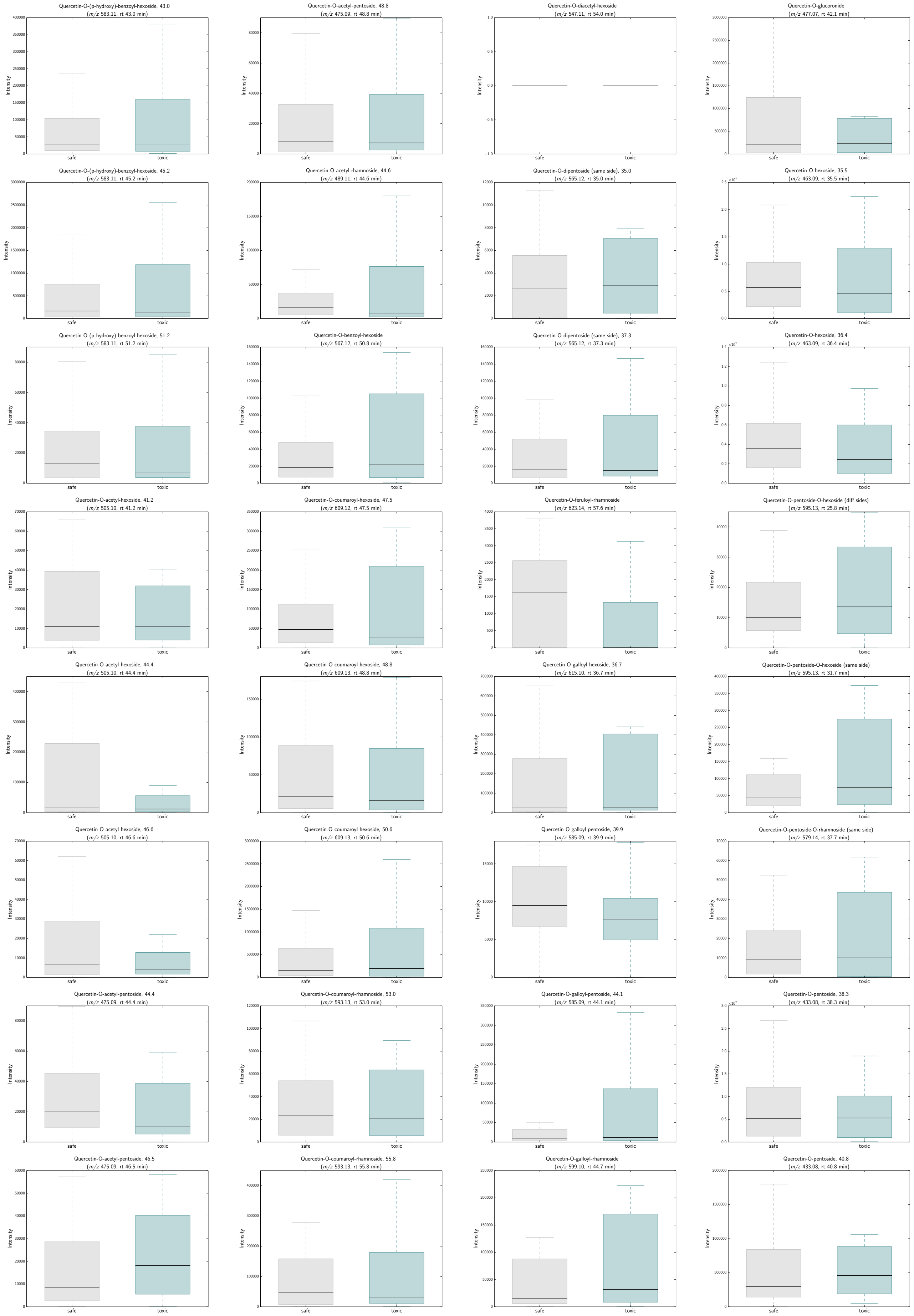
Quercetin-O-(p-hydroxy)-benzoyl-hexoside, 43.0
(m/z 583.11, rt 43.0 min)
Quercetin-O-acetyl-pentoside, 48.8
(m/z 475.09, rt 48.8 min)
Quercetin-O-diacetyl-hexoside
(m/z 547.11, rt 54.0 min)
Quercetin-O-glucoronide
(m/z 477.07, rt 42.1 min)
Quercetin-O-(p-hydroxy)-benzoyl-hexoside, 45.2
(m/z 583.11, rt 45.2 min)
Quercetin-O-acetyl-rhamnoside, 44.6
(m/z 489.11, rt 44.6 min)
Quercetin-O-dipentoside (same side), 35.0
(m/z 565.12, rt 35.0 min)
Quercetin-O-hexoside, 35.5
(m/z 463.09, rt 35.5 min)
Quercetin-O-(p-hydroxy)-benzoyl-hexoside, 51.2
(m/z 583.11, rt 51.2 min)
Quercetin-O-benzoyl-hexoside
(m/z 567.12, rt 50.8 min)
Quercetin-O-dipentoside (same side), 37.3
(m/z 565.12, rt 37.3 min)
Quercetin-O-hexoside, 36.4
(m/z 463.09, rt 36.4 min)
Quercetin-O-acetyl-hexoside, 41.2
(m/z 505.10, rt 41.2 min)
Quercetin-O-coumaroyl-hexoside, 47.5
(m/z 609.12, rt 47.5 min)
Quercetin-O-feruloyl-rhamnoside
(m/z 623.14, rt 57.6 min)
Quercetin-O-pentoside-O-hexoside (diff sides)
(m/z 595.13, rt 25.8 min)
Quercetin-O-acetyl-hexoside, 44.4
(m/z 505.10, rt 44.4 min)
Quercetin-O-coumaroyl-hexoside, 48.8
(m/z 609.13, rt 48.8 min)
Quercetin-O-galloyl-hexoside, 36.7
(m/z 615.10, rt 36.7 min)
Quercetin-O-pentoside-O-hexoside (same side)
(m/z 595.13, rt 31.7 min)
Quercetin-O-acetyl-hexoside, 46.6
(m/z 505.10, rt 46.6 min)
Quercetin-O-coumaroyl-hexoside, 50.6
(m/z 609.13, rt 50.6 min)
Quercetin-O-galloyl-pentoside, 39.9
(m/z 585.09, rt 39.9 min)
Quercetin-O-pentoside-O-rhamnoside (same side)
(m/z 579.14, rt 37.7 min)
Quercetin-O-acetyl-pentoside, 44.4
(m/z 475.09, rt 44.4 min)
Quercetin-O-coumaroyl-rhamnoside, 53.0
(m/z 593.13, rt 53.0 min)
Quercetin-O-galloyl-pentoside, 44.1
(m/z 585.09, rt 44.1 min)
Quercetin-O-pentoside, 38.3
(m/z 433.08, rt 38.3 min)
Quercetin-O-acetyl-pentoside, 46.5
(m/z 475.09, rt 46.5 min)
Quercetin-O-coumaroyl-rhamnoside, 55.8
(m/z 593.13, rt 55.8 min)
Quercetin-O-galloyl-rhamnoside
(m/z 599.10, rt 44.7 min)
Quercetin-O-pentoside, 40.8
(m/z 433.08, rt 40.8 min)
Intensity
safe
toxic

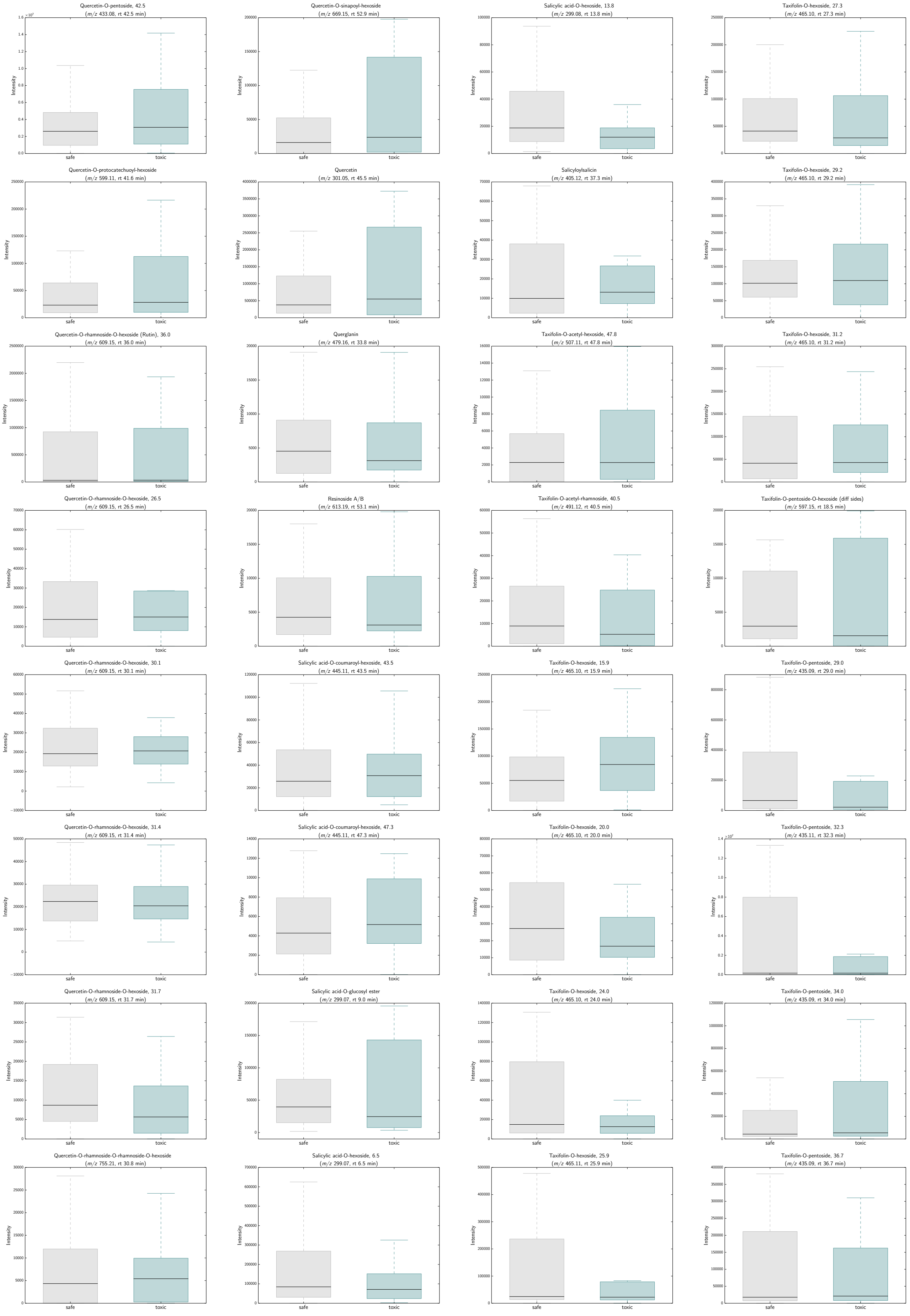
Quercetin-O-pentoside, 42.5
(m/z 433.08, rt 42.5 min)
Quercetin-O-sinapoyl-hexoside
(m/z 669.15, rt 52.9 min)
Salicylic acid-O-hexoside, 13.8
(m/z 299.08, rt 13.8 min)
Taxifolin-O-hexoside, 27.3
(m/z 465.10, rt 27.3 min)
Quercetin-O-protocatechuoyl-hexoside
(m/z 599.11, rt 41.6 min)
Quercetin
(m/z 301.05, rt 45.5 min)
Salicyloylsalicin
(m/z 405.12, rt 37.3 min)
Taxifolin-O-hexoside, 29.2
(m/z 465.10, rt 29.2 min)
Quercetin-O-rhamnoside-O-hexoside (Rutin), 36.0
(m/z 609.15, rt 36.0 min)
Querglanin
(m/z 479.16, rt 33.8 min)
Taxifolin-O-acetyl-hexoside, 47.8
(m/z 507.11, rt 47.8 min)
Taxifolin-O-hexoside, 31.2
(m/z 465.10, rt 31.2 min)
Quercetin-O-rhamnoside-O-hexoside, 26.5
(m/z 609.15, rt 26.5 min)
Resinoside A/B
(m/z 613.19, rt 53.1 min)
Taxifolin-O-acetyl-rhamnoside, 40.5
(m/z 491.12, rt 40.5 min)
Taxifolin-O-pentoside-O-hexoside (diff sides)
(m/z 597.15, rt 18.5 min)
Quercetin-O-rhamnoside-O-hexoside, 30.1
(m/z 609.15, rt 30.1 min)
Salicylic acid-O-coumaroyl-hexoside, 43.5
(m/z 445.11, rt 43.5 min)
Taxifolin-O-hexoside, 15.9
(m/z 465.10, rt 15.9 min)
Taxifolin-O-pentoside, 29.0
(m/z 435.09, rt 29.0 min)
Quercetin-O-rhamnoside-O-hexoside, 31.4
(m/z 609.15, rt 31.4 min)
Salicylic acid-O-coumaroyl-hexoside, 47.3
(m/z 445.11, rt 47.3 min)
Taxifolin-O-hexoside, 20.0
(m/z 465.10, rt 20.0 min)
Taxifolin-O-pentoside, 32.3
(m/z 435.11, rt 32.3 min)
Quercetin-O-rhamnoside-O-hexoside, 31.7
(m/z 609.15, rt 31.7 min)
Salicylic acid-O-glucosyl ester
(m/z 299.07, rt 9.0 min)
Taxifolin-O-hexoside, 24.0
(m/z 465.10, rt 24.0 min)
Taxifolin-O-pentoside, 34.0
(m/z 435.09, rt 34.0 min)
Quercetin-O-rhamnoside-O-rhamnoside-O-hexoside
(m/z 755.21, rt 30.8 min)
Salicylic acid-O-hexoside, 6.5
(m/z 299.07, rt 6.5 min)
Taxifolin-O-hexoside, 25.9
(m/z 465.11, rt 25.9 min)
Taxifolin-O-pentoside, 36.7
(m/z 435.09, rt 36.7 min)
Intensity
safe
toxic

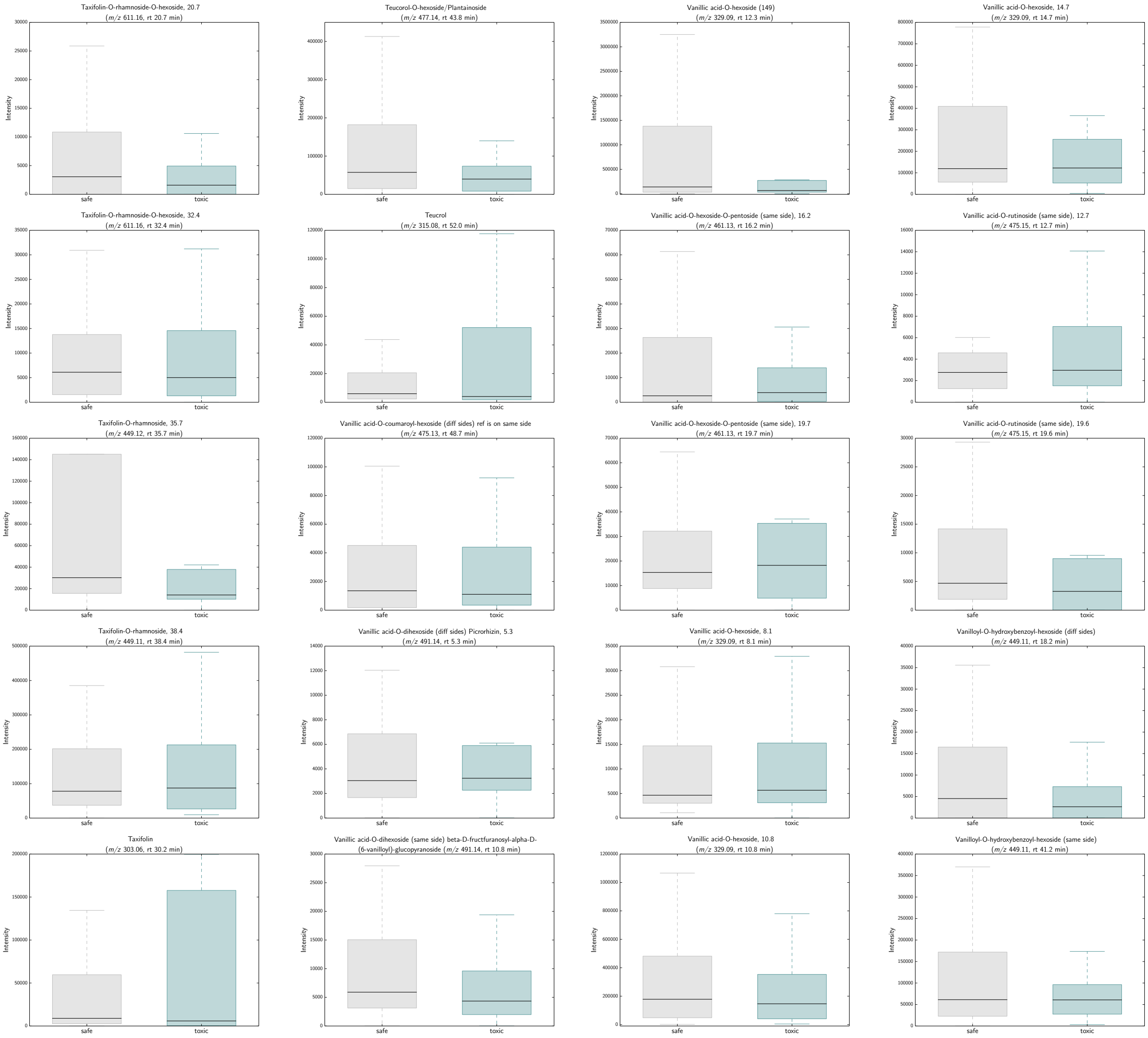

Figure S6 | Box plots of all 292 identified polyphenolics showing no significant difference in LC-MS intesity with respect to cytotoxicity classification towards IEC-6 cells of all 87 *Rhododendron* species divided in cytotoxic (22 species, green) and non-cytotoxic (65 species, gray).

Table S1 | Average and standard deviation of mass-to-charge (m/z) ratios and retention times (rt) for most-predictive LC-MS peaks regarding antimicrobial activity as well as cytotoxicity towards HaCaT and IEC-6 cells.

	# peaks	m/z ratio	rt [min]
Antimicrobial activity	23	399.69 ± 98.97	63.0 ± 6.7
Cytotoxicity towards HaCaT cells	26	719.60 ± 202.91	45.5 ± 15.2
Cytotoxicity towards IEC-6 cells	13	557.92 ± 237.94	34.9 ± 14.9

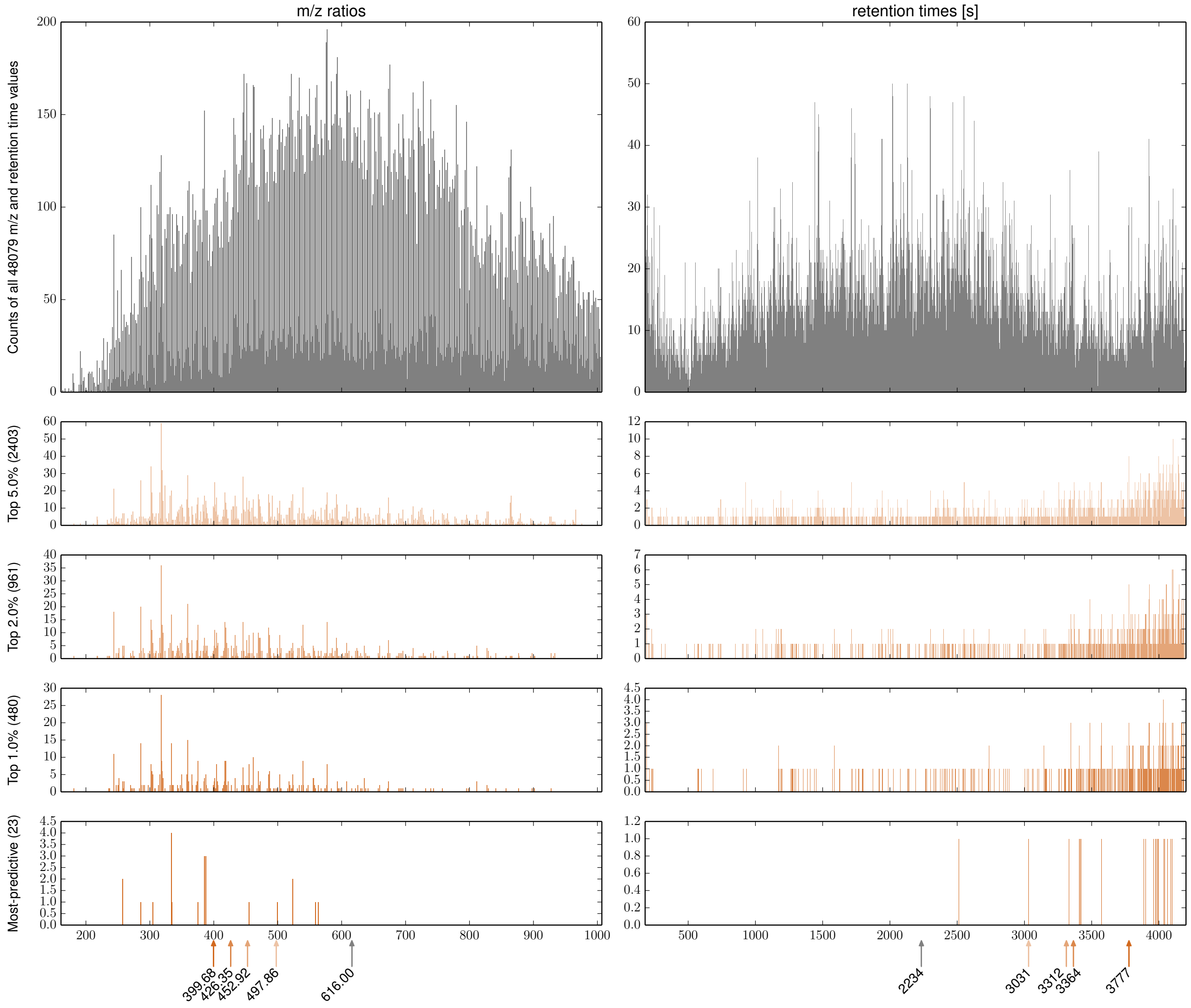

Figure S7 | Distribution of the detected LC-MS peaks (grey) regarding m/z ratios (left) and retention times (right). In addition, the m/z ratio and retention time of the 23 most-predictive peaks as well as top 1%, 2% and 5% peaks (dark orange to bright orange) with respect to Cohen's κ correlation to antimicrobial activity across all 87 *Rhododendron* species are shown. The arrows denote the average m/z ratio and retention time, respectively.

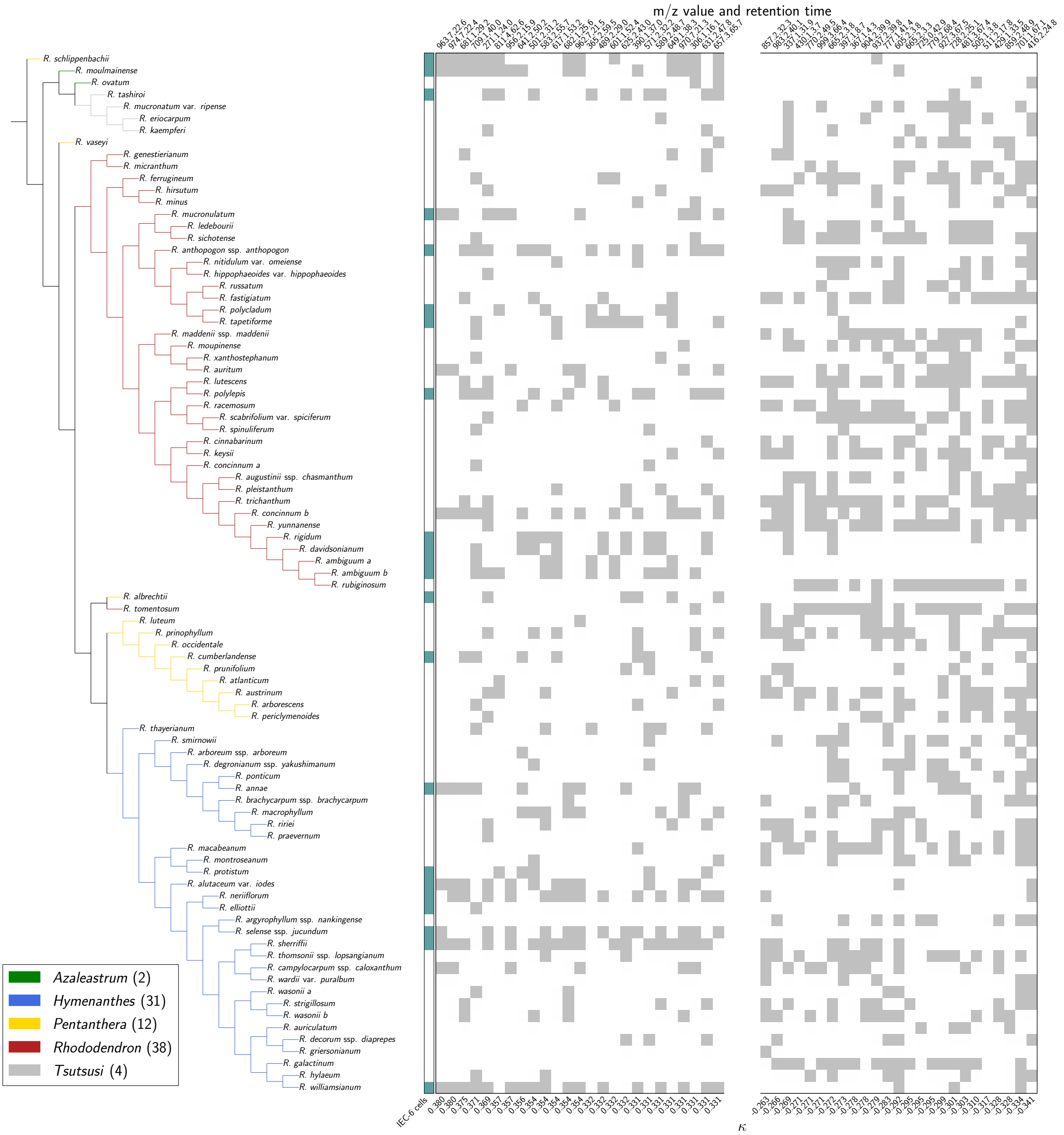

Figure S8 | The 25 most- and least-predictive LC-MS peaks with respect to Cohen's κ correlation for cytotoxicity towards IEC-6 cells across all 87 *Rhododendron* species. A sample is denoted as cytotoxic towards IEC-6 cells (green) if the MTT assay is significantly dropped. A compound, defined by an *m/z* ratio and retention time tuple, is denoted as present in a sample (gray) if its intensity is ≥ 10000. The * represents the significance of the p-values according to multiple testing correction by Benjamini-Hochberg (0.05).

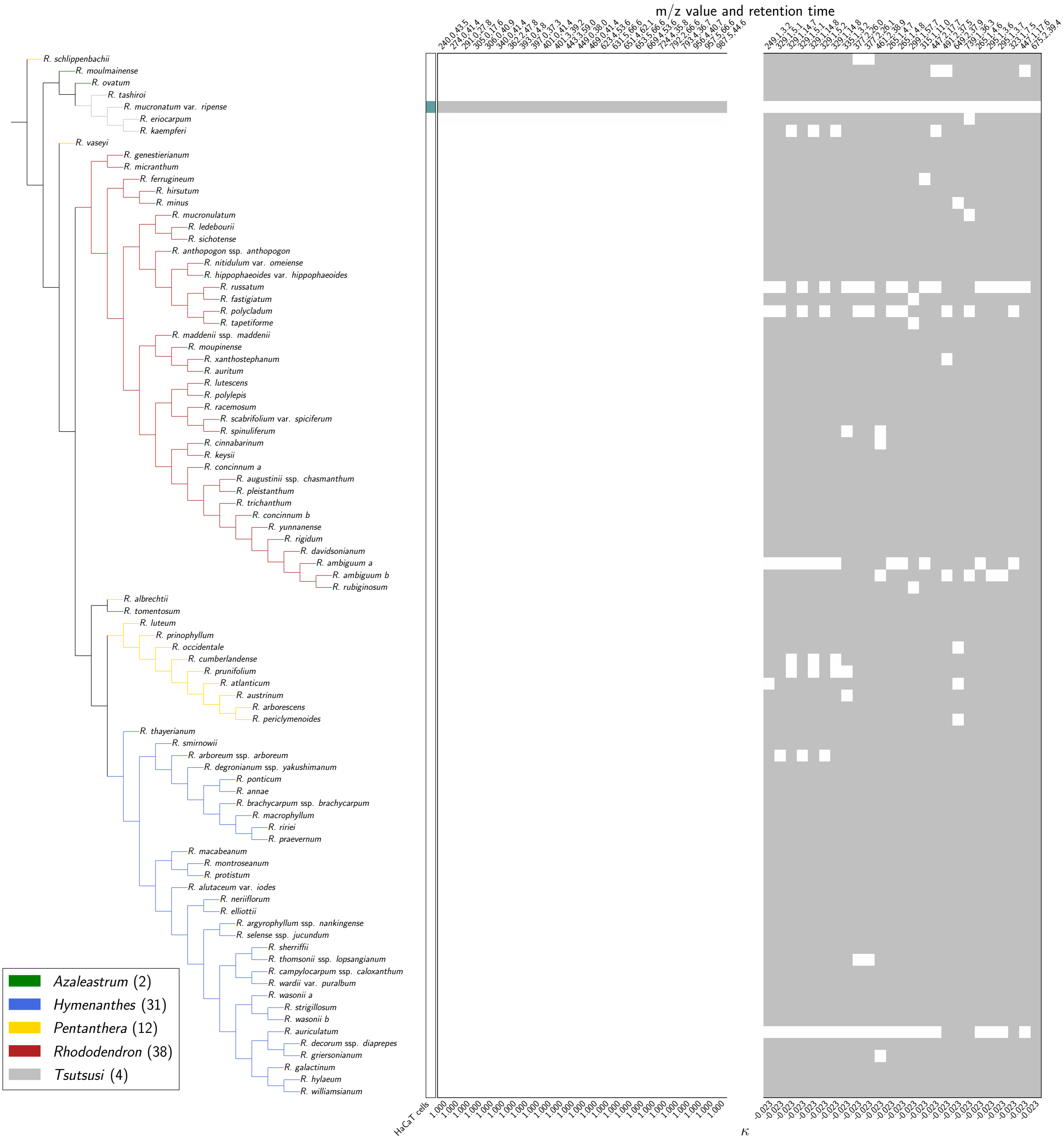

Figure S9 | The 25 most- and least-predictive LC-MS peaks with respect to Cohen's κ correlation for cytotoxicity towards HaCaT cells across all 87 *Rhododendron* species. A sample is denoted as cytotoxic towards HaCaT cells (green) if the MTT assay is significantly dropped. A compound, defined by an m/z ratio and retention time tuple, is denoted as present in a sample (gray) if its intensity is ≥ 10000. The * represents the significance of the p-values according to multiple testing correction by Benjamini-Hochberg (0.05).

Table S2 | The 23 most-predictive LC-MS peaks with respect to Cohen's κ correlation for antimicrobial activity across all 87 *Rhododendron* species. The peaks are uniquely determined by m/z ratio and retention time (rt) and have attributed the rank and correlation coefficient, κ, for antimicrobial activity, cytotoxicity towards HaCaT and IEC-6 cells. The highlighted rows depict the seven most-predictive peaks for antimicrobial active but non-cytotoxic compounds.

Peak		Antimicrobial activity		Cytotoxictiy HaCaT		Cytotoxictiy IEC-6	
m/z ratio	rt [min]	rank	κ	rank	κ	rank	κ
333.19	64.8	1	0.7704	31117	−0.0220	7956	0.0821
455.20	66.0	1	0.7704	31117	−0.0220	7956	0.0821
523.19	67.7	3	0.7601	34746	−0.0223	25643	−0.0036
333.22	67.3	4	0.7592	29299	−0.0218	3987	0.1205
257.16	66.5	5	0.7468	27352	−0.0215	1812	0.1617
257.20	66.5	5	0.7468	27352	−0.0215	1812	0.1617
387.22	56.8	7	0.7383	31907	−0.0221	3205	0.1307
334.17	68.3	8	0.7258	30246	−0.0219	5717	0.1010
304.16	67.3	9	0.7200	34075	−0.0223	21733	0.0124
375.22	59.5	10	0.7121	28363	−0.0217	9472	0.0691
387.16	57.0	11	0.7076	32668	−0.0222	4652	0.1119
499.17	68.4	11	0.7076	32668	−0.0222	42896	−0.0854
285.09	50.5	13	0.6969	26230	−0.0213	4851	0.1092
333.16	66.4	13	0.6969	26230	−0.0213	1103	0.1834
333.20	66.2	13	0.6969	26230	−0.0213	1103	0.1834
384.95	56.9	13	0.6969	26230	−0.0213	4851	0.1092
384.99	66.6	13	0.6969	26230	−0.0213	4851	0.1092
384.99	68.3	13	0.6969	26230	−0.0213	4851	0.1092
559.15	65.0	13	0.6969	26230	−0.0213	1103	0.1834
607.16	66.2	13	0.6969	26230	−0.0213	4851	0.1092
387.23	55.5	21	0.6939	31117	−0.0220	2267	0.1501
563.17	41.9	21	0.6939	31117	−0.0220	21419	0.0141
523.21	68.3	23	0.6916	1777	0.0750	46192	−0.1291

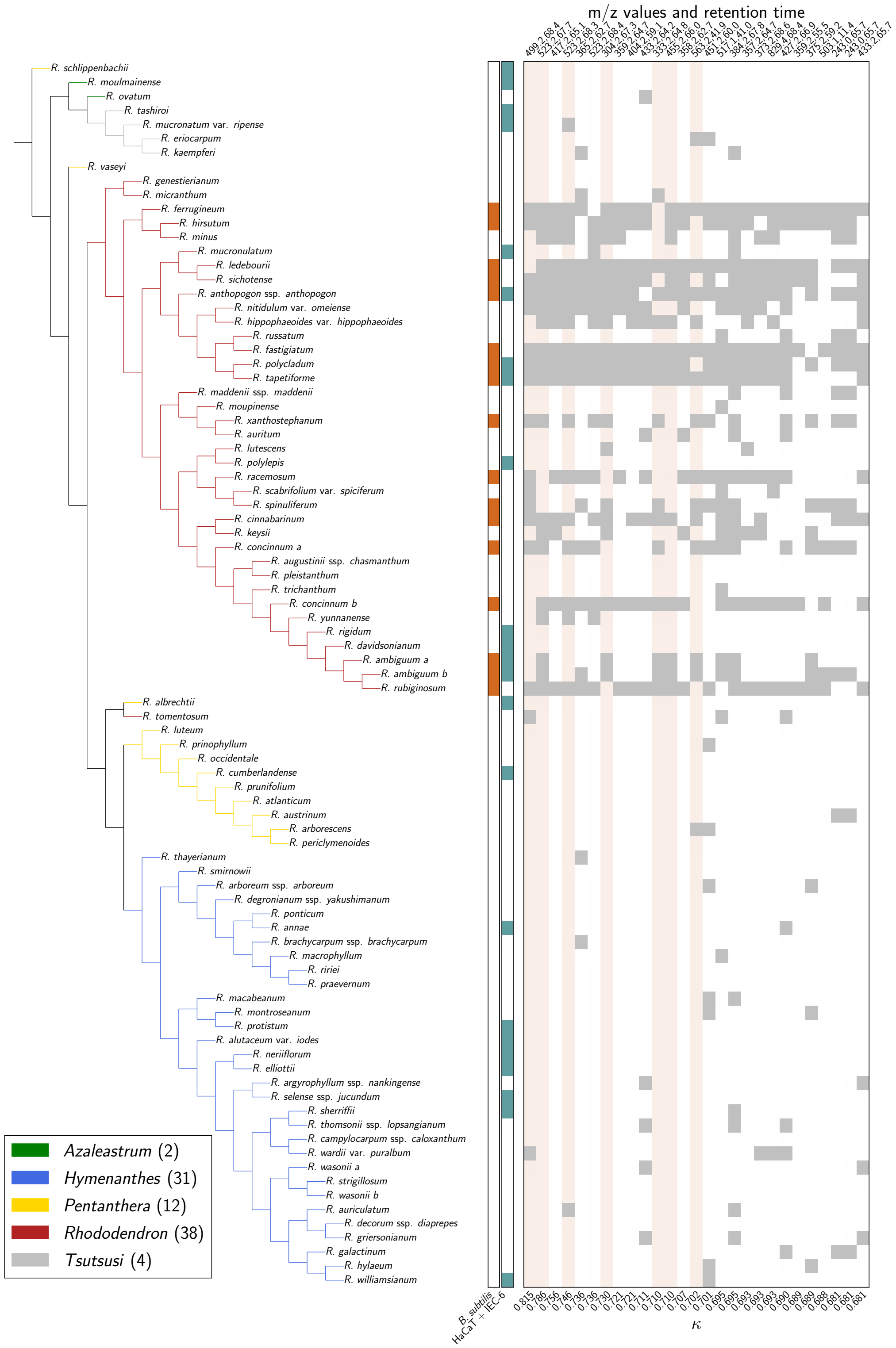

Figure S10 | The 27 most-predictive LC-MS peaks with a strong combined relation signal (≥ 0.68) for antimicrobial activity and non-cytotoxicity towards both cell lines across all 87 *Rhododendron* species (see Material and Methods). A sample is denoted as antimicrobial active (orange, left lane) if the radius of the agar diffusion assay is ≥ 0.6 cm and as cytotoxic towards HaCaT or IEC-6 cells (green, right lane) if the MTT assay is significantly dropped. A compound, defined by an m/z ratio and retention time tuple, is denoted as present in a sample (gray) if its intensity is ≥ 10,000. The seven most-predictive peaks regarding antimicrobial activity and non-cytotoxicity at once are highlighted in bright orange.

Table S3 | 27 most-predictive LC-MS peaks with respect to the combined relation signal for antimicrobial activity and non-cytotoxicity towards both cell lines across all 87 *Rhododendron* species. The peaks are uniquely determined by *m/z* ratio and retention time (rt) and have attributed the combined correlation coefficient, κ, and the individual ranks for antimicrobial activity and the cytotoxicity towards HaCaT and IEC-6 cells. The highlighted rows depict the seven most-predictive peaks for antimicrobial active but non-cytotoxic compounds.

Peak		Combined	Individual ranks		
m/z ratio	rt [min]	κ	AM	HaCaT	IEC-6
499.17	68.4	0.8152	11	32668	42896
523.19	67.7	0.7861	3	34746	25643
417.17	65.1	0.7556	33	31907	40552
523.21	68.3	0.7457	23	1777	46192
365.19	62.7	0.7362	27	33392	33332
523.19	68.4	0.7362	27	33392	33332
304.16	67.3	0.7299	9	34075	21733
359.16	64.7	0.7210	24	29299	28761
404.22	59.1	0.7210	24	29299	28761
433.22	64.2	0.7111	560	34075	47583
333.19	64.8	0.7104	1	31117	7956
455.20	66.0	0.7104	1	31117	7956
358.21	62.7	0.7067	46	30246	33644
563.17	41.9	0.7019	21	31117	21419
451.17	60.0	0.7006	185	35392	42037
517.11	41.0	0.6949	64	36005	33108
384.25	67.8	0.6949	42	39555	28111
357.22	64.7	0.6934	104	31117	37580
373.20	68.6	0.6934	104	31117	37580
829.40	68.4	0.6934	104	31117	37580
427.23	66.9	0.6900	265	36005	43695
359.22	55.5	0.6893	429	22316	46572
375.16	59.2	0.6887	33	31907	25424
503.10	11.4	0.6882	204	23675	41567
242.95	65.7	0.6808	192	31907	40552
242.99	65.7	0.6808	192	31907	40552
433.22	65.7	0.6808	192	31907	40552

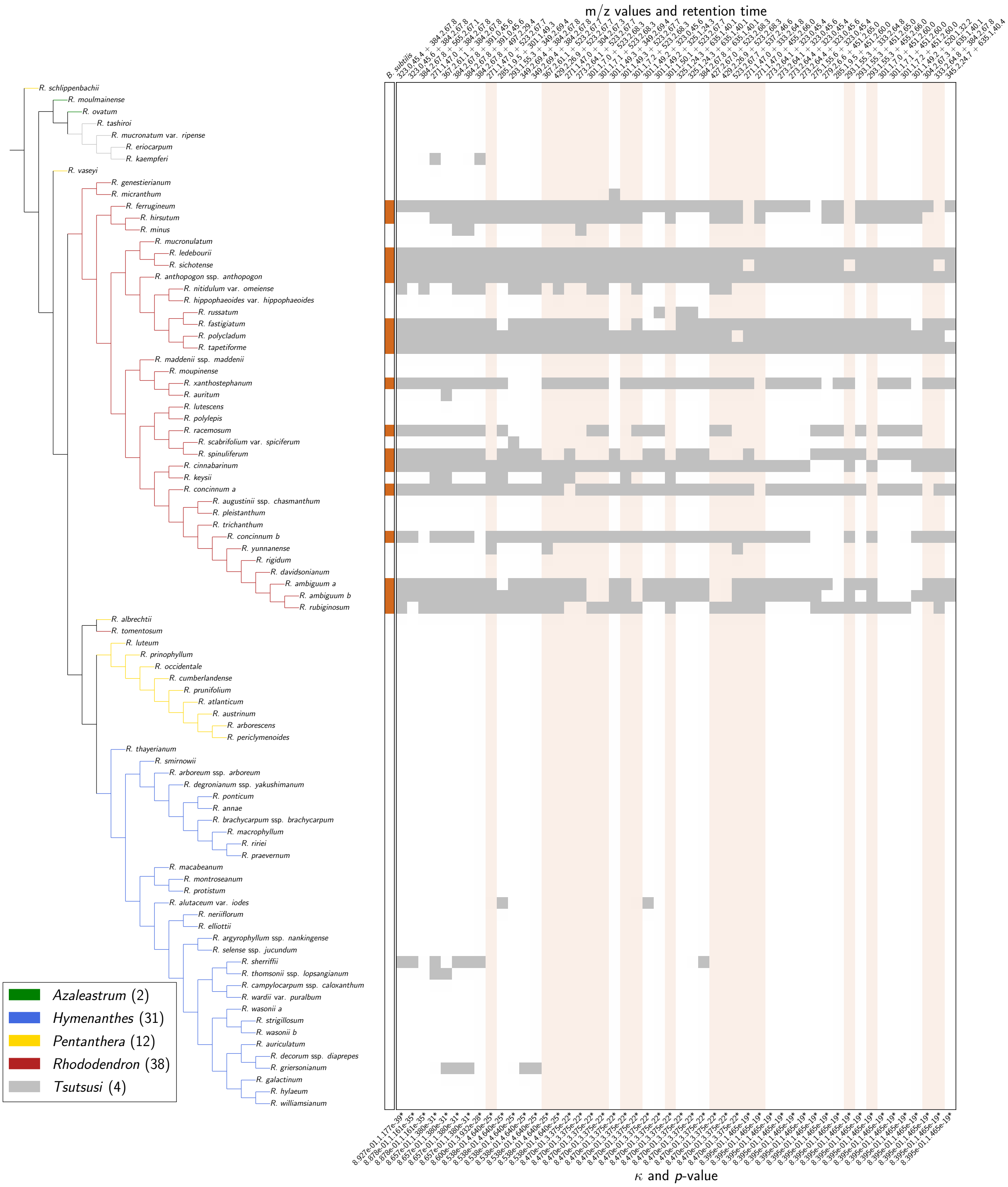

Figure S11 | The 50 most-predictive peak combinations with additive effects with respect to Cohen's κ correlation for antimicrobial activity across all 87 *Rhododendron* species. A sample is denoted as antimicrobial active (orange) if the radius of the agar diffusion assay is $\geq 0.6\ cm$. A compound, defined by an *m/z* ratio and retention time tuple, is denoted as present in a sample (gray) if its intensity is ≥ 10000. The * represents the significance of the p-values according to multiple testing correction by Benjamini-Hochberg (0.05). The combinations comprising one of the seven most-predictive peaks regarding antimicrobial activity and non-cytotoxicity at once are highlighted in bright orange.

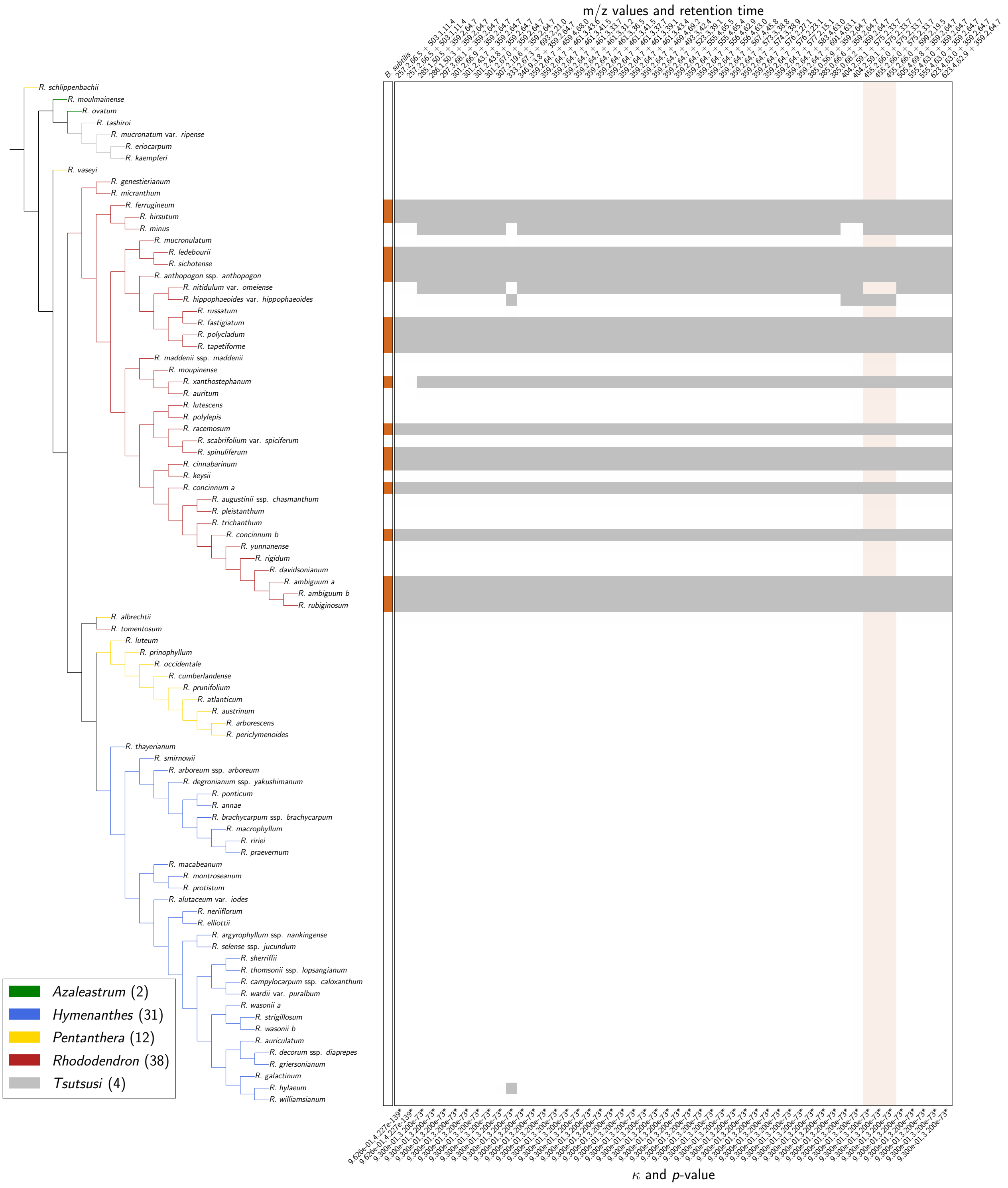

Figure S12 | The 50 most-predictive peak combinations with alternative effects with respect to Cohen's κ correlation for antimicrobial activity across all 87 *Rhododendron* species. A sample is denoted as antimicrobial active (orange) if the radius of the agar diffusion assay is $\geq 0.6\ cm$. A compound, defined by an m/z ratio and retention time tuple, is denoted as present in a sample (gray) if its intensity is ≥ 10000. The * represents the significance of the p-values according to multiple testing correction by Benjamini-Hochberg (0.05). The combinations comprising one of the seven most-predictive peaks regarding antimicrobial activity and non-cytotoxicity at once are highlighted in bright orange.

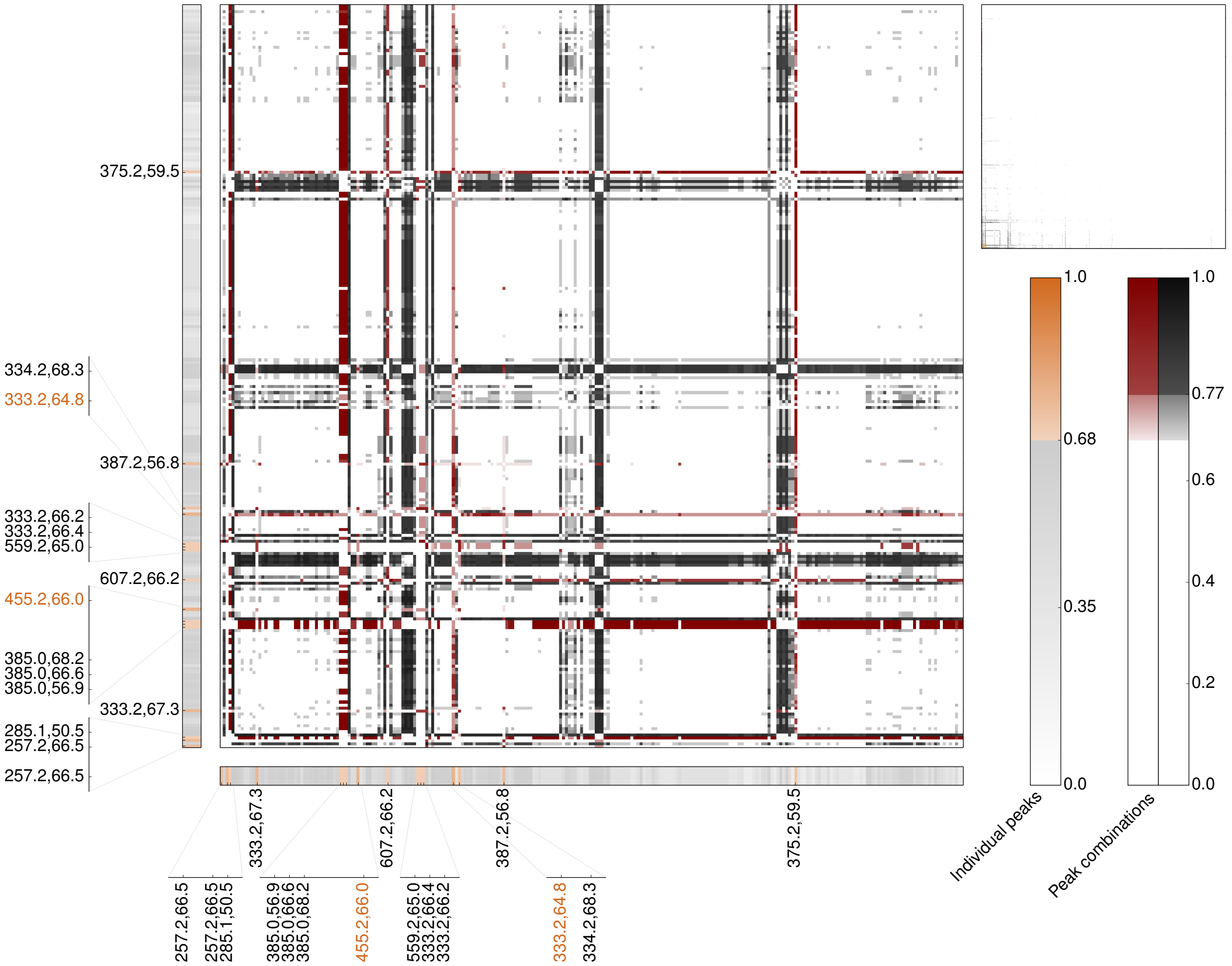

Figure S13 | Heatmap of the top 250 LC-MS peaks involved in the most-predictive alternative peak combinations regarding Cohen's κ correlation for antimicrobial activity across all 87 *Rhododendron* species (main panel). The upper right panel provides the overview of all 5,414 peaks included in the outperforming alternative peak combinations, namely $\kappa \geq 0.77$. The combinations highlighted in red involve at least one of the 23 most-predictive peaks regarding the individual peak analysis, $\kappa \geq 0.68$. The corresponding individual peak correlation coefficients are depicted in the thinner horizontal and vertical panels. Orange labels emphasize the two out of seven most-predictive peaks regarding antimicrobial activity and non-cytotoxicity.

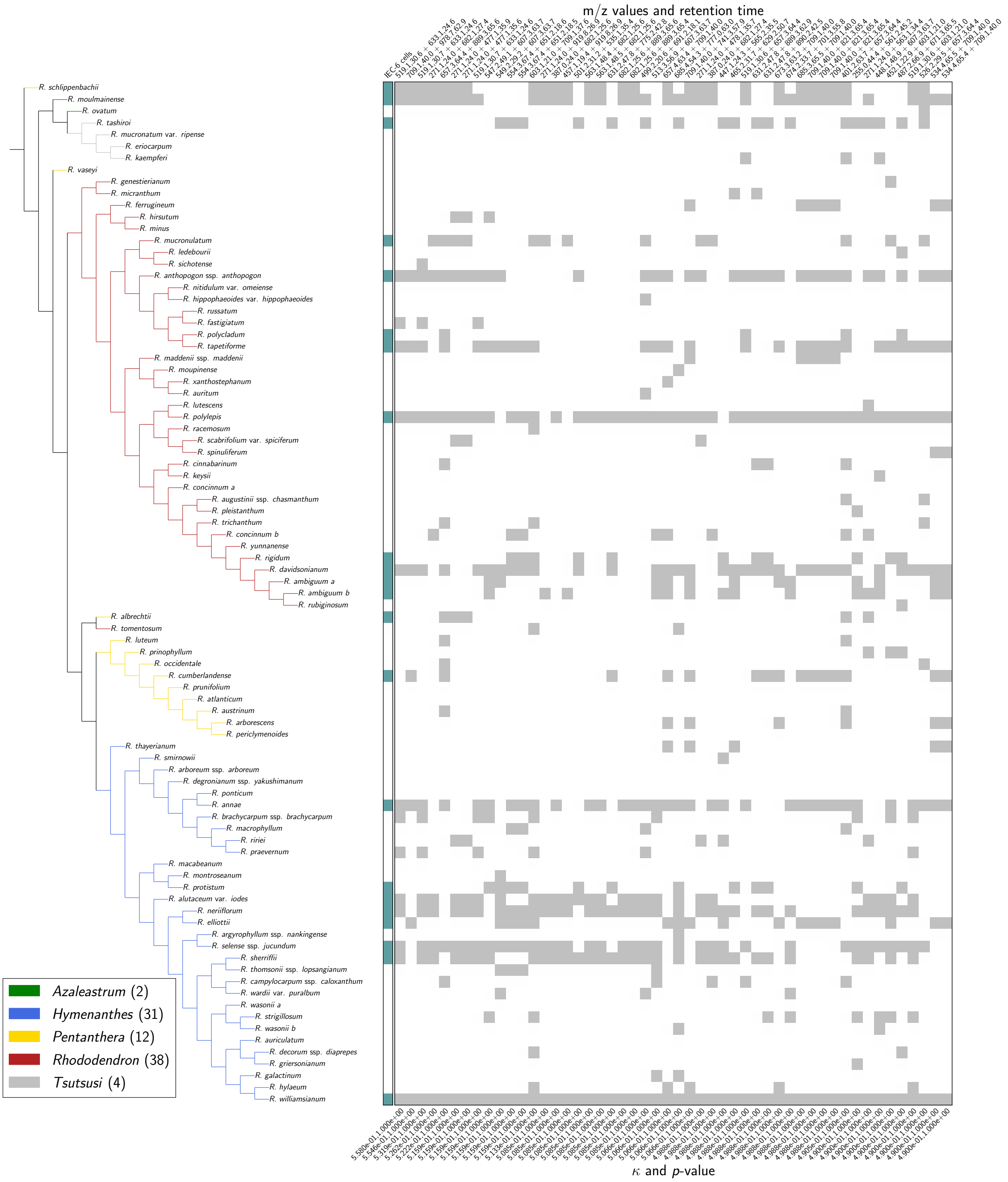

Figure S14 | The 50 most-predictive LC-MS peak combinations with <u>additive effects</u> with respect to Cohen's κ correlation for cytotoxicity towards IEC-6 cells across all 87 *Rhododendron* species. A sample is denoted as cytotoxic towards IEC-6 cells (green) if the MTT assay is significantly dropped. A compound, defined by an m/z ratio and retention time tuple, is denoted as present in a sample (gray) if its intensity is ≥ 10000. The * represents the significance of the p-values according to multiple correction by Benjamini-Hochberg (0.05).

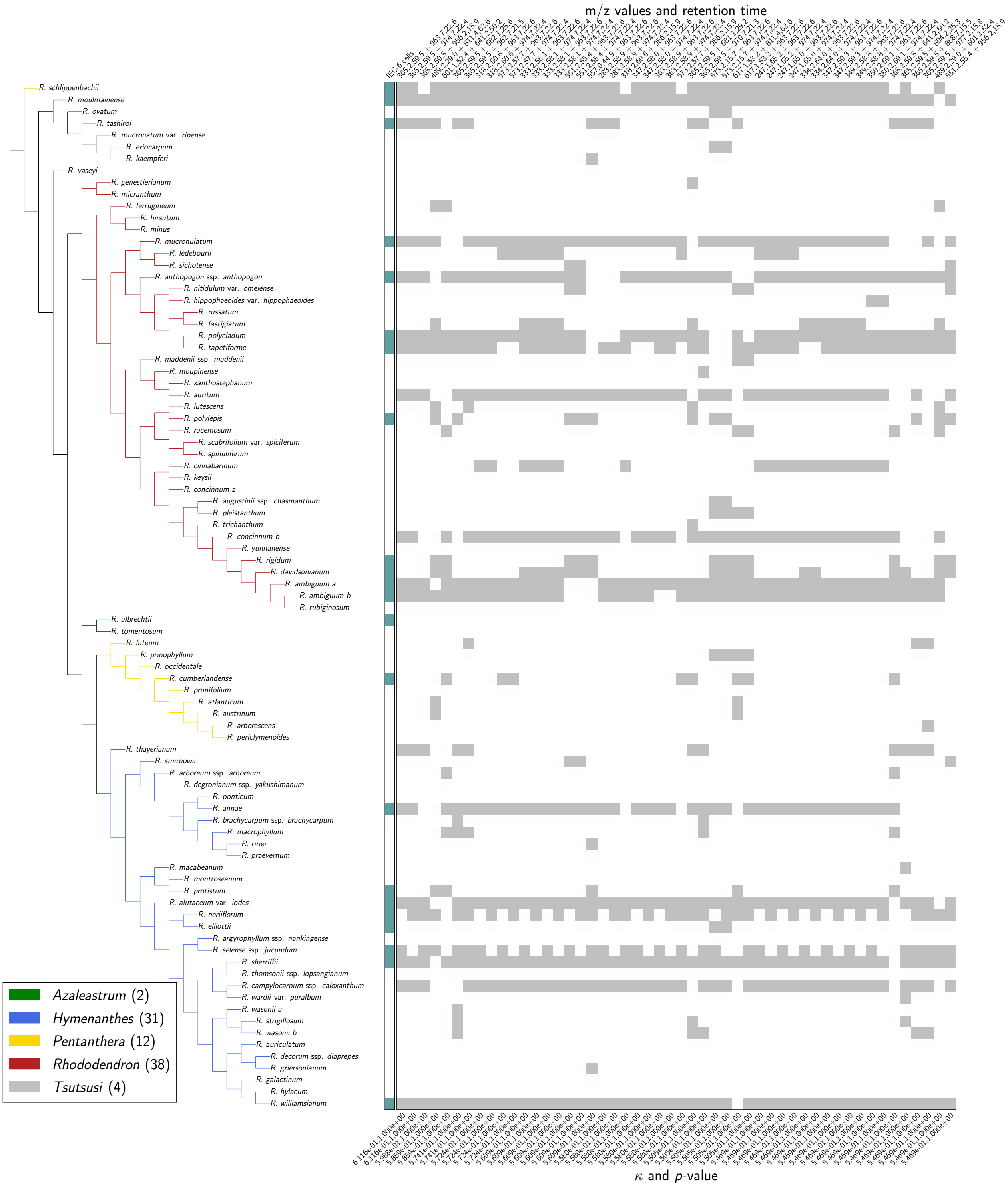

Figure S15 | The 50 most-predictive LC-MS peak combinations with alternative effects with respect to Cohen's κ correlation for cytotoxicity towards IEC-6 cells across all 87 *Rhododendron* species. A sample is denoted as cytotoxic towards IEC-6 cells (green) if the MTT assay is significantly dropped. A compound, defined by an m/z ratio and retention time tuple, is denoted as present in a sample (gray) if its intensity is ≥ 10000. The * represents the significance of the p-values according to multiple testing correction by Benjamini-Hochberg (0.05).

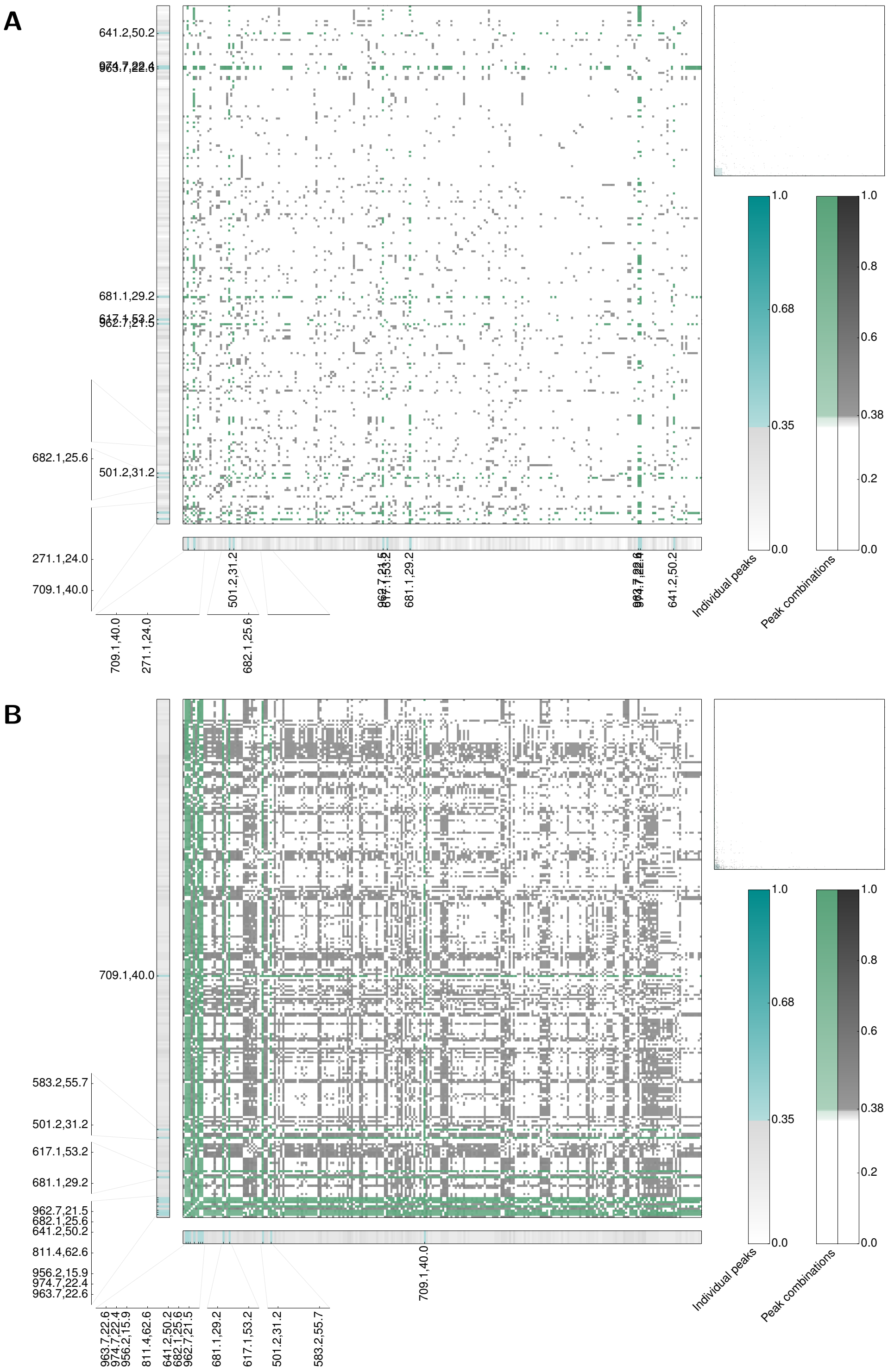

Figure S16 | Heatmap of the top 250 LC-MS peaks involved in the most-predictive (A) additive and (B) alternative peak combinations regarding Cohen's κ correlation for cytotoxicity towards IEC-6 cells across all 87 Rhododendron species (main panel). The respective upper right panel provides the overview of all 7,763 and 8,665 peaks included in the outperforming additive and alternative peak combinations, namely $\kappa \geq 0.38$. The combinations highlighted in green involve at least one of the 13 most-predictive peaks regarding the individual peak analysis, $\kappa \geq 0.35$. The corresponding individual peak correlation coefficients are depicted in the thinner horizontal and vertical panels.

Table S4 | The 12 out of 23 most-predictive individual LC-MS peaks involved in the most-predictive additive peak combinations regarding antimicrobial activity across all 87 *Rhododendron* species. The peaks are uniquely determined by *m/z* ratio and retention time (rt) and have attributed the respective maximum Cohen's κ correlation coefficient with respect to 'AND' operation, κ, and the (minimum) ranks for antimicrobial activity of 'AND' combination and individual (single) occurence. The highlighted rows depict the seven most-predictive peaks for antimicrobial active but non-cytotoxic compounds.

m/z ratio	rt [min]	κ	min $rk_{'AND'}$	$rk_{'single'}$
523.19	67.7	0.8538	9	3
304.16	67.3	0.8470	16	9
523.21	68.3	0.8470	16	23
333.19	64.8	0.8395	32	1
455.20	66.0	0.8395	32	1
387.16	57.0	0.8395	32	11
387.22	56.8	0.8395	32	7
499.17	68.4	0.8041	159	11
333.22	67.3	0.7943	317	4
334.17	68.3	0.7943	317	8
563.17	41.9	0.7943	317	21
387.23	55.5	0.7943	317	21

Table S5 | The 23 most-predictive individual LC-MS peaks involved in tge most-predictive alternative peak combinations regarding antimicrobial activity across all 87 *Rhododendron* species. The peaks are uniquely determined by *m/z* ratio and retention time (rt) and have attributed the respective maximum Cohen's κ correlation coefficient with respect to 'OR' operation, κ, and the (minimum) ranks for antimicrobial activity of 'OR' combination and individual (single) occurence. The highlighted rows depict the seven most-predictive peaks for antimicrobial active but non-cytotoxic compounds.

m/z ratio	rt [min]	κ	min $rk_{'OR'}$	$rk_{'single'}$
257.16	66.5	0.9626	1	5
257.20	66.5	0.9626	1	5
285.09	50.5	0.9300	3	13
333.22	67.3	0.9300	3	4
384.95	56.9	0.9300	3	13
384.99	66.6	0.9300	3	13
384.99	68.2	0.9300	3	13
455.20	66.0	0.9300	3	1
607.16	66.2	0.9235	64	13
559.15	65.0	0.9235	64	13
333.16	66.4	0.9235	64	13
333.20	66.2	0.9235	64	13
333.19	64.8	0.8972	160	1
334.17	68.3	0.8972	160	8
387.22	56.8	0.8972	160	7
375.22	59.5	0.8927	224	10
499.17	68.4	0.8657	4025	11
387.16	57.0	0.8657	4025	11
387.23	55.5	0.8657	4025	21
563.17	41.9	0.8657	4025	21
523.19	67.7	0.8355	15781	3
523.21	58.3	0.8065	32005	23
304.16	67.3	0.8065	32005	9

Table S6 | The 6 identified caffeoylquinic acids (CQA) acting additive in LC-MS peak combinations (AND).

CQA		Peak in combination		Cohen's κ		
m/z ratio	rt [min]	*m/z* ratio	rt [min]	$\kappa_{'CQA'}$	$\kappa_{'single'}$	$\kappa_{'AND'}$
353.08	24.6	455.20	66.9	−0.0640	0.7704	0.7258
353.09	13.9	384.25	67.8	0.0175	0.6538	0.6916
353.09	15.4	433.22	65.7	0.0106	0.5887	0.6475
353.09	19.0	387.22	56.8	−0.0497	0.7383	0.7258
353.09	21.6	359.16	64.7	−0.0086	0.6790	0.6790
353.09	24.2	285.10	9.5	0.0235	0.6183	0.6650

Table S7 | The 15 identified regioisomeric chlorogenic acids (CGA) acting as functional alternative structures in LC-MS peak combinations (OR).

CGA		CGA in combination		Cohen's κ		
m/z ratio	rt [min]	*m/z* ratio	rt [min]	$\kappa_{\text{'CGA'}}$	$\kappa_{\text{'CGA'}}$	$\kappa_{\text{'OR'}}$
337.09	19.0	367.10	28.7	−0.0200	0.0433	0.0386
337.09	20.3	353.09	13.9	−0.0783	0.0175	0.0110
337.09	21.9	367.10	32.7	0.0372	−0.1932	−0.0760
337.09	25.7	353.09	21.6	−0.0086	−0.0086	0.0173
337.09	27.6	353.09	13.9	−0.0792	0.0175	0.0112
337.09	29.6	353.09	24.2	−0.1233	0.0235	0.0052
353.08	24.6	337.09	25.7	−0.0640	−0.0086	−0.0007
353.09	13.9	337.09	19.0	−0.0175	0.0200	0.0374
353.09	15.4	337.09	25.7	0.0106	−0.0086	0.0052
353.09	19.0	337.09	25.7	−0.0497	−0.0086	−0.0007
353.09	21.6	337.09	25.7	−0.0086	−0.0086	0.0173
353.09	24.2	367.10	22.5	0.0235	−0.0097	0.0173
367.10	22.5	337.09	19.0	0.0177	−0.0097	−0.0200
367.10	28.7	337.09	19.0	0.0386	0.0433	−0.0200
367.10	32.7	353.09	24.2	0.0052	−0.1932	0.0235

Table S8 | Quercetin (Q) and the 16 identified quercetin-O-glycosides (QG) acting as functional alternative structures in LC-MS peak combinations (OR).

Q(G)		QG in combination				
m/z ratio	rt [min]	*m/z* ratio	rt [min]	$\kappa_{\text{'Q(G)'}}$	$\kappa_{\text{'CG'}}$	$\kappa_{\text{'OR'}}$
301.05	45.5	433.08	42.5	−0.1105	0.0056	0.0056
433.08	35.1	433.08	42.5	−0.0249	0.0056	0.0056
433.08	38.3	433.08	42.5	−0.0177	0.0056	0.0056
433.08	40.8	609.15	31.7	−0.0121	0.0089	0.0114
433.08	42.5	301.05	45.5	0.0056	−0.1105	0.0056
447.10	40.0	433.08	42.5	−0.0903	0.0056	0.0056
463.09	35.5	433.08	42.5	−0.0903	0.0056	0.0056
463.09	36.4	433.08	42.5	−0.0903	0.0056	0.0056
463.09	41.9	433.08	42.5	−0.1154	0.0056	0
609.12	47.5	433.08	42.5	−0.0601	0.0056	0.0056
609.13	48.8	609.15	36.0	0.0261	−0.1333	−0.1008
609.13	50.6	433.08	42.5	−0.1266	0.0056	0.0056
609.15	26.5	433.08	42.5	−0.3533	0.0056	0.0056
609.15	30.1	433.08	40.8	0.0110	−0.0121	0.0056
609.15	31.4	609.15	36.0	0.0235	−0.1333	0.0172
609.15	31.7	433.08	40.8	0.0089	−0.0121	0.0114
609.15	36.0	609.15	31.4	−0.1333	0.0235	0.0172

Table S9 | Sequence generation for phylogenetic analysis including DNA regions, primer sequences, and respective PCR protocols.

Region	Primer sequence (starting from 5' end)			Initialization	Amplification cycles		Final elongation
*mat*K	*trn*K707F	ACT GTA TCG CAC TAT GTA TCA	Milne et al. (2010)	94°C, 2min	30×	94°C, 30s;	72°C, 7min
	*trn*K2R	AAC TAG TCG GAT GGA GTA G	Johnson and Soltis (1995)			61.5°C, 1min;	
	MK1447F	CGC TCA ATA TCT TCT GAA ACC TT	Milne et al. (2010)[a]			72°C, 1min	
	MK1645R	AGC CAA AAT GGC TTT TCC TC	Milne et al. (2010)[a]				
	MK1538F	TAT GGG TGT TTA AAG AGC	[b]				
	MK1785R	TCT ATC ATT TGA CTC CGT ACC A	[b]				
*trn*L-F	*trn*L-5$^{(UAA)}$F	CGA AAT CGG TAG ACG CTA CG	Taberlet et al. (1991)	94°C, 1min	35×	94°C, 30s;	72°C, 10min
	*trn*F$^{(GAA)}$	ATT TGA ACT GGT GAC ACG AG	Taberlet et al. (1991)			54°C, 30s;	
						72°C, 1min 20s	
ITS	ITS-4	TCC TCC GCT TAT TGA TAT GC	White et al. (1990)	94°C, 1min	35×	94°C, 18s;	72°C, 8min
	ITS-A	GGA AGG AGA AGT CGT AAC AAG G	Blattner (1999)			54°C, 30s;	
						72°C, 1min	

[a] – Internal sequencing primer, [b] – modified internal sequencing primer

References

Blattner, F. R. (1999). Direct amplification of the entire ITS region from poorly preserved plant material using recombinant PCR. *BioTechniques* 27, 1180–1186

Johnson, L. A. and Soltis, D. E. (1995). Phylogenetic Inference in *Saxifragaceae Sensu Stricto* and *Gilia* (*Polemoniaceae*) Using *mat*K Sequences. *Ann. Mo. Bot. Gard.* 82, 149–175. doi:10.2307/2399875

Milne, R. I., Davies, C., Prickett, R., Inns, L. H., and Chamberlain, D. F. (2010). Phylogeny of *Rhododendron* subgenus *Hymenanthes* based on chloroplast DNA markers: between-lineage hybridisation during adaptive radiation? *Plant. Syst. Evol.* 285, 233–244. doi:10.1007/s00606-010-0269-2

Taberlet, P., Gielly, L., Pautou, G., and Bouvet, J. (1991). Universal primers for amplification of three non-coding regions of chloroplast DNA. *Plant Mol. Biol.* 17, 1105–1109. doi:10.1007/bf00037152

White, T., Bruns, T., Lee, S., and Taylor, J. (1990). Amplification and Direct Sequencing of Fungal Ribosomal RNA Genes for Phylogenetics. In *PCR protocols: A Guide to Methods and Applications*, eds. M. A. Innis, D. H. Gelfand, J. J. Sninsky, and T. J. White (Academic Press). 315–322. doi: 10.1016/b978-0-12-372180-8.50042-1
